# Combined chemical transformation and biological transformation of artemisinin: A facile approach to diverse artemisinin derivatives

**DOI:** 10.3389/fchem.2022.1089290

**Published:** 2023-01-24

**Authors:** Xinna Gao, Yue Bai, Peng Sun, Huimin Gao, Lan Yang, Dong Zhang, Yifan Zhao, Yue Ma

**Affiliations:** ^1^ Artemisinin Research Center, Institute of Chinese Meteria Medica, China Academy of Chinese Medical Sciences, Beijing, China; ^2^ School of Graduate Students, Tianjin University of Traditional Chinese Medicine, Tianjin, China

**Keywords:** artemisinin, microbial transformation, *Cunninghamella* genus, anti-malarial activity, bioactive metabolites

## Abstract

**Introduction:** Artemisinin (**1**) is a milestone compound in malaria treatment, and it exhibits a broad scope of bioactivities. Herein, sequential chemo-reduction and biotransformation of artemisinin were undertaken to obtain a series of artemisinin derivatives.

**Methods:** First, 10-deoxyartemisinin (**2**) and 9-ene-10-deoxyartemisinin (**3**) were synthesized after simple handling with boron trifluoride/diethyl ether and sodium borohydride. Then, biotransformation of 10-deoxyartemisinin was conducted with *Cunninghamella echinulata* CGMCC 3.4879 and *Cunninghamella elegans* CGMCC 3.4832, and the transformed products were separated and identified. The antimalarial activity of these products was tested *in vitro* against *Plasmodium falciparum* 3D7.

**Results:** Fifteen metabolites **(4–18)**, including seven novel compounds, were isolated and identified after cultivation. Compounds **2**, **3**, **13**, **15**, **16**, and **18** displayed moderate-to-good antimalarial activity, with a half-maximal inhibitory concentration ranging from 6 to 223 nM.

**Discussion:** This work explored the combination of chemical and biological transformation to develop a co-environmental, efficient, and cost-efficiency synthetic methodology and applied it to synthesize novel derivatives of artemisinin. The association of the two strategies will hopefully provide an abundant source for the development of novel drugs with bioactivities.

## 1 Introduction

Artemisinin (also named qinghaosu, ART) is a legendary antimalarial agent. It was discovered from *Artemisia annua* L. by Tu et al. (1981) in 1972. In the last 50 years, millions of people suffering from malaria have been saved by artemisinin or its derivatives. Artemisinin-based combination therapy (ACT) is a recommended first-line treatment for malaria by the World Health Organization (especially for chloroquine-resistant malaria) (World Health Organization, 2021).

In recent years, parasite clearance for some patients in the Great Mekong Area and parts of Africa has been reported to be delayed after treatment with artesunate for 3 days (Uwimana et al., 2020). According to clinical studies, parasites can be cleared through 7-day treatment of artesunate, but the aforementioned delayed clearance has increased the anxiety about the possibility of resistance to artemisinin (Wang et al., 2019). To prevent the consequences of unpredictable drug resistance, there is an urgent need to explore antimalarial agents, including novel derivatives of artemisinin or other new chemical skeletons.

Microorganism-mediated modification of natural products and bioactive molecules is an efficient route for drug development (Cao et al., 2015). The abundant enzymes found in microorganisms enable hydroxylation, oxidation, reduction, and coupling reactions, with excellent chemo-, regio-, and even stereo-selectivities (Asha and Vidyavathi, 2009). In recent years, numerous microbial-based transformations of artemisinin have been attempted and various microbiological strains have been applied, including those of the genera *Aspergillus*, *Streptomyces*, *Penicillium*, and *Cunninghamella* (Parshikov et al., 2004a; Liu et al., 2006; Goswami et al., 2010; Ponnapalli et al., 2018). However, the number of compounds converted in a single transformation has been modest. In the latest research, four compounds from artemisinin were converted by *Aspergillus niger* in 2022 (Luo et al., 2022). Comparatively, plentiful metabolites were reported in this work.

Our research team has been engaged in the optimization of artemisinin (**1**) for the development of antimalarial drugs. In recent years, we have prepared a series of artemisinin derivatives through fungal-mediated transformations (Bai et al., 2019; Ma et al., 2019; Bai et al., 2021; Zhao et al., 2021). The labile lactone structure of artemisinin is widely perceived to contribute to diminishing the stability of the entire molecule. Herein, the lactone was reduced to methylene to offer 10-deoxyartemisinin (**2**), accompanied by a byproduct: 9-ene-10-deoxyartemisinin (**3**). The 10-deoxyartemisinin was modified by *Cunninghamella* species to obtain structurally divergent metabolites. The antimalarial activity of these generated products against *P. falciparum* (*Pf.*) 3D7 was examined to obtain potential lead compounds for drug development.

## 2 Materials and methods

### 2.1 General experimental procedures

^1^H (600 MHz), ^13^C (150 MHz), and two-dimensional nuclear magnetic resonance (2D-NMR) spectroscopy were undertaken on a spectrometer (AV 600; Bruker, Billerica, MA, United States) with tetramethylsilane as an internal reference. Chemical shifts (*δ*) are given in ppm. Coupling constants (J) are given in hertz. X-ray diffraction was carried out using a diffractometer (D8 Venture; Bruker) with Cu K*α* radiation. Column chromatography was performed with a silica flash column (330 g; Qingdao Marine Chemical Group, Qingdao, China), silica gel (200–300 mesh; Qingdao Marine Chemical Group), and a Chromatorex (FujiSilysia Chemicals, Kasugai, Japan) system. Analytical thin-layer chromatography was carried out on pre-coated silica-gel GF_254_ plates (Qingdao Marine Chemical Group). Water was prepared using a Milli-Q™ system operating at 18.2 MΩ (Millipore, Bedford, MA, United States). Unless stated otherwise, all chemicals were obtained from commercially available sources and were used without further purification.

### 2.2 Synthesis of 10-deoxyartemisinin and 9-ene-10-deoxyartemisinin

**FIGURE 1:**
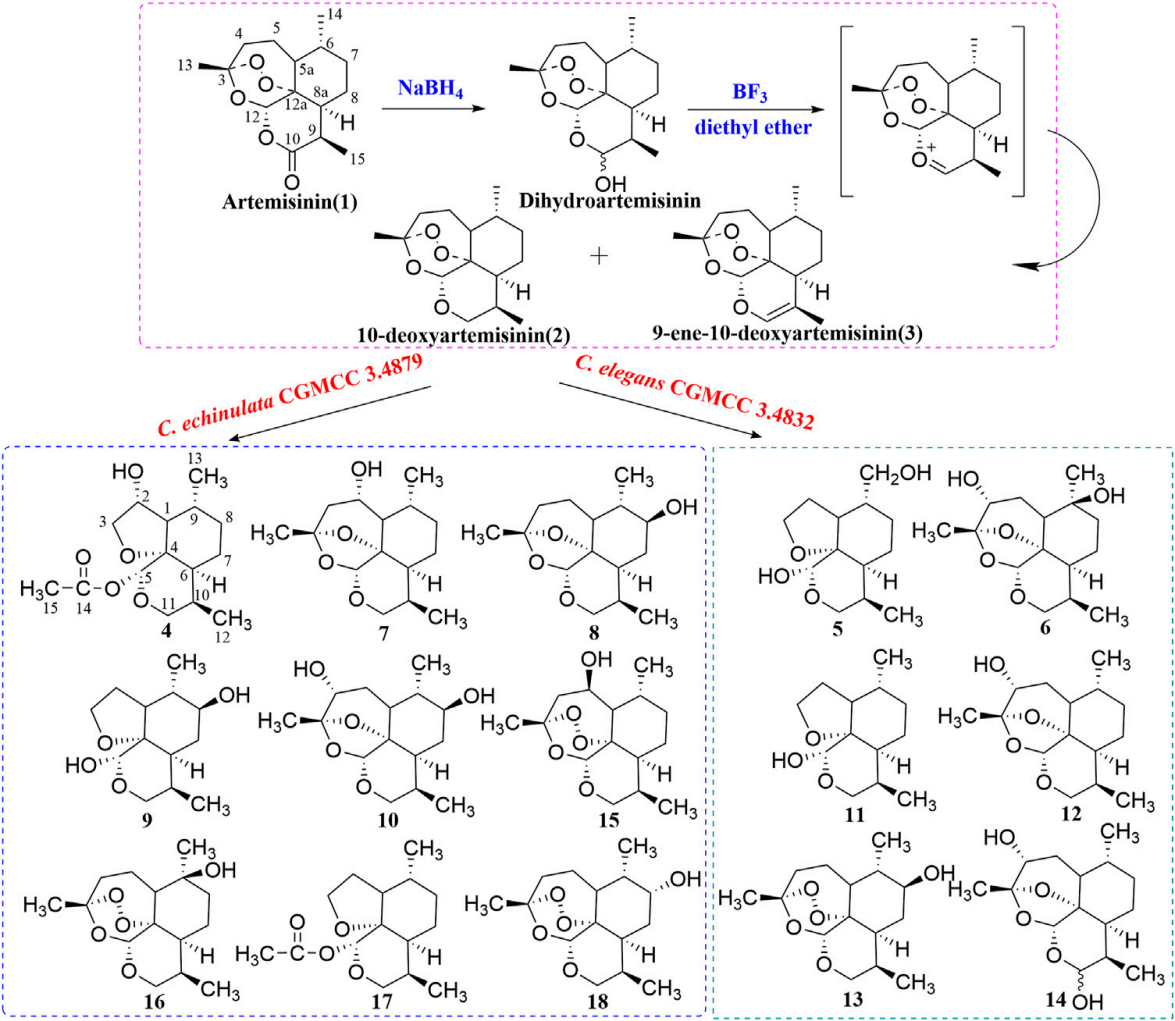
Synthesis route of 10-deoxyartemisinin and its metabolites by *C. elegans* and *C. echinulata*.

Artemisinin (99% by high-performance liquid chromatography (HPLC); batch number, C00120160) was purchased from Kunming Pharmaceutical Group (Kunming, China). Under an inert atmosphere, a solution of artemisinin (2 g) and boron trifluoride/diethyl ether (BF_3_/Et_2_O) (26.4 mL) in dry tetrahydrofuran (THF; 30 mL) at 0 °C was added dropwise to an ice-cooled solution of sodium tetrahydroborate (NaBH_4_; 0.6 g) in dry THF (30 mL). The reaction was carried out for 3 h at 0°C and then heated to reflux for 15 min. The synthetic route is shown in Figure 1. After cooling to room temperature, the reaction mixture was extracted thrice with ether (1:1, *v/v*). The combined organic phase was washed with saturated sodium chloride and dried over anhydrous sodium sulfate (NaSO_4_). The solvent was removed after evaporation *in vacuo* to obtain the crude product. The latter was purified by silica-gel column chromatography. The target product, 10-deoxyartemisinin (**2**, yield 50%), and the byproduct, 9-ene-10-deoxyartemisinin (**3**, yield 22%), were obtained with petroleum ether–ethyl acetate as the eluent. High-resolution-electrospray ionization-mass spectrometry (HR-ESI-MS) and ^1^H-NMR and ^13^C-NMR spectroscopy were used to identify structures. The NMR data of 10-deoxoartemisinin and 9-ene-10-deoxyartemisinin are shown in Table 1 and Table 2. The synthesis scheme is shown in Figure 1.

**TABLE 1:** ^13^C-NMR spectral data (*δ*) for compounds **2**–**18**.

No.	2 *δ*_C_	3 *δ*_C_	4 *δ*_C_	5 *δ*_C_	6 *δ*_C_	7 *δ*_C_	8 *δ*_C_	9 *δ*_C_	10 *δ*_C_	11 *δ*_C_	12 *δ*_C_	13 *δ*_C_	14 *δ*_C_	15 *δ*_C_	16 *δ*_C_	17 *δ*_C_	18 *δ*_C_
1			61.8	50.7				54.5		56.2						55.6	
2			75.7	26.3				28.2		27.4						27.7	
3	104.2	104.1	73.5	68.6	106.4	105.0	106.5	71.1	107.5	69.3	107.3	104.2	108.1	102.4	104.0	68.7	104.2
4	36.3	36.3	82.0	80.3	69.1	44.3	33.1	81.9	69.2	81.2	69.8	36.0	69.6	46.3	35.9	80.4	36.3
5	24.8	24.4	92.5	93.5	39.3	68.0	21.0	95.8	30.3	94.5	30.3	24.6	30.3	69.3	29.8	92.8	24.6
5a	52.2	51.4			42.5	53.1	42.3		42.0		41.8	49.6	41.1	59.4	53.4		44.3
6	37.3	36.7	47.3	45.9	70.3	34.6	41.5	46.6	37.7	46.8	35.0	42.5	35.0	36.9	72.4	47.1	40.7
7	34.0	34.1	20.9	19.7	24.8	34.4	73.8	31.7	74.6	21.0	34.3	73.9	34.2	34.9	39.5	21.0	70.0
8	20.7	29.5	35.6	37.1	17.8	23.0	32.1	78.1	32.8	35.4	23.7	27.6	22.8	20.6	16.7		28.0
8a	44.9	44.2			39.6	39.0	36.8		39.3		39.8	44.1	41.6	44.7	45.1	35.3	37.6
9	28.0	107.7	30.2	28.5	26.1	25.4	25.1	38.7	26.1	30.4	26.4	29.9	33.7	27.8	28.3	30.4	27.3
10	66.2	135.3	28.9	28.8	63.4	63.3	63.3	31.1	64.4	29.8	64.6	66.1	96.7	66.2	66.4	29.6	66.1
11			67.9	66.0				67.9		66.9						67.7	
12	92.1	89.4	12.5	11.8	94.9	95.2	94.9	13.1	95.3	12.8	95.5	91.8	95.4	91.7	93.2	12.7	91.6
12a	80.8	79.1			82.7	81.6	80.7		82.4		83.2	80.0	83.1	80.1	81.3		80.5
13	26.1	16.3	19.8	65.8	19.8	22.7	22.9	17.1	20.5	20.5	20.6	26.0	20.8	25.9	26.1	20.6	26.1
14	20.3	20.5	169.0		27.7	20.8	13.3		15.9		18.7	15.4	18.6	21.3	19.6	169.2	16.5
15	13.1	25.9	21.6		14.0	15.8	15.3		14.2		16.3	13.1	14.6	13.0	13.2	21.6	13.0

**TABLE 2:** ^1^H-NMR spectral data (*δ*) for compounds **2**, **3**, **6–8**, and **10**.

No.	2 *δ*_*H*_ (*J* in Hz)	3 *δ*_*H*_ (*J* in Hz)	6 *δ*_*H*_ (*J* in Hz)	7 *δ*_*H*_ (*J* in Hz)	8 *δ*_*H*_ (*J* in Hz)	10 *δ*_*H*_ (*J* in Hz)
4			3.61–3.55 (m)	2.22 (m)	1.65 (dd, *J* = 13.5, 5.7 Hz)	3.56 (dd, *J* = 9.9, 2.5 Hz)
				1.45 (d, *J* = 3.5 Hz)	1.54–1.47 (m)	
5			1.71–1.63 (m)	3.78 (m)	1.81–1.73 (m)	
			1.45 (m)		1.37–1.24 (m)	
5a			1.60 (m)	1.12 (d, *J* = 6.5 Hz)	1.20 (m)	
6				1.45 (d, *J* = 3.5 Hz)	1.12 (m)	
7			1.71–1.63 (m) 2.02 (m)		3.18 (m, *J* = 10.4, 3,9 Hz)	
8			1.93–1.84 (m)	1.72 (m)	1.37–1.24 (m)	
			1.71–1.63 (m)	1.30 (m)	1.97 (m)	
8a			1.93–1.84 (m)	1.87 (m)	1.97 (m)	2.02 (m)
9	2.57 (m)		2.33–2.25 (m)	2.22 (m)	2.23 (m, *J* = 7.1, 12.3 Hz)	2.32 (m)
10	3.66 (dd, *J* = 12.0, 6.0 Hz)	6.08 (d, *J* = 1.9 Hz)	3.30 (dd, *J* = 11.6, 7.5 Hz)	3.86 (dd, *J* = 11.5, 7.2 Hz)	3.85 (dd, *J* = 11.5, 6.6 Hz)	3.86 (dd, *J* = 11.6, 6.3 Hz)
	3.38 (t, *J* = 12.0 Hz)		3.67 (dd, *J* = 11.6, 5.5 Hz)	3.21 (dd *J* = 11.5, 4.4 Hz)	3.25 (dd, *J* = 11.6, 5.0 Hz)	3.34 (dd, *J* = 11.7, 5.6 Hz)
12	5.13 (s)	5.60 (s)	5.67 (s)	5.08 (s)	5.20 (s)	5.21 (s)
13	1.36 (s)	1.48 (s)	1.50 (s)	1.47 (s)	1.46 (s)	1.56 (s)
14	0.89 (d, *J* = 6.4 Hz)	0.88 (d, *J* = 6.4 Hz)	1.13 (s)	1.12 (d, *J* = 6.5 Hz)	0.96 (d, *J* = 6.2 Hz)	1.00 (d, *J* = 6.4 Hz)
15	0.71 (d, *J* = 7.2 Hz)	1.26 (s)	0.82 (d, *J* = 7.4 Hz)	0.86 (d, *J* = 7.4 Hz)	0.86 (d, *J* = 7.4 Hz)	0.91 (d, *J* = 7.4 Hz)

### 2.3 Preparative biotransformation, extraction, and isolation of metabolites

*C. echinulata* CGMCC 3.4879 and *C. elegans* CGMCC 3.4832 were obtained from the China General Microbiological Culture Collection Center (Beijing, China). Culture was conducted in a medium comprising sabouraud dextrose broth (20 g/L), peptone (10 g/L), and sucrose (15 g/L). Two-stage fermentation was carried out. The substrate (10-deoxyartemisinin) was dissolved in methanol (25 mg/mL) and added to each flask after the second fermentation to reach a final concentration of 0.5 mg/mL. Cultures were incubated at 28 °C and agitated at 180 rpm/min for 14 days. Then, they were filtered and extracted thrice with ethyl acetate at an equal volume. The extract was dried with anhydrous Na_2_SO_4_ and concentrated under a vacuum at 45 °C to provide a residue.

The residue from *C. echinulata* CGMCC 3.4879 was subjected to a silica-gel column chromatography by elution with petroleum ether/ethyl acetate to provide six subfractions (Fr.1–Fr.6). Fractions 1, 2, and 4 were separated by Chromatorex silica-gel column chromatography with petroleum ether/ethyl acetate to obtain compound **12** (200 mg), compound **9** (9 mg), and compound **13** (250 mg), respectively. Fraction 3 was purified by recrystallization from ethyl acetate to provide compound **11** (120 mg). Fractions 5 and 6 were purified by semi-preparative normal-phase HPLC (methanol/water) to gain compound **14** (7 mg) and compounds **4–6** (34, 12, and 6 mg), respectively.

The residue from *C. elegans* CGMCC 3.4832 was subjected to silica-gel column chromatography by elution with petroleum ether/ethyl acetate to provide seven fractions (Fr.1–Fr.7). Fractions 1, 5, and 7 were separated by Chromatorex silica-gel column chromatography with petroleum ether/ethyl acetate to afford compound **17** (50 mg), compound **18** (154 mg), and compound **9** (5 mg), respectively. Fraction 3 was separated by reverse-phase C_18_ column chromatography with methanol/water to obtain compound **15** (10 mg). Fraction 4 was separated by Chromatorex silica-gel column chromatography with petroleum ether/ethyl acetate with additional reverse-phase C_18_ column chromatography to obtain compound **7** (10 mg), compound **8** (4 mg), compound **16** (20 mg), and compound **10** (100 mg).

### 2.4 Identification of compounds

10-Deoxyartemisinin (**2**) (Jung et al., 1990): White acicular crystals (ethyl acetate). HR-ESI-MS *m/z* 291.1624 [M + Na]^+^ (calcd for C_15_H_24_O_4_, 268.1675). ^13^C-NMR data are shown in Table 1. ^1^H-NMR data are shown in Table 2.

9-ene-10-Deoxyartemisinin (**3**) (Xie et al., 2001): White powder (ethyl acetate). HR-ESI-MS *m/z* 289.1425 [M + Na]^+^ (calcd for C_15_H_22_O_4_, 266.1518). ^13^C-NMR data are shown in Table 1. ^1^H-NMR data are shown in Table 2.

**FIGURE 2:**
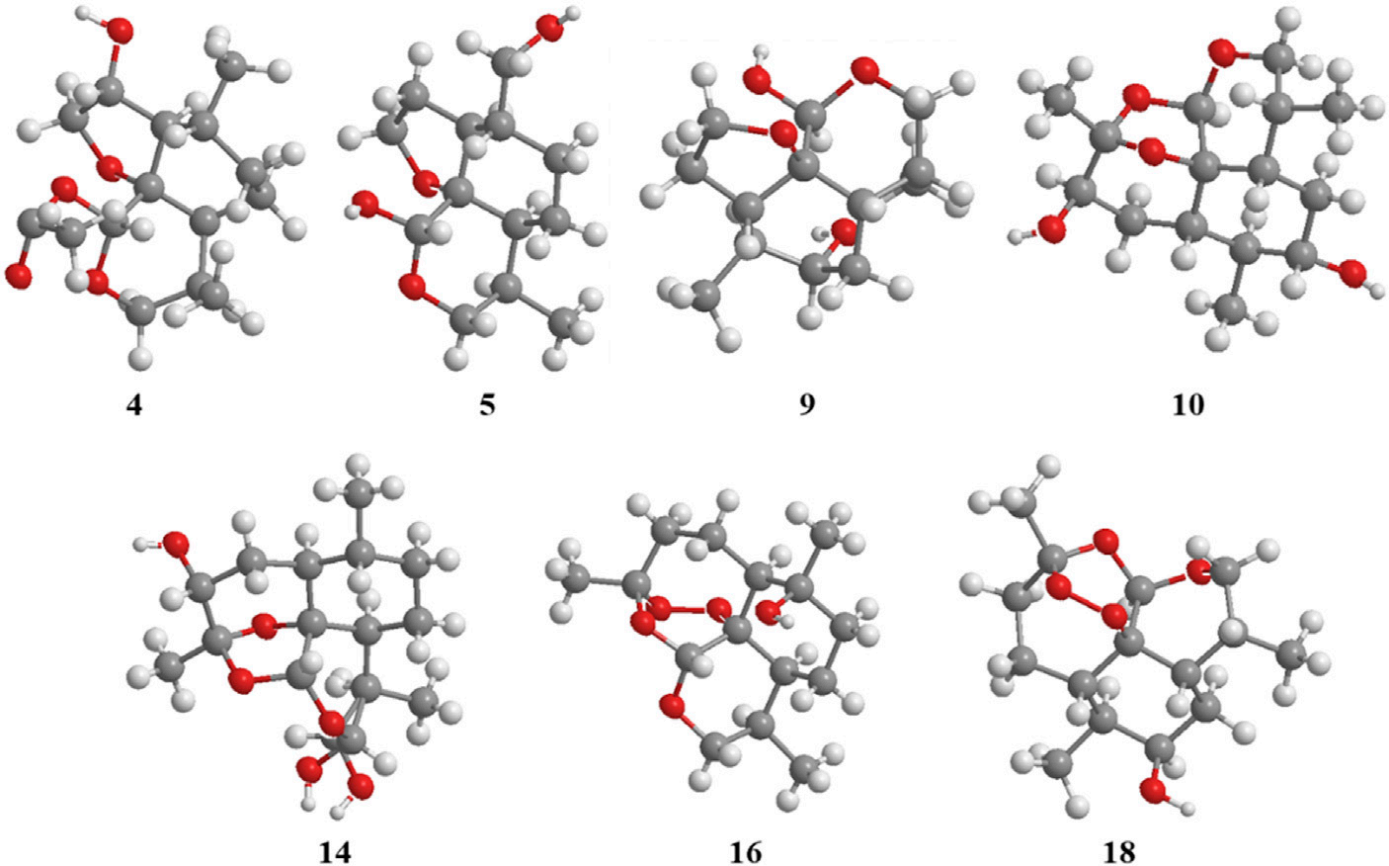
ORTEP drawing of the X-ray structures of compounds 4, 5, 9, 10, 14, 16, and 18.

2*α*-Hydroxy-5*α*-acetoxy-artemethin-Ⅱ (**4**): Colorless, transparent, columnar crystals (ethyl acetate). HR-ESI-MS *m/z* 307.1520 [M + Na]^+^ (calcd for C_15_H_24_O_5_, 284.1624). Crystal data: C_15_H_24_O_5_, M = 284.34, monoclinic system, crystal size is 0.47 mm^3^ × 0.40 mm^3^ × 0.39 mm^3^, a = 13.2788 (6) Å, b = 14.9910 (7) Å, c = 15.8479 (8) Å; *α* = *β* = *γ* = 90.00°, V = 3154.7 (3) Å3, space group P2_1_2_1_2_1_ (NO. 19), T = 273.15 K, Z = 8, Z′ = 2, *μ* (Cu K*α*) = 0.089 mm^−1^, wavelength/Å = 0.71073, R1 = 0.1017, wR (F_2_) = 0.1283. Flack parameter: 0.2 (2). Crystallographic data of compound 4 have been deposited to CCDC (www.ccdc.cam.ac.uk/, number = CCDC 2218207). The structure of a single crystal is shown in Figure 2. ^13^C-NMR data are shown in Table 1. ^1^H-NMR data are shown in Table 4.

5*α*,13-Dihydroxy-artemethin-Ⅱ (**5**): Colorless, transparent, columnar crystals (petroleum ether and acetone). HR-ESI-MS *m/z* 265.1415 [M + Na]^+^ (calcd for C_13_H_22_O_4_, 242.1518). Crystal data: C_13_H_22_O_4_, M = 242.30, monoclinic system, crystal size is 0.47 mm^3^ × 0.40 mm^3^ × 0.39 mm^3^, a = 6.1074 (3) Å, b = 15.6846 (7) Å, c = 6.7830 (3) Å; *α* = *γ* = 90°, V = 634.54 (5) Å3, space group P2_1_ (NO. 4), T = 273.15 K, Z = 2, *μ* (Cu K*α*) = 0.092 mm^−1^, wavelength/Å = 0.71073, R1 = 0.0319 [I > 2*σ* (I)], wR (F_2_) = 0.0875. Flack parameter: 0.2 (2). Crystallographic data of compound 5 have been deposited to CCDC (www.ccdc.cam.ac.uk/, number = CCDC 2218208). The structure of a single crystal is shown in Figure 2. ^13^C-NMR data are shown in Table 1. ^1^H-NMR data are shown in Table 4.

4*α*,6*β*-Dihydroxy-1-deoxy-10-deoxoartemisinin (**6**): White powder (ethyl acetate). HR-ESI-MS 307.1549 [M + Na]^+^ (calcd for C_15_H_24_O_5_, 284.1624). ^13^C-NMR data are shown in Table 1. ^1^H-NMR data are shown in Table 2.

5*α*-Hydroxy-1-deoxy-10-deoxoartemisinin (**7**): White powder (ethyl acetate). HR-ESI-MS 269.1756 [M + H]^+^ (calcd for C_15_H_24_O_4_, 268.1675). ^13^C-NMR data are shown in Table 1. ^1^H-NMR data are shown in Table 2.

7*β*-Hydroxy-1-deoxy-10-deoxoartemisinin (**8**): Yellow powder (ethyl acetate). HR-ESI-MS 269.1754 [M + H]^+^ (calcd for C_15_H_24_O_4_, 268.1675). ^13^C-NMR data are shown in Table 1. ^1^H-NMR data are shown in Table 2.

5*α*,8*β*-Dihydroxy-artemethin-Ⅱ (**9**): Colorless, transparent, needle crystals (ethyl acetate). HR-ESI-MS *m/z* 265.1416 [M + H]^+^ (calcd for C_13_H_22_O_4_, 242.1518). Crystal data: C_13_H_22_O_4_, M = 242.31, triclinic system, crystal size is 0.38 mm^3^ × 0.11 mm^3^ × 0.08 mm^3^, a = 9.5815 (4) Å, b = 10.7290 (5) Å, c = 13.8292 (3) Å; *α* = 79.138 (3)°, *β* = 72.174 (3)°, *γ* = 75.565 (3)°, V = 1300.94 (9) Å3, space group P_1_ (NO. 1), T = 111.6 (3) K, Z = 4, *μ* (Cu K*α*) = 0.738 mm^−1^, R1 = 0.0436 (all data), wR (F_2_) = 0.1088. Flack parameter: 0.06 (11). Crystallographic data of compound 9 have been deposited to CCDC (www.ccdc.cam.ac.uk/, number = CCDC 2218214). The structure of a single crystal is shown in Figure 2. ^13^C-NMR data are shown in Table 1. ^1^H-NMR data are shown in Table 4.

3*α*,7*β*-Dihydroxy-1-deoxy-10-deoxoartemisinin (**10**): Colorless, transparent, columnar crystals (ethyl acetate and acetone). HR-ESI-MS *m/z* 285.1690 [M + Na]^+^ (calcd for C_15_H_24_O_5_, 284.1624). Crystal data: C_15_H_24_O_5_, M = 283.94, triclinic system, crystal size is 0.46 mm^3^ × 0.31 mm^3^ × 0.27 mm^3^, a = 9.3731 (18) Å, b = 12.574 (3) Å, c = 15.842 (3) Å; *α* = 85.623 (6)°, *β* = 81.953 (6)°, *γ* = 89.302 (7)°, V = 89.302 (7) Å3, space group P_1_ (NO. 1), T = 273.15 K, Z = 5, Z' = 5 *μ* (Cu K*α*) = 0.095 mm^−1^, wavelength/Å = 0.71073, R1 = 0.0463 [I > 2*σ* (I)], wR (F_2_) = 0.1078. Flack parameter: −0.05 (19). Crystallographic data of compound 10 have been deposited to CCDC (www.ccdc.cam.ac.uk/, number = CCDC 2218210). The structure of a single crystal is shown in Figure 2. ^13^C-NMR data are shown in Table 1. ^1^H-NMR data are shown in Table 2.

5*α*-Hydroxy-artemethin-Ⅱ (**11**) (Gaur et al., 2014): White powder (ethyl acetate). HR-ESI-MS *m/z* 249.1467 [M + Na]^+^ (calcd for C_13_H_22_O_3_, 226.1569). ^13^C-NMR data are shown in Table 1. ^1^H-NMR data are shown in Table 4.

4*α*-Hydroxy-1-deoxy-10-deoxoartemisinin (**12**) (Parshikov et al., 2004b): White powder (ethyl acetate). HR-ESIMS *m/z* 269.1741 [M + H]^+^ (calcd for C_15_H_24_O_4_, 268.1675). ^13^C-NMR data are shown in Table 1. ^1^H-NMR data are shown in Table 3.

**TABLE 3:** ^1^H-NMR spectral data (*δ*) for compounds **12**–**16** and **18**.

No.	12 *δ*_*H*_ (*J* in Hz)	13 *δ*_*H*_ (*J* in Hz)	14 *δ*_*H*_ (*J* in Hz)	15 *δ*_*H*_ (*J* in Hz)	16 *δ*_*H*_ (*J* in Hz)	18 *δ*_*H*_ (*J* in Hz)
4	3.31 (dd, *J* = 11.6, 5.1 Hz)	2.36 (m)				2.43 (m)
		2.05 (m)				
5	1.88 (m)			3.94 (q, *J* = 7.9 Hz)		
5a				1.27 (dd, *J* = 11.3, 8.0 Hz)		
6	1.27–1.17 (m)			1.58 (m)		
7	1.75 (m)	3.26 (td, *J* = 10.7, 4.4 Hz)				3.88–3.84 (m)
	1.02 (m)					
8	1.88 (m)	1.87 (m)				1.88–1.76 (m)
	1.34–1.27 (m)					
8a	1.97 (d, *J* = 10.3 Hz)	1.64–1.57 (m)		1.64 (m)		2.13–2.02 (m)
9	2.33–2.25 (m)	2.62 (m)	2.46 (m)	2.63 (m)	2.64 (m)	2.71 (m)
10	3.89 (dd, *J* = 11.5, 6.6 Hz)	3.73 (dd, *J* = 11.8, 3.6 Hz)	5.32 (s)	3.70 (dd, *J* = 11.7, 4.2 Hz)	3.75 (dd, *J* = 11.6, 4.0 Hz)	3.78 (dd, *J* = 11.8, 5.0 Hz)
	3.31 (dd, *J* = 11.6, 5.1 Hz)	3.44 (t, *J* = 11.8 Hz)		3.44 (t, *J* = 11.8 Hz)	3.48 (t, *J* = 11.7 Hz)	3.45 (t, *J* = 11.8 Hz)
12	5.18 (s)	5.22 (s)		5.14 (s)	5.61 (s)	5.20 (s)
13	1.56 (s)	1.41 (s)	1.54 (s)	1.41 (s)	1.46 (s)	1.45 (s)
14	0.91 (d, *J* = 7.4 Hz)	1.06 (d, *J* = 6.0 Hz)	0.96 (d, *J* = 7.5 Hz)	1.13 (d, *J* = 6.6 Hz)	1.30 (s)	1.08 (d, *J* = 6.8 Hz)
15	0.87 (d, *J* = 6.4 Hz)	0.78 (d, *J* = 7.2 Hz)	0.88 (d, *J* = 6.4 Hz)	0.76 (d, *J* = 7.2 Hz)	0.82 (d, *J* = 7.2 Hz)	0.80 (d, *J* = 7.2 Hz)

7*β*-Hydroxy-10-deoxoartemisinin (**13**) (Parshikov et al., 2004a): White powder (ethyl acetate). HR-ESI-MS 307.1521 [M + Na]^+^(calcd for C_15_H_24_O_5_, 284.1624). ^13^C-NMR data are shown in Table 1. ^1^H-NMR data are shown in Table 3.

4*α*-Hydroxy-1-deoxydihydroartemisinin (**14**) (Lee et al., 1990): Colorless, transparent, columnar crystals (ethyl acetate). HR-ESI-MS *m/z* 307.1526 [M + Na]^+^ (calcd for C_15_H_24_O_5_, 284.1624). Crystal data: C_15_H_24_O_5_, M = 284.34, monoclinic system, crystal size is 0.25 mm^3^ × 0.21 mm^3^ × 0.13 mm^3^, a = 5.7937 (4) Å, b = 8.5070 (4) Å, c = 29.3066 (17) Å; *α* = *β* = *γ* = 90°, V = 1444.43 (15) Å3, space group P2_1_2_1_2_1_ (NO. 19), T = 294.0 K, Z = 4, *μ* (Cu K*α*) = 0.097 mm^−1^, wavelength/Å = 0.71073, R1 = 0.0585, wR (F_2_) = 0.1157. Flack parameter: 0.0 (12). Crystallographic data of compound 14 have been deposited to CCDC (www.ccdc.cam.ac.uk/. number = CCDC 2218211). The structure of a single crystal is shown in Figure 2. ^13^C-NMR data are shown in Table 1. ^1^H-NMR data are shown in Table 3.

5*β*-Hydroxy-10-deoxoartemisinin (**15**) (Parshikov et al., 2004b): White powder (ethyl acetate). HR-ESI-MS 307.1519 [M + Na]^+^ (calcd for C_15_H_24_O_5_, 284.1624). ^13^C-NMR data are shown in Table 1. ^1^H-NMR data are shown in Table 3.

6*β*-Hydroxy-10-deoxoartemisinin (**16**) (Medeiros et al., 2002): Colorless, transparent, columnar crystals (ethyl acetate). HR-ESI-MS *m/z* 307.1522 [M + Na]^+^ (calcd for C_15_H_24_O_5_, 284.1624). Crystal data: C_15_H_24_O_5_, M = 284.34, monoclinic system, crystal size is 0.24 mm^3^ × 0.21 mm^3^ × 0.10 mm^3^, a = 7.1619 (10) Å, b = 12.1265 (17) Å, c = 16.910 (2) Å; *α* = *β* = *γ* = 90.00°, V = 1468.6 (3) Å3, space group P2_1_2_1_2_1_ (NO. 19), T = 273.15 K, Z = 4, Z′ = 1, *μ* (Cu K*α*) = 0.095 mm^−1^, R1 = 0.0469 [I > 2*σ* (I)], wR (F_2_) = 0.1142. Flack parameter: −0.2 (4). Crystallographic data of compound 16 have been deposited to CCDC (www.ccdc.cam.ac.uk/, number = CCDC 2218212). The structure of a single crystal is shown in Figure 2. ^13^C-NMR data are shown in Table 1. ^1^H-NMR data are shown in Table 3.

5*α*-Acetoxy-artemethin-Ⅱ (**17**) (Khalifa et al., 1995): Colorless, transparent, oily substance (ethyl acetate). HR-ESI-MS *m/z* 291.1573 [M + Na]^+^ (calcd for C_15_H_24_O_4_, 268.1675). ^13^C-NMR data are shown in Table 1. ^1^H-NMR data are shown in Table 4.

**TABLE 4:** ^1^H-NMR spectral data (*δ*) for compounds **4**, **5**, **9**, **11**, and **17**.

No.	4 *δ*_*H*_ (*J* in Hz)	5 *δ*_*H*_ (*J* in Hz)	9 *δ*_*H*_ (*J* in Hz)	11 *δ*_*H*_ (*J* in Hz)	17 *δ*_*H*_ (*J* in Hz)
1			1.36 (m)		1.33 (m)
2	3.52 (t, *J* = 11.9 Hz)	2.20 (m)		1.84 (m)	1.96–1.89 (m)
		2.12–1.90 (m)		2.18 (m)	
3	4.54–4.51 (m)	4.15–4.10 (m)	4.18 (t, *J* = 8.4 Hz)	3.84 (m)	3.91 (q, *J* = 8.0 Hz)
		3.82 (m)	3.84 ( q, *J* = 7.7 Hz)	4.17 (m)	4.27–4.22 (m, 1H)
5	5.92 (s)	4.95 (d, *J* = 8.0 Hz)	5.10 (s)	4.99 (d, *J* = 8.3 Hz)	5.97 (s)
6	1.71 (m)		1.75 (m)		
7	1.96–1.86 (m)		1.58 (m)	1.40–2.00 (m)	
			1.92–1.85 (m)	1.66 (m)	
8		0.89 (d, *J* = 6.6 Hz)	3.24 (dd, *J* = 10.3, 5.8 Hz)	0.86 (m)	1.96–1.89 (m)
		1.85–1.79 (m)		1.87 (m)	
9		2.60 (d, *J* = 8.3 Hz)	1.58 (m)	1.70–1.75 (m)	1.52 (m)
10	2.49–2.43 (m)	2.38–2.32 (m)	2.23 (m)	2.36 (m)	2.45 (m, *J* = 11.9, 5.0 Hz)
11	3.72 (dd, *J* = 11.7, 5.1 Hz)	3.62 (m)	3.58 (dd, *J* = 11.7, 5.1 Hz)	3.43 (d, *J* = 11.5 Hz)	3.52 (t, *J* = 11.8 Hz)
	3.59 (dd, *J* = 8.1, 4.8 Hz)	3.41 (t, *J* = 11.7 Hz)	3.42 (t, *J* = 11.8 Hz, 1H)	3.64 (m)	3.70 (dd, *J* = 11.6, 5.2 Hz)
12	0.78 (d, *J* = 7.1 Hz)	0.71 (d, *J* = 7.1 Hz)	0.76 (d, *J* = 7.1 Hz)	0.73 (d, *J* = 7.2 Hz)	0.76 (d, *J* = 7.1 Hz)
13	1.06 (d, *J* = 6.4 Hz)		1.03 (d, *J* = 6.3 Hz)	0.94 (d, *J* = 6.5 Hz)	0.91 (d, *J* = 6.4 Hz)
15	2.14 (s)				

7*α*-Hydroxy-10-deoxoartemisinin (**18**) (Khalifa et al., 1995): Colorless, transparent, columnar crystals (ethyl acetate). HR-ESI-MS *m/z* 307.1520 [M + Na]^+^ (calcd for C_15_H_24_O_5_, 284.1624). Crystal data: C_15_H_24_O_5_, M = 284.34, triclinic system, crystal size is 0.46 mm^3^ × 0.31 mm^3^ × 0.27 mm^3^, a = 10.2388 (17) Å, b = 14.937 (3) Å, c = 9.8826 (17) Å; *α* = 94.230 (6)°, *β* = 101.038 (6)°, *γ* = 90.123 (5)°, V = 1479.2 (4) Å3, space group P1 (NO. 1), T = 293 (2) K, Z = 4, *μ* (Cu K*α*) = 0.095 mm^−1^, R1 = 0.1021 [I > 2*σ* (I)], wR (F2) = 0.2594. Flack parameter: −0.3 (15). Crystallographic data of compound 18 have been deposited to CCDC (www.ccdc.cam.ac.uk/, number = CCDC 2218213). The structure of a single crystal is shown in Figure 2. ^13^C-NMR data are shown in Table 1. ^1^H-NMR data are shown in Table 3.

### 2.5 Evaluation of antimalarial activity *in vitro*

*Pf*. 3D7 strains were obtained from Professor Chenqijun (Institute of Zoonosis, Jilin University, Jilin, China). *Pf.* 3D7 strains were grown under a gas mixture (5% CO_2_, 5% O_2_, and 90% N_2_). Human erythrocytes were grown at 2% hematocrit. Synchronization was carried out by treatment with 5% D-sorbitol when most parasites were in the “ring” stage. All compounds were prepared in dimethyl sulfoxide and diluted serially in culture medium (100 µL) across the columns of a 96-well tissue-culture plate. Artemisinin was used as a positive control drug. Then, 100 µL of a parasite suspension (1% ring-infected erythrocytes at 4% hematocrit) was added to each well. The plate was incubated under the gas mixture for 72 h at 37 °C. After incubation, 100 µL of lysis buffer (Tris-Cl (1 M), EDTA (0.5 M), 10% saponin, 0.08% Triton X-100, pH 7.5, SYBR™ Green 1, at the recommended dilution of the manufacturer) was added to each well. The plate was agitated for 1.5 h, and fluorescence was measured at an excitation wavelength of 485 nm and emission wavelength of 530 nm. The half-maximal inhibitory concentration (IC_50_) was used to evaluate the anti-malarial action of all compounds.

## 3 Results

### 3.1 Structural elucidation

The two chemically synthesized derivatives, 10-deoxyartemisinin (**2**) and 9-ene-10-deoxyartemisinin (**3**), were obtained following reduction, dehydration, and a second reduction from artemisinin. In addition, 10-deoxyartemisinin (**2**) was employed as the substrate for microbial transformation with *C. elegans* CGMCC 3.4832 and *C. echinulata* CGMCC 3.4879.

Seven new metabolites and eight known metabolites were isolated and characterized unambiguously by various spectroscopy methods (Figure 1). The biotransformation products were identified to be 2*α*-hydroxy-5*α*-acetoxy-artemethin-Ⅱ (**4**), 5*α*,13-dihydroxy-artemethin-Ⅱ (**5**), 4*α*,6*β*-dihydroxy-1-deoxy-10-deoxoartemisinin (**6**), 5*α*-hydroxy-1-deoxy-10-deoxoartemisinin (**7**), 7*β*-hydroxy-1-deoxy-10-deoxoartemisinin (**8**), 5*α*,8*β*-dihydroxy-artemethin-Ⅱ (**9**), and 4*α*,7*β*-dihydroxy-1-deoxy-10-deoxoartemisinin (**10**). The known compounds were determined to be 5*α*-hydroxy-artemethin-Ⅱ (**11**), 4*α*-hydroxy-1-deoxy-10-deoxoartemisinin (**12**), 7*β*-hydroxy-10-deoxoartemisinin (**13**), 4*α*-hydroxy-1-deoxydihydroartemisinin (**14**), 5*β*-hydroxy-10-deoxoartemisinin (**15**), 6*β*-hydroxy-10-deoxoartemisinin (**16**), 5*α*-acetoxy-artemethin-Ⅱ (**17**), and 7*α*-hydroxy-10-deoxoartemisinin (**18**).

Metabolite **4** had a molecular formula of C_15_H_24_O_5_, as deduced from HR-ESI-MS *m/z* of 307.1520 [M + Na]^+^. ^13^C-NMR data suggested one hydroxy carbon signal (*δ*_C_ 75.7) instead of an alkane carbon signal. The hydroxy group was at C-2 on the basis of the data for 5*α*-acetoxy-artemethin-II (Khalifa et al., 1995). The structure was confirmed by X-ray crystallography. The structure of a single crystal is shown in Figure 2. Thus, metabolite 4 was identified as 2*α*-hydroxy-5*α*-acetoxy-artemethin-Ⅱ.

Metabolite **5** had a molecular formula of C_13_H_22_O_4_, as deduced from its HR-ESI-MS *m/z* of 265.1415 [M + Na]^+^. ^1^H-NMR spectra showed −CH_3_ (*δ*_H_ 0.95, 3H) to be substituted by −CH_2_OH (*δ*_H_ 0.94, 2H). ^13^C-NMR spectra showed that *δ*_C_ 20.5 (C-13) shifted to *δ*_C_ 65.8 compared with 5*α*-hydroxy-artemethin-II (Gaur et al., 2014). Thus, the hydroxy group was at C-13. The structure was confirmed by X-ray crystallography. The structure of a single crystal is shown in Figure 2. Thus, metabolite 5 was identified as 5*α*,13-dihydroxy-artemethin-Ⅱ.

**FIGURE 3:**
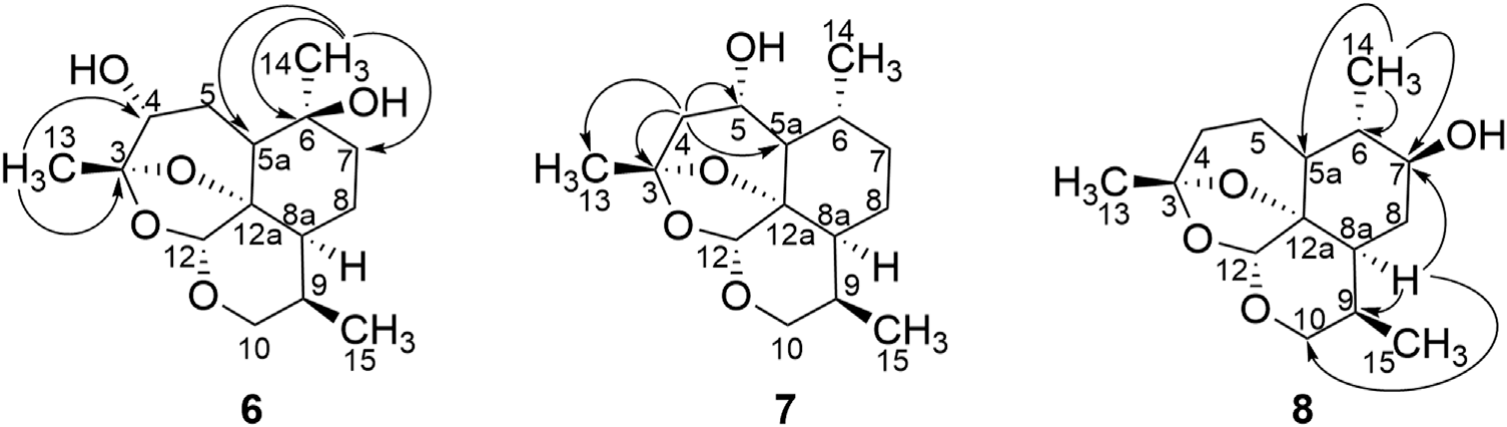
HMBC correlations for compounds 6, 7, and 8.

Metabolite **6** had a molecular formula of C_15_H_24_O_5_, as deduced from its HR-ESI-MS *m/z* of 307.1549 [M + Na]^+^. ^13^C-NMR (150 MHz, CDCl_3_) and distortionless enhancement by polarization transfer (DEPT) spectroscopy showed 15 carbon signals: three methyl, four methylene, five methine, and three quaternary carbon atoms. The low-field shift of C-3 (*δ*_C_ 106.4) together with C-12 (*δ*_C_ 94.9) and C-12a (*δ*_C_ 82.7) compared with those of **2** implied deoxidation of an endoperoxide bridge. Compared with 4*α*-hydroxy-1-deoxy-10-deoxoartemisinin (Parshikov et al., 2004a), a quaternary carbon signal *δ*_C_ 70.3 was found in place of a tertiary carbon signal. Together with a mass shift of 16 Da, the aforementioned information implied one more hydroxy group than that in 4*α*-hydroxy-1-deoxy-10-deoxoartemisinin. Heteronuclear multiple-bond coherence (HMBC) correlation from the methoxy proton (*δ*_H_ 1.13, H-14) to the quaternary carbon (*δ*_C_ 70.3, C-6) confirmed a hydroxyl group at C-6. HMBC correlation for metabolite **6** is shown in Figure 3. Thus, metabolite **6** was identified as 4*α*,6*β*-dihydroxy-1-deoxy-10-deoxoartemisinin.

Metabolite **7** had a molecular formula of C_15_H_24_O_4_, as determined by HR-ESI-MS at *m/z* of 269.1756 [M + H]^+^. ^13^C-NMR (150 MHz, CDCl_3_), and DEPT spectroscopy showed three methyl carbon signals (*δ* 22.7, 20.7, and 15.8), four methylene signals (*δ* 63.3, 44.3, 34.4, and 23.0), six tertiary carbon signals (*δ* 95.2, 67.9, 54.1, 39.0, 34.6, and 25.4), and two quaternary carbon signals (*δ* 105.0 and 81.7). Compared with 4*α*-hydroxy-1-deoxy-10-deoxoartemisinin (Parshikov et al., 2004b), metabolite **7** was indicated to be a monohydroxy of 1,10-deoxy-artemisinin. ^1^H-NMR spectroscopy (600 MHz, CDCl_3_) showed three methyl hydrogen signals: *δ*_H_ 1.47 (s, 3 H, H-13), 1.12 (d, J = 6.5 Hz, 3 H, H-14), and .86 (d, J = 7.4 Hz, 3 H, H-15), which implied that the hydroxyl group may be present at positions 5, 7, or 8. Based on HMBC spectroscopy, *δ*_H_ 2.22 (m, 1H, H-4*α*) and *δ*_H_ 1.45 (d, 1H, J = 3.5 Hz, H-4*β*) were remotely correlated to *δ*_C_ 105.0 (C-3), *δ*_C_ 15.8 (C-13), *δ*_C_ 53.1 (C-5a), and *δ*_C_ 67.9, and the hydroxyl group was suggested to be located at C-5. HMBC correlation for metabolite **7** is shown in Figure 3. Given the characterized hydroxylation C-5 of artemisinin, the structure of metabolite **7** was identified as 5*α*-hydroxy-1-deoxy-10-deoxoartemisinin.

Metabolite **8** had a molecular formula of C_15_H_24_O_4_, as deduced from its HR-ESI-MS *m/z* of 269.1754 [M + H]^+^. ^13^C-NMR (150 MHz, CDCl_3_), and DEPT spectroscopy showed three methyl signals (*δ* 22.9, 15.3, and 13.3), four methylene signals (*δ* 63.3, 33.1, 32.1, and 21.0), six tertiary carbon signals (*δ* 94.9, 73.8, 42.3, 41.5, 36.8, and 25.1), and two quaternary carbon atoms. Compared with ^13^C-NMR spectroscopy of 1-deoxy-10-deoxoartemisinin, *δ*_C_ 73.8 was predicted to be a hydroxyl carbon signal. Metabolite **8** was suggested to be hydroxyl 1-deoxy-10-deoxoartemisinin. HMBC spectroscopy showed a correlation from *δ*_H_ 0.96 (d, J = 6.2 Hz, 3H, H-14) to *δ*_C_ 42.3 (C-5a), *δ*_C_ 41.50 (C-6), and *δ*_C_ 73.8, and a correlation from *δ*_H_ 2.02–1.92 (m, 1 H, H-8a) to *δ*_C_ 25.1 (C-9), *δ*_C_ 63.3 (C-10), *δ*_C_ 32.1 (C-8), and *δ*_C_ 73.8. Thus, the hydroxyl group was suggested to be positioned at C-7. HMBC correlation for metabolite **8** is shown in Figure 3. Based on the distinction between 7*α* (*δ*_C_ 73.5) and 7*β* (*δ*_C_ 68.7) hydroxylated chemical-shift deviation of artemisinin C-7 (*δ*_C_ 33.6), metabolite **8** was finally identified as 7*β*-hydroxy-1-deoxy-10-deoxoartemisinin.

Metabolite **9** had a molecular formula of C_13_H_22_O_4_, as deduced from its HR-ESI-MS *m/z* of 265.1416 [M + H]^+^. ^13^C-NMR spectroscopy showed one more hydroxy carbon signal (*δ*_C_ 78.1) than that for 5*α*-hydroxy-artemethin-II. Combined with its molecular formula, metabolite **9** was predicted to be hydroxylated 5*α*-hydroxy-artemethin-II. The ^1^H-NMR spectrum showed a hydrogen signal *δ*_H_ 3.24 (dd, J = 10.3, 5.8 Hz, 1H), which indicated that the hydroxy group was at position C-8. The structure of metabolite **9** was confirmed by X-ray crystallography. The structure of a single crystal is shown in Figure 2. Thus, metabolite **9** was identified as 5*α*,8*β*-dihydroxy-artemethin-Ⅱ.

Metabolite **10** had a molecular formula of C_15_H_24_O_5_, as deduced from its HR-ESI-MS *m/z* of 285.1690 [M + Na]^+^. ^13^C-NMR spectroscopy indicated that metabolite **10** was a two hydroxy of 1-deoxy-10-deoxoartemisinin, of which hydroxy carbon signals were at *δ*_C_ 69.2 and *δ*_C_ 74.6. Compared with 4*α*-hydroxy-1-deoxy-10-deoxoartemisinin, ^1^H-NMR spectroscopy indicated that the hydroxy groups were located at C-4 and C-7. Finally, the structure of metabolite **10** was confirmed by X-ray crystallography. The structure of a single crystal is shown in Figure 2. Thus, metabolite **10** was identified as 4*α*,7*β*-dihydroxy-1-deoxy-10-deoxoartemisinin.

### 3.2 Antimalarial activity *in vitro*

**FIGURE 4:**
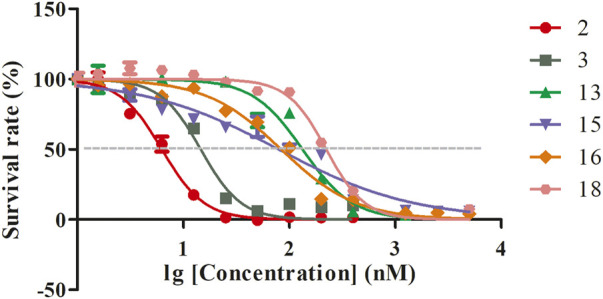
The IC_50_ curves of compounds 2, 3, 13, 15, 16, and 18.

The positive control drug (artemisinin) exhibited *in vitro* antimalarial activity against *Pf.* 3D7, with an IC_50_ (50% inhibition concentration) value of 11 nM. The *in vitro* antimalarial activity against *Pf.* 3D7 of compounds **2**, **3**, **13**, **15**, **16**, and **18** was indicated by IC_50_ values (nM) of 6, 15, 133, 79, 84, and 223 (Figure 4). The other compounds did not show activity against *Pf.* 3D7.

## 4 Discussion

Artemisinin is an unusual sesquiterpene lactone possessing an endoperoxide moiety. The sesquiterpene lactone endoperoxide structure of artemisinin is the pharmacophore characteristic element. The partial resistance of artemisinin refers to a delay in the clearance of malaria parasites from the bloodstream following treatment with ACT. This class of compounds with parent nucleus structures is characterized by a relatively complex metabolic process *in vivo*, which was considered to be partly responsible for the drug resistance phenomenon by some perspectives. Exploring the associated hydroxylation derivatives, especially hydroxylation, which is highly similar to the *in vivo* disposal process of artemisinin, is an effective approach to discover novel agents with higher activities.

Structural modification of artemisinin to improve its solubility, stability, and bioavailability has been a research “hotspot” in medicinal chemistry since its discovery. In recent years, the need for novel antimalarial drugs to prevent unpredictable drug tolerance has been urgent. 10-Deoxyartemisinin was first synthesized by Jung in 1989, and it was shown to be more stable in gastric acid and more efficacious than artemisinin. The synthesis of 10-deoxyartemisinin has been previously examined in detail, and in this work the reaction conditions were optimized, including temperature, duration, and solvent. As a result, a direct one-step reduction of the carbonyl function of artemisinin into 10-deoxyartemisinin was successfully achieved by NaBH_4_ in the presence of BF_3_/Et_2_O, with a yield of 50%. Moreover, 9-ene-10-deoxyartemisinin was isolated as a by-product, and it demonstrated similar antimalarial activity to that of artemisinin. Subsequently, 10-deoxyartemisinin was chosen as the substrate for microbial transformation.

*Cunninghamella* species are commonly used models for microbial transformation. Numerous research studies have confirmed that *Cunninghamella* functioned with outstanding hydroxylation ability, which was responsible for the cytochrome P450 activity of fungus (Wang et al., 2000; Asha and Vidyavathi, 2009). In addition, some research studies found that the cytochrome P450 in *Cunninghamella* species belongs to the CYP5I family, and the role of these enzymes was confirmed as the function of CYP3A4 enzyme in mammal metabolism (Dube et al., 2016). Thus, *Cunninghamella* was also employed to transform xenobiotics for the simulation of phase I (oxidative) and phase II (conjugative) metabolism (Zhang et al., 1996). In this work, two strains were chosen based on our previous study on the biotransformation of artemisinin (Ma et al., 2019) and dihydroartemisinin (unpublished data). The activity evaluation of the microbial transformation products showed that the antimalarial activity of the C-5 hydroxylated product was better than others. Though metabolites **13** and **18** were both hydroxylated products at the C-7 position, the antimalarial activity of the *β*-OH product exhibited two times better efficiency than the *α*-OH product. Unfortunately, hydroxylated products at C-5, C-6, and C-7 positions of 10-deoxyartemisinin led to attenuated antimalarial activities. Nevertheless, hydroxylation could improve the solubility of the compound, while also providing the possibility for further functionalization. Derivatives with a reduced peroxide bridge exhibited negligible antimalarial activity, a finding that is in accordance with previous reports (O’Neill et al., 2010). The synergistic effect of these products on malarial treatment and other bioactivities needs to be further studied.

## 5 Conclusion

In summary, seventeen artemisinin derivatives, including seven novel compounds (**4–10**) and ten known compounds, were isolated and identified through combined chemical and biological transformation. This protocol provided a highly efficient and divergent translation strategy for artemisinin. The pharmacological activities of the generated products were evaluated, and some derivatives displayed good antimalarial activity, which inspired us to conduct a comprehensive druggability study. The novel products with divergent structural moieties provided promising candidates for further bio-evaluation and drug development.

## Data Availability

The data presented in the study are deposited in the article/Supplementary Material. The crystallographic data presented in the study are deposited in www.ccdc.cam.ac.uk/, accession numbers CCDC 2218207 (M4), 2218208 (M5), 2218214 (M9), 2218210 (10), 2218211 (14), 2218212 (M16), 2218213 (M18).
