# Supplementary figures and images for: Combined chemical transformation and biological transformation of artemisinin: A facile approach to diverse artemisinin derivatives
Source: Front Chem. 2023 Jan 24;10:1089290. doi: 10.3389/fchem.2022.1089290 (PMC9902651; doi:10.3389/fchem.2022.1089290)

TQHS.2.fid  
C13CPD CDCl3 D:\nmrsu 11

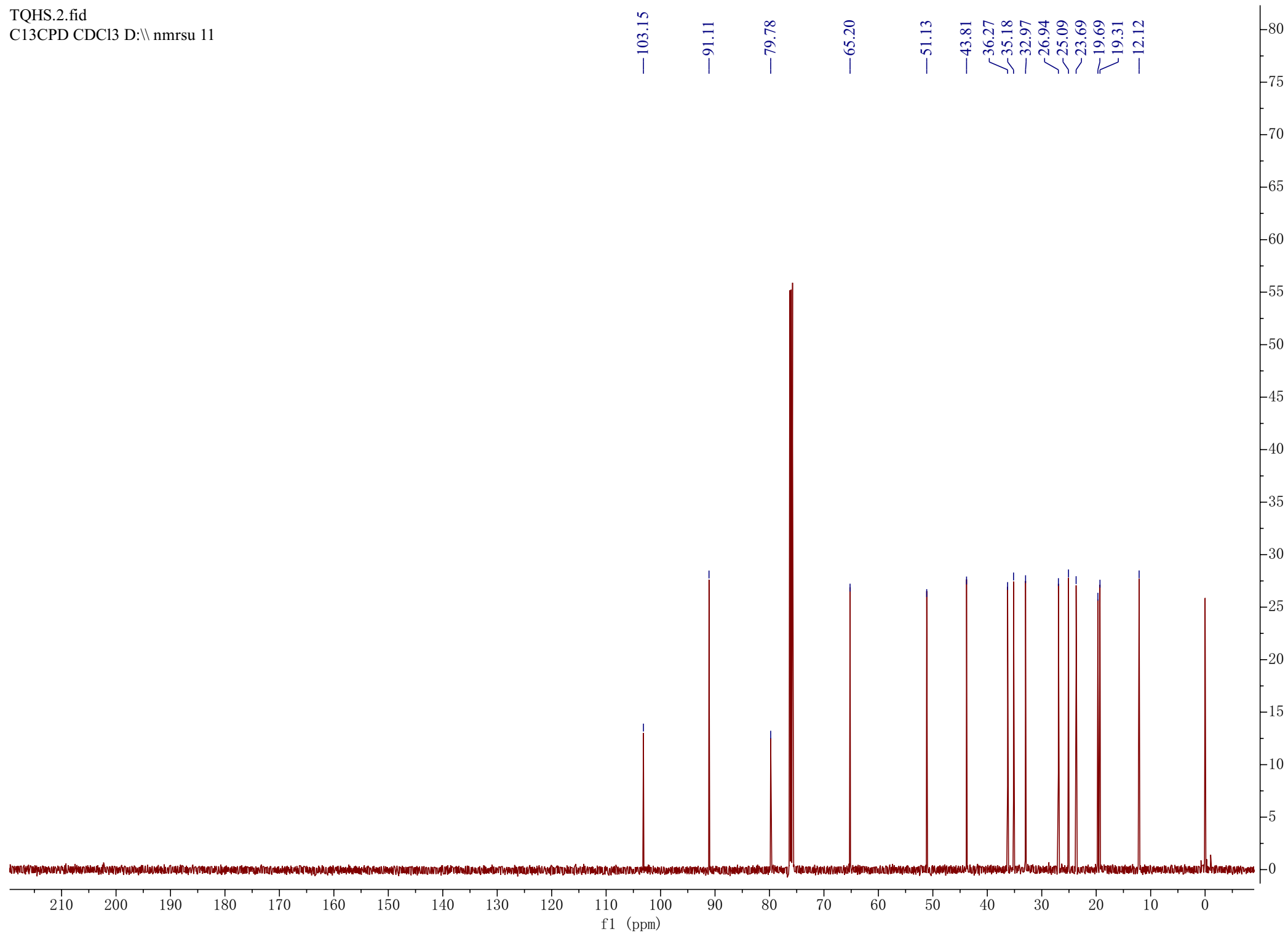

Supplement: Supplementary file 1 [file DataSheet1.ZIP › Supplementary Materials/Figure S1.C-NMR of Metabolite 2.pdf]

m120910.2.fid

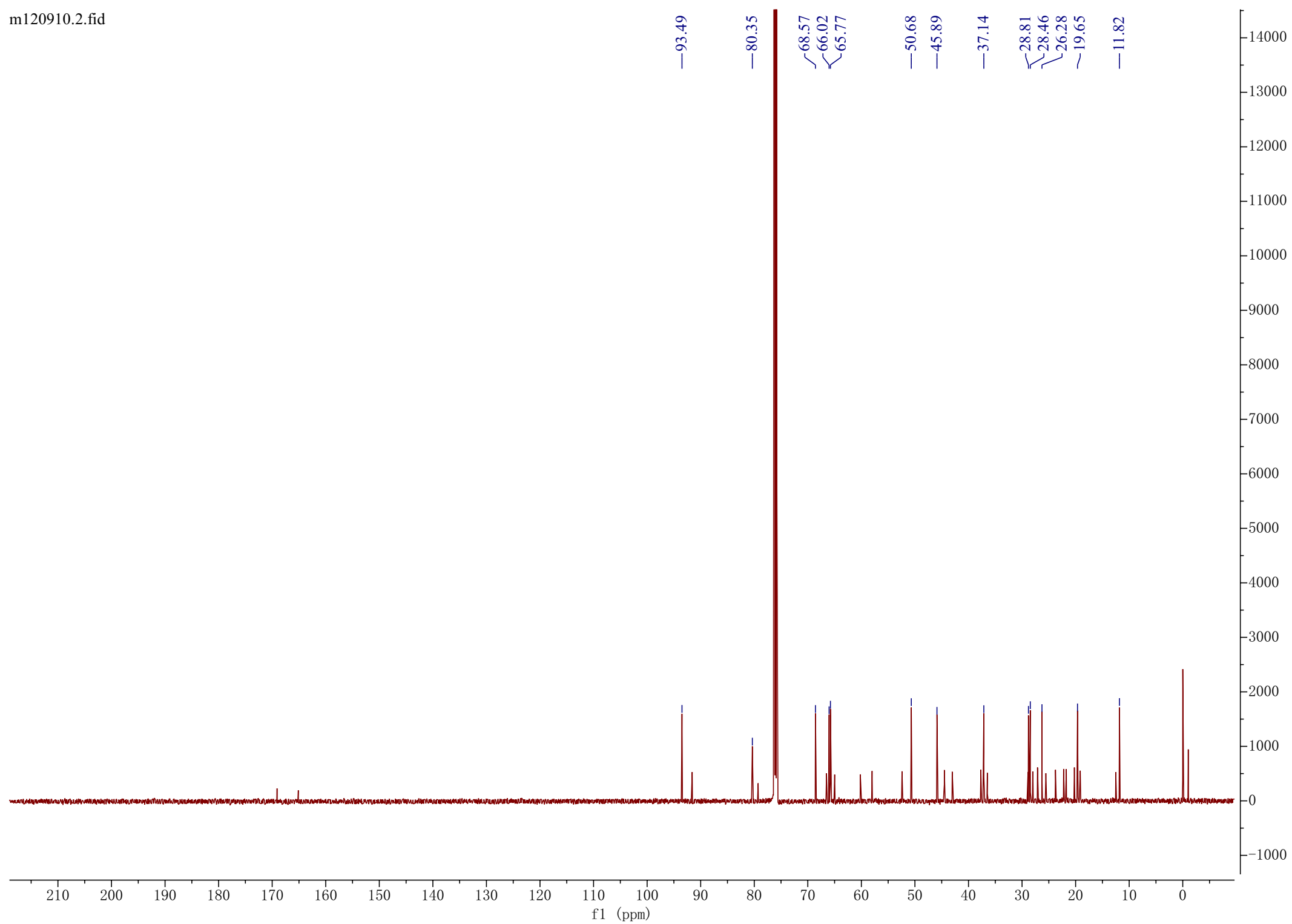

Supplement: Supplementary file 1 [file DataSheet1.ZIP › Supplementary Materials/Figure S10.C-NMR of Metabolite 5.pdf]

m120910.1.fid

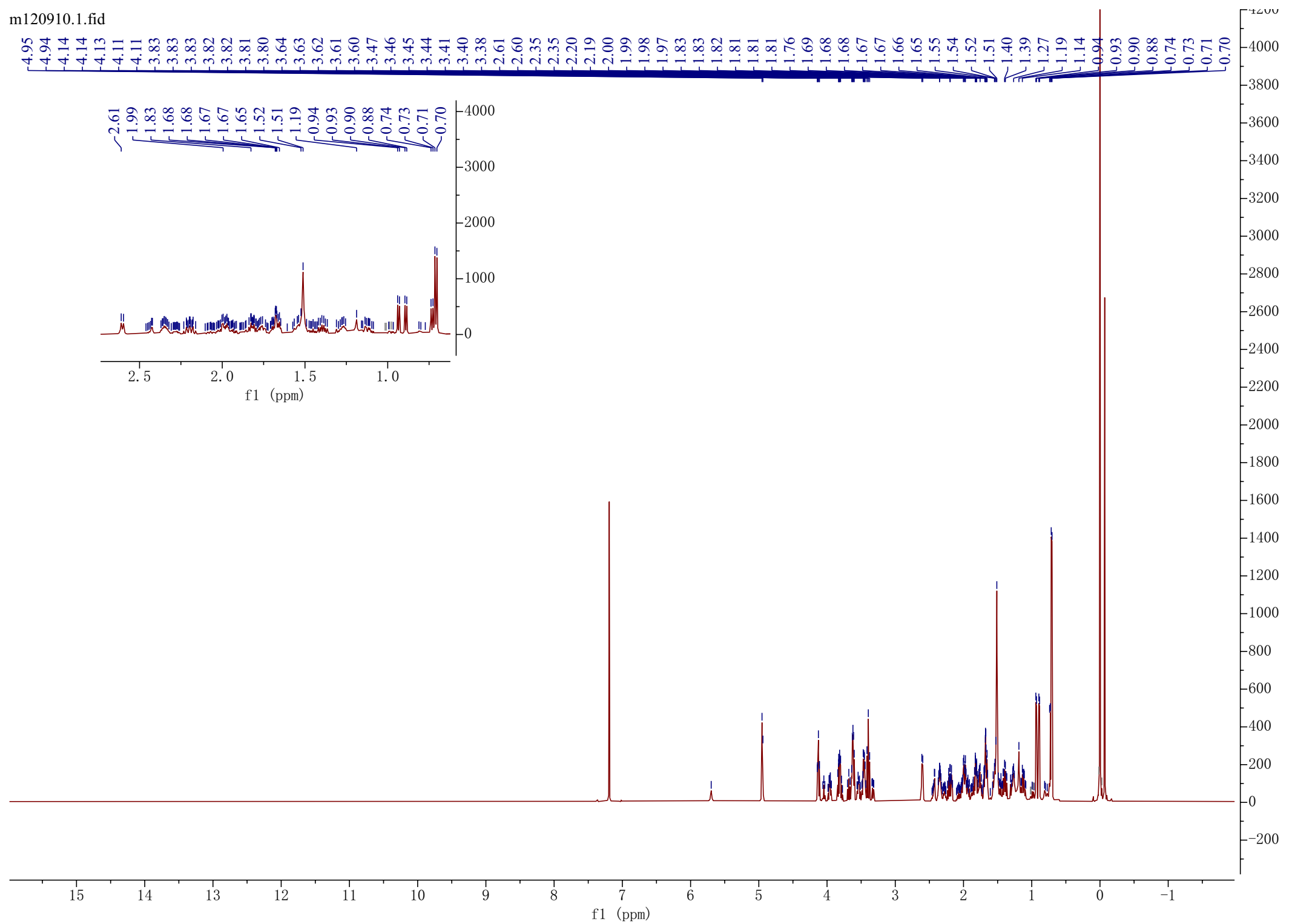

Supplement: Supplementary file 1 [file DataSheet1.ZIP › Supplementary Materials/Figure S11.H-NMR of Metabolite 5.pdf]

1

20200928\_BY\_M12 426 (3.183) AM2 (Ar,22000.0,556.28,0.00,LS 10)

1: TOF MS ES+  
1.48e5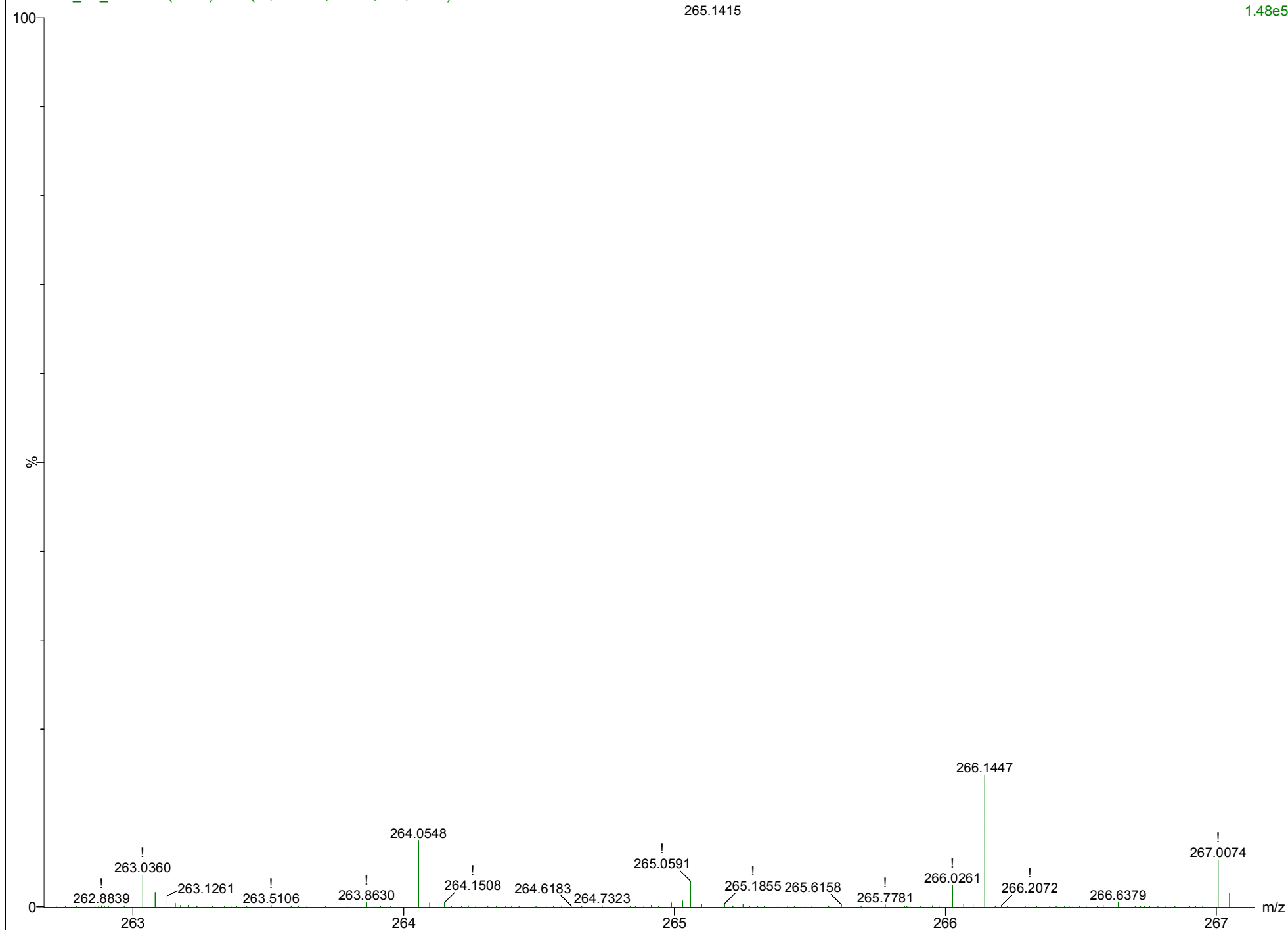

Supplement: Supplementary file 1 [file DataSheet1.ZIP › Supplementary Materials/Figure S12.HR-ESI-MS of Metabolite 5.pdf]

m130910.2.fid

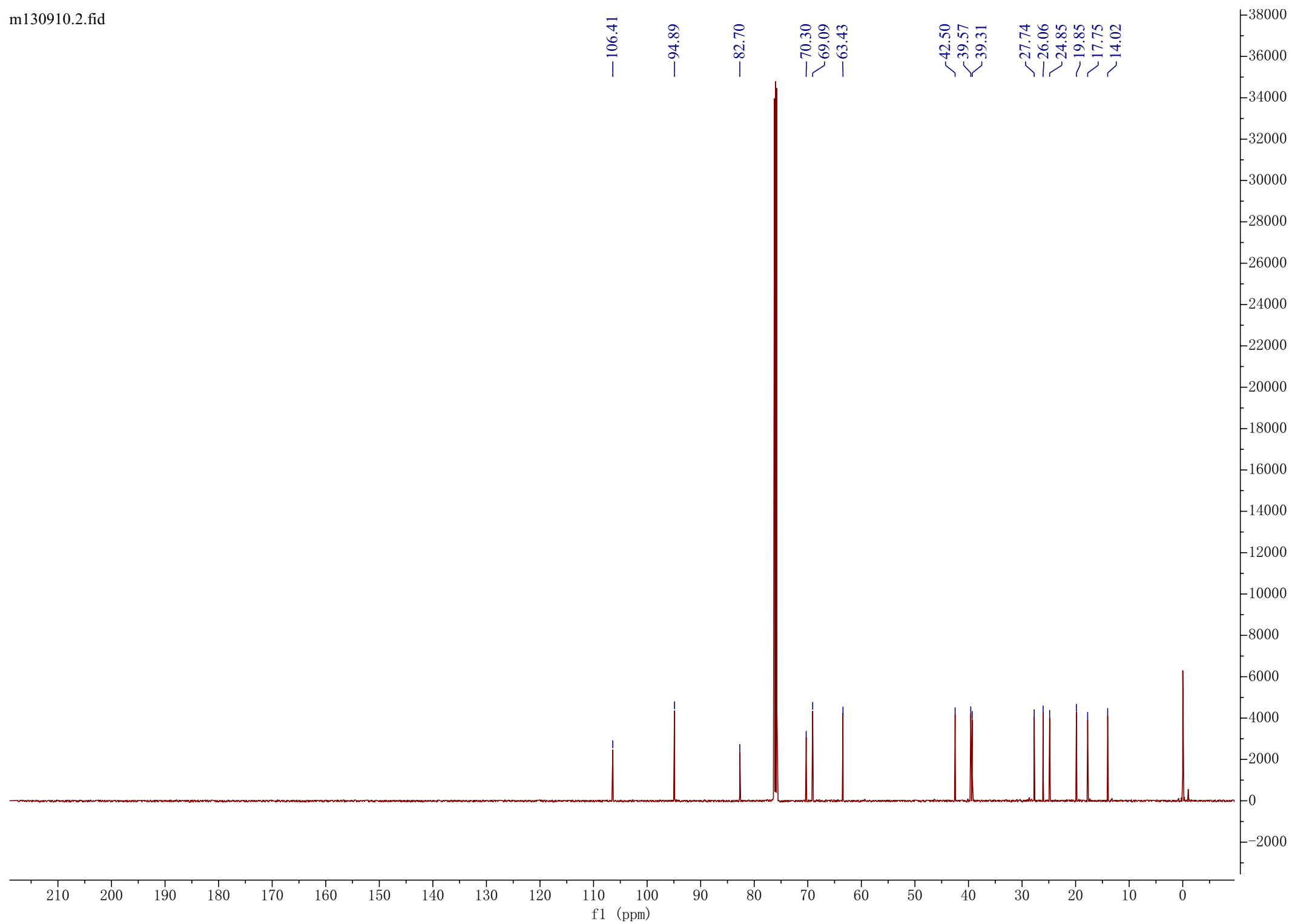

Supplement: Supplementary file 1 [file DataSheet1.ZIP › Supplementary Materials/Figure S13.C-NMR of Metabolite 6.pdf]

m130910.1.fid

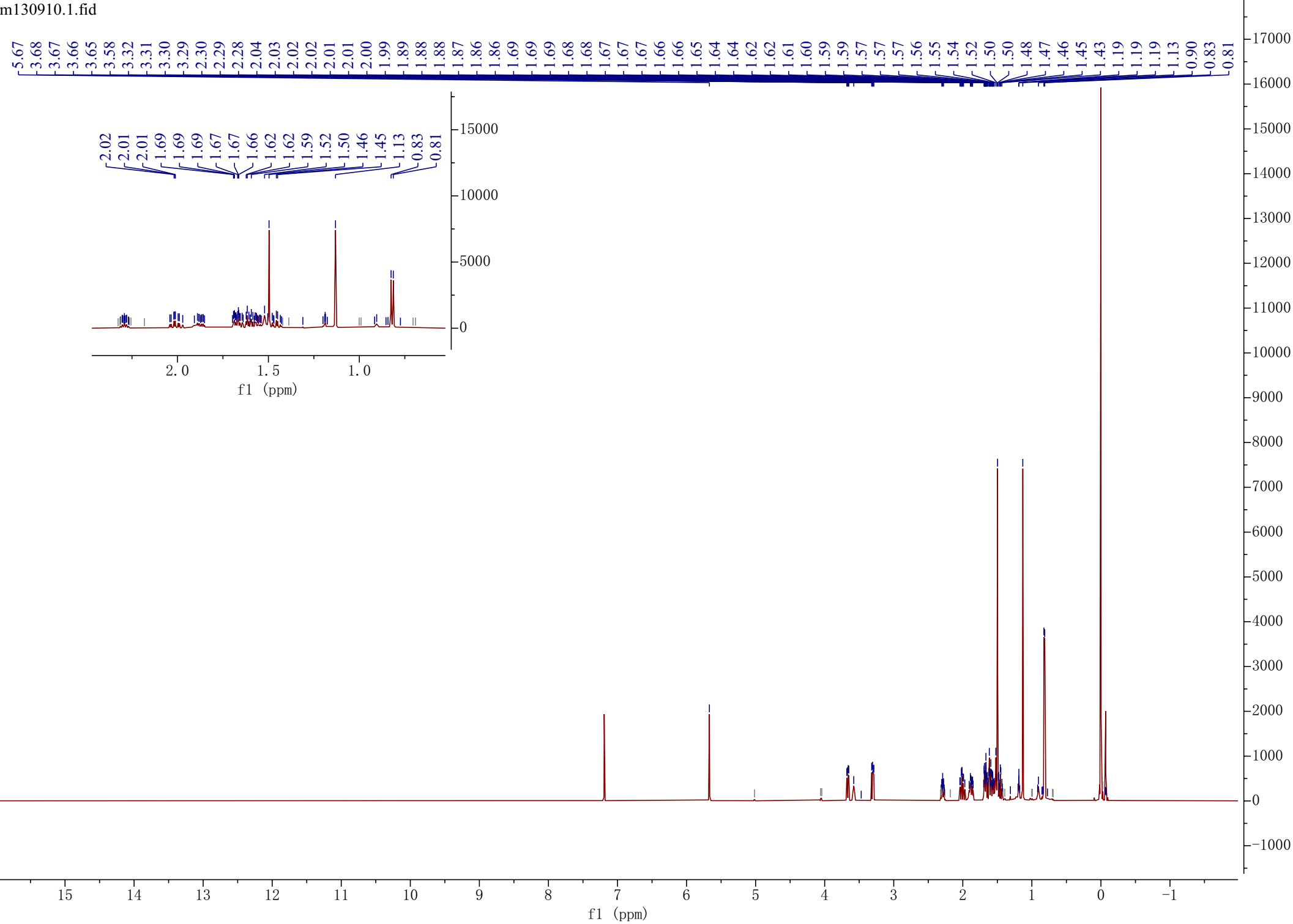

Supplement: Supplementary file 1 [file DataSheet1.ZIP › Supplementary Materials/Figure S14.H-NMR of Metabolite 6.pdf]

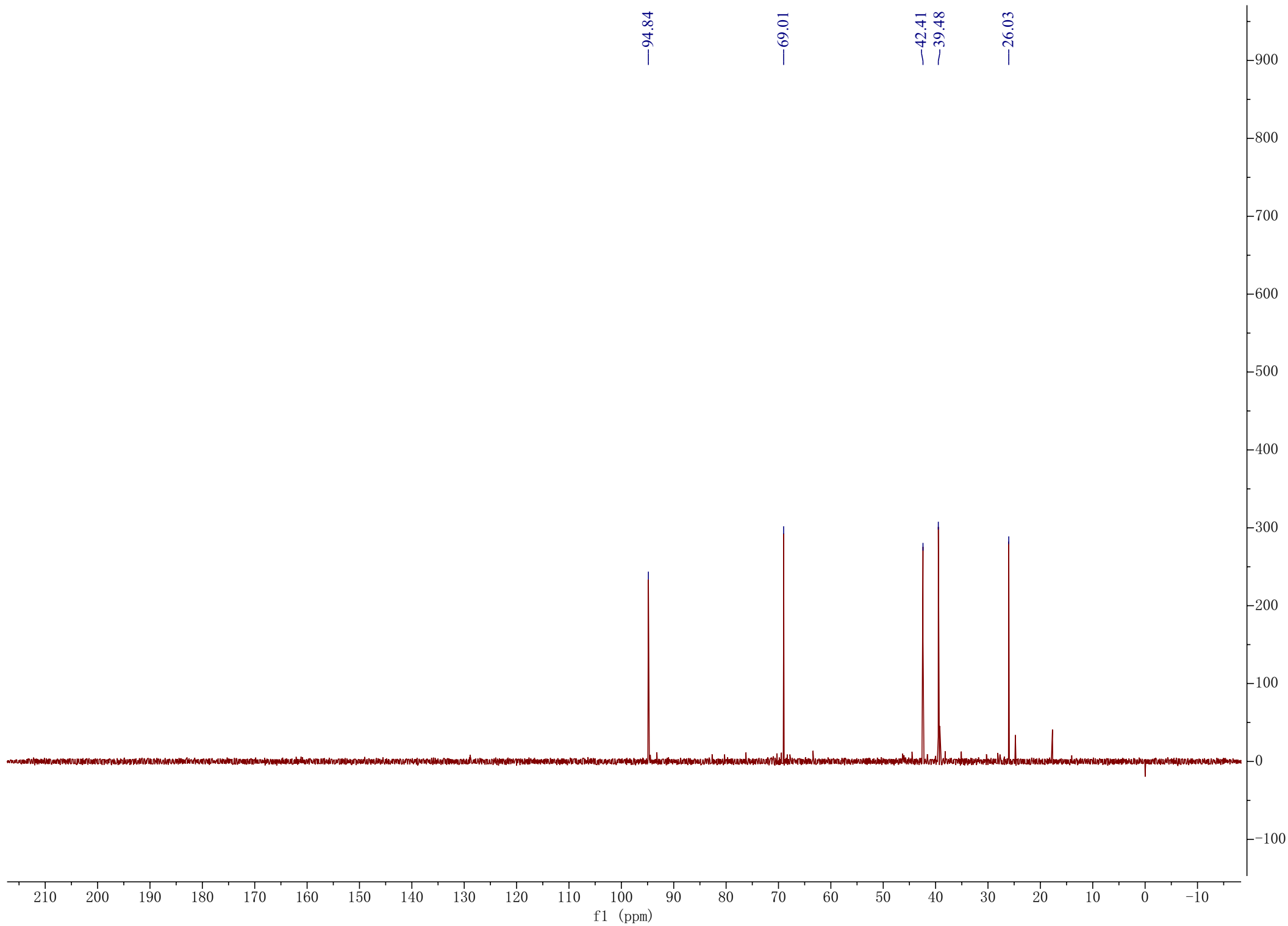

Supplement: Supplementary file 1 [file DataSheet1.ZIP › Supplementary Materials/Figure S15.DEPT(90) of Metabolite 6.pdf]

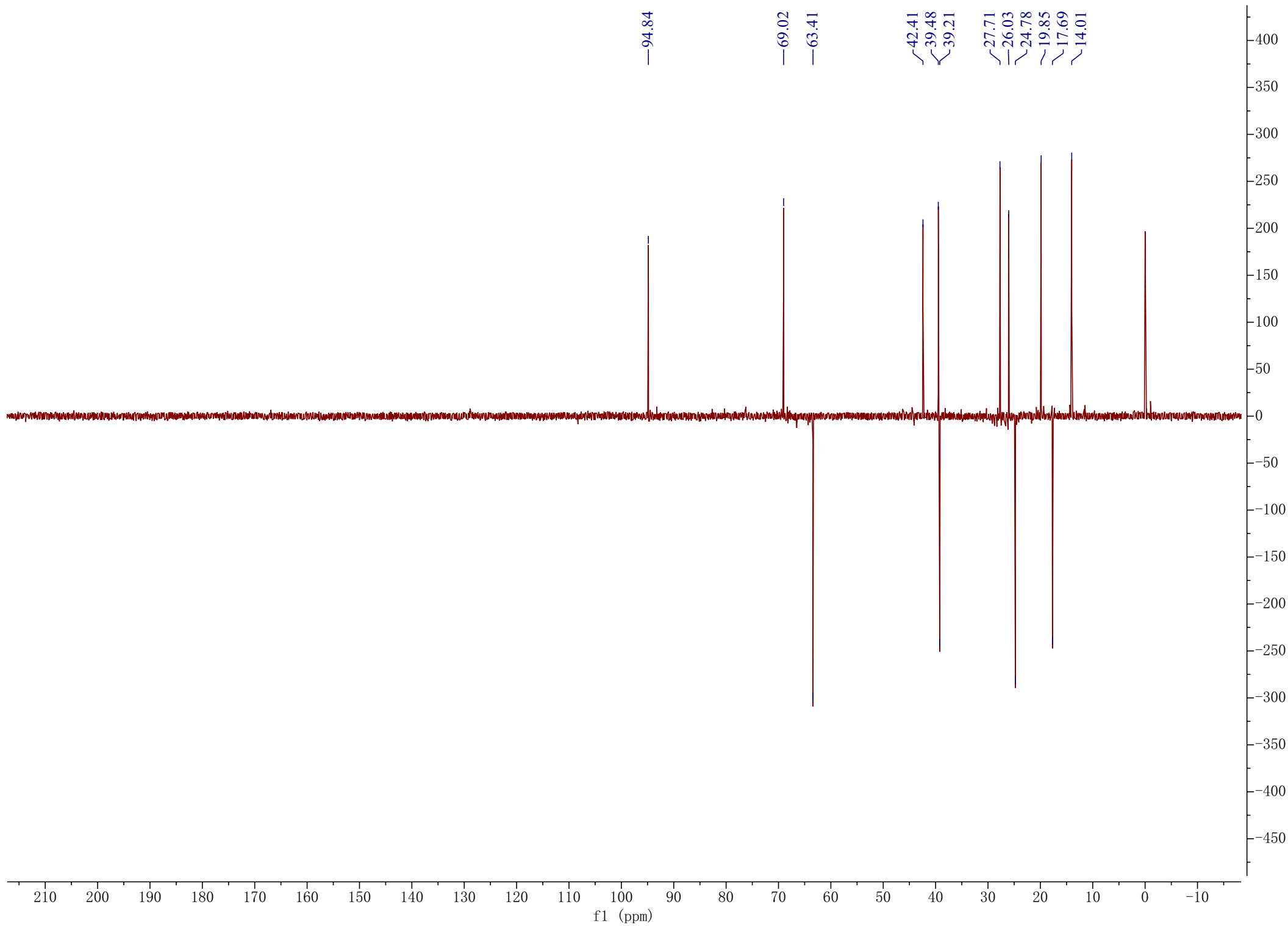

Supplement: Supplementary file 1 [file DataSheet1.ZIP › Supplementary Materials/Figure S16.DEPT(135) of Metabolite 6.pdf]

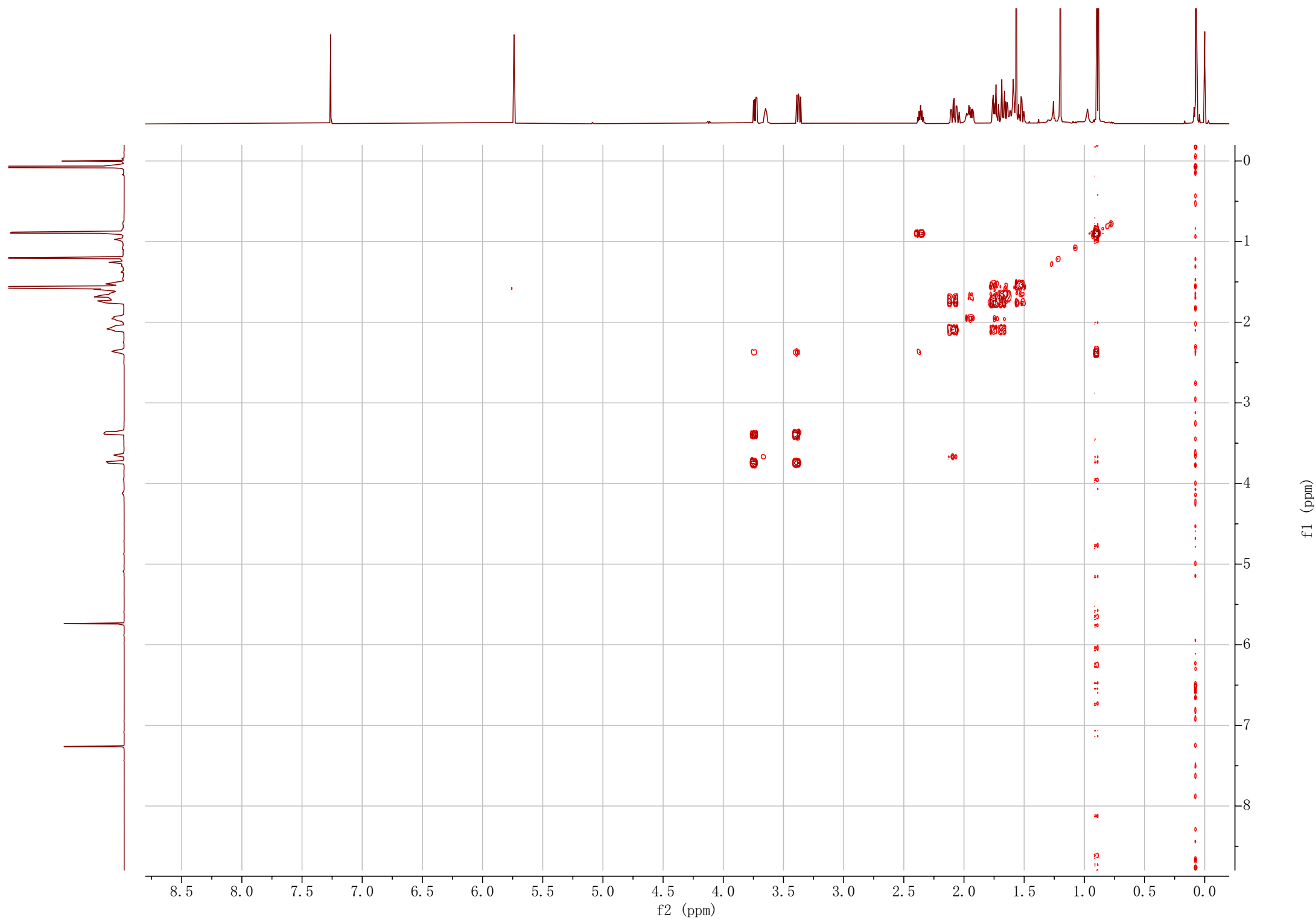

Supplement: Supplementary file 1 [file DataSheet1.ZIP › Supplementary Materials/Figure S17.H-H COSY of Metabolite 6.pdf]

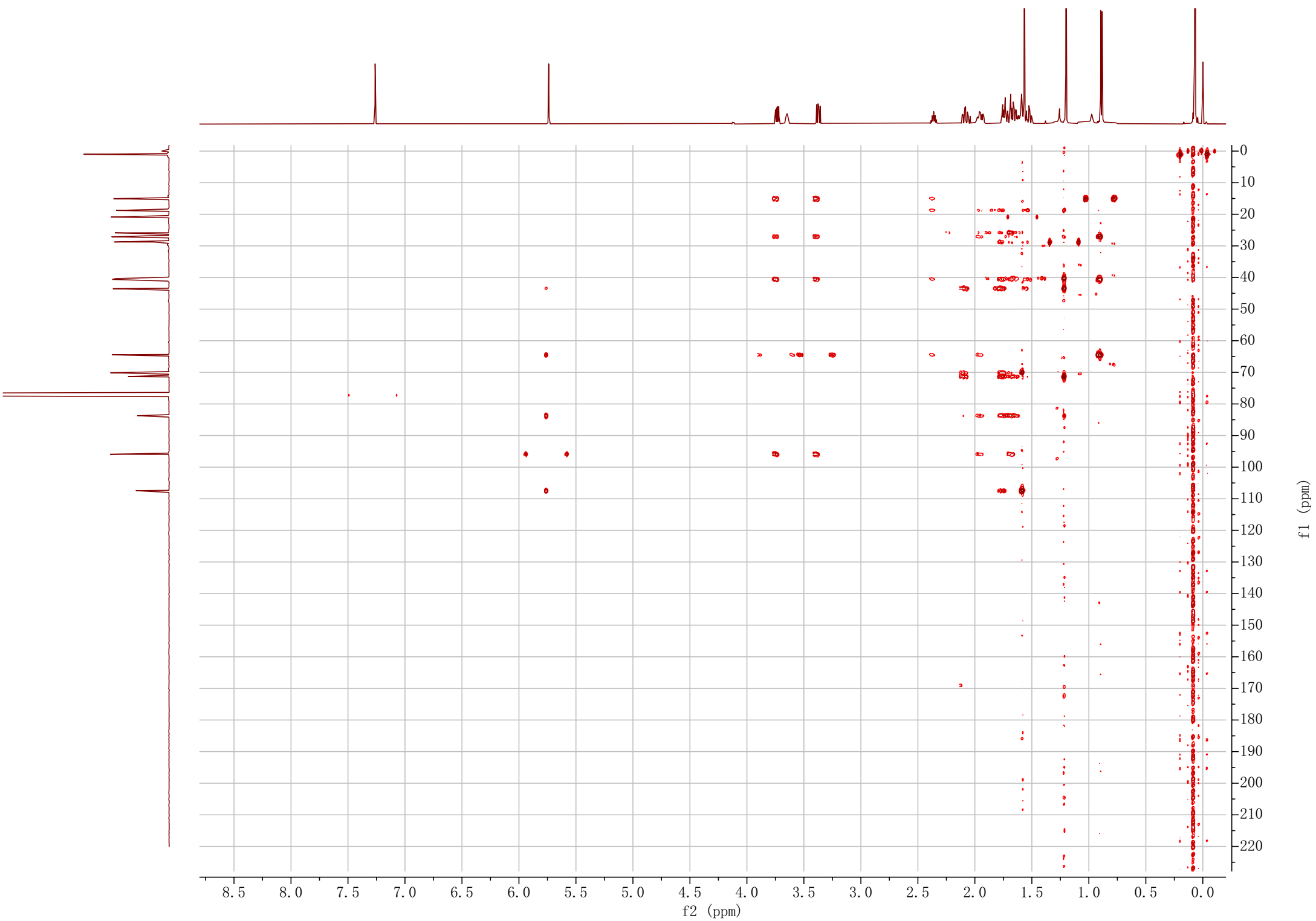

Supplement: Supplementary file 1 [file DataSheet1.ZIP › Supplementary Materials/Figure S18.HMBC of Metabolite 6.pdf]

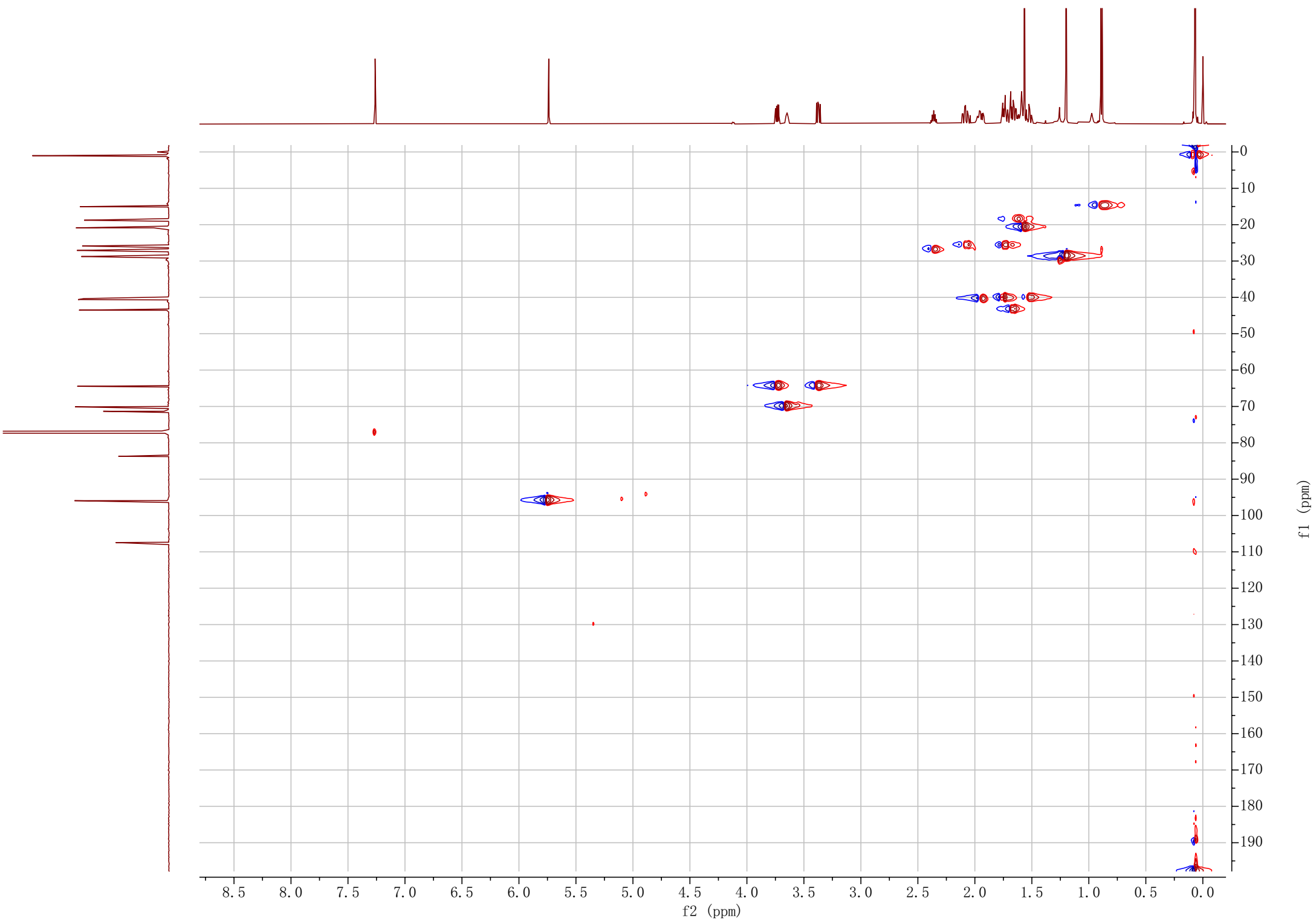

Supplement: Supplementary file 1 [file DataSheet1.ZIP › Supplementary Materials/Figure S19.HSQC of Metabolite 6.pdf]

TQHS.1.fid  
PROTON CDCl3 D:\nmrsu 11

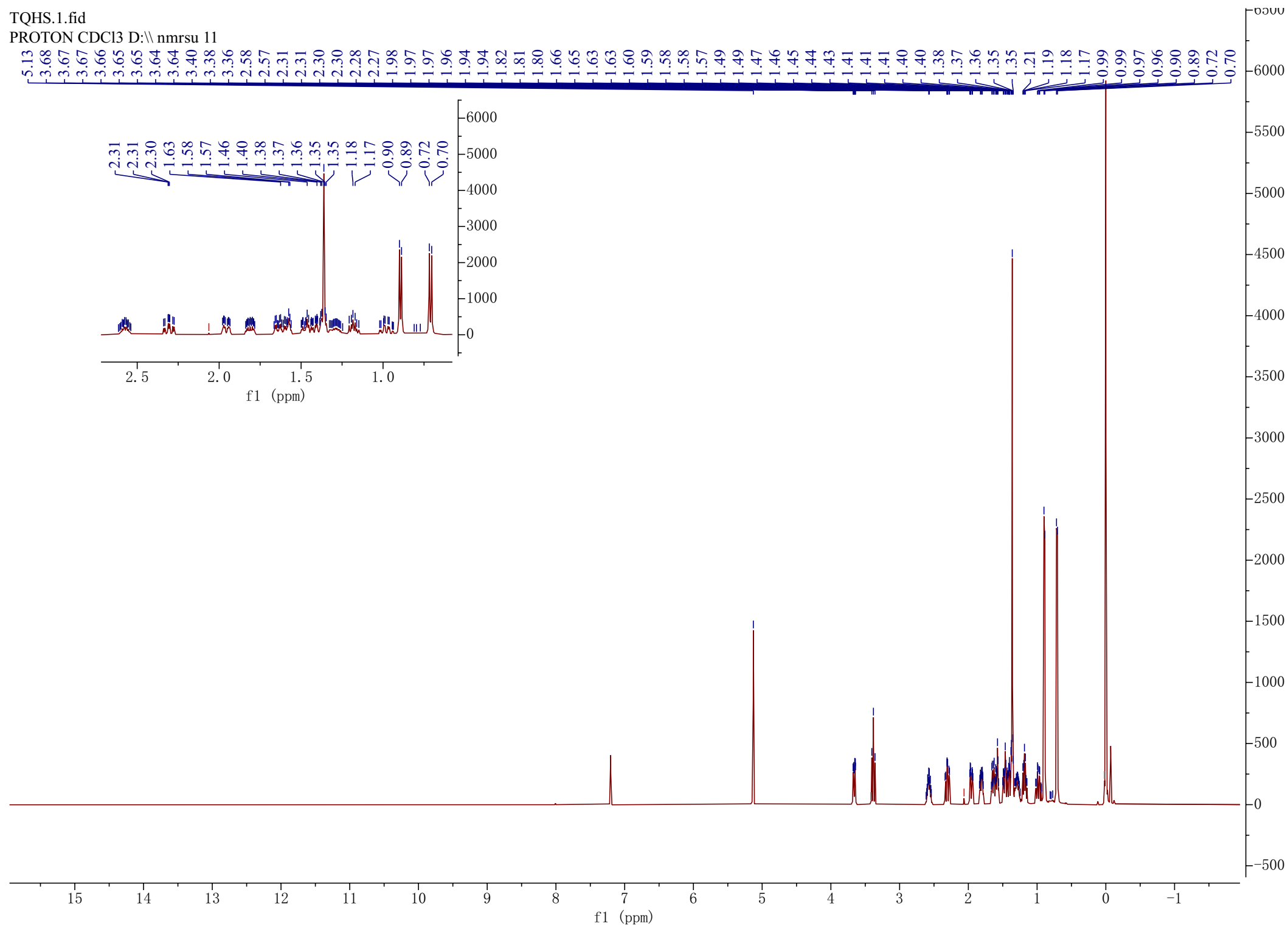

Supplement: Supplementary file 1 [file DataSheet1.ZIP › Supplementary Materials/Figure S2.H-NMR of Metabolite 2.pdf]

5

BY\_20200107M13P 362 (2.710) AM2 (Ar,22000.0,556.28,0.00,LS 10)

1: TOF MS ES+  
7.93e6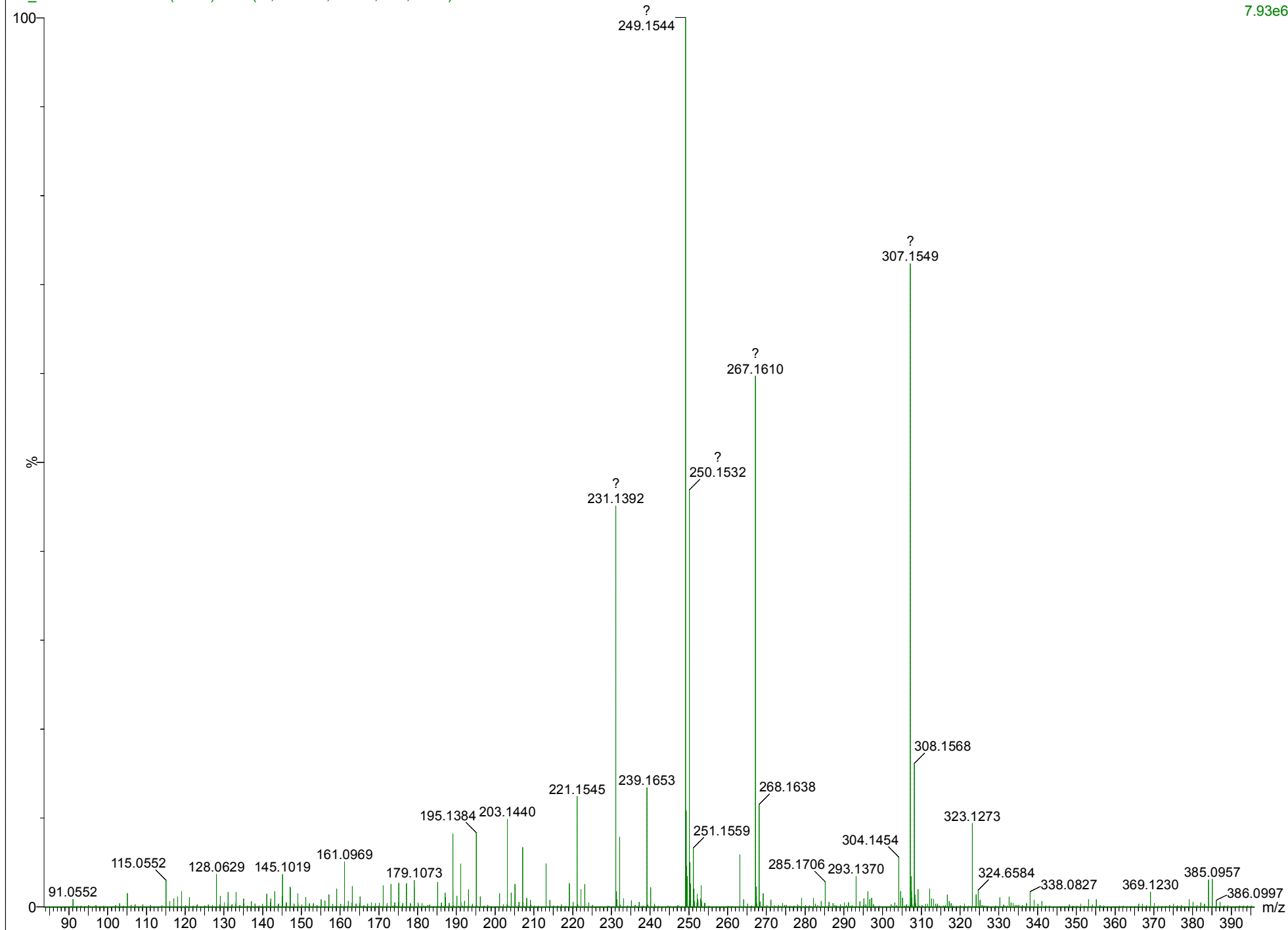

Supplement: Supplementary file 1 [file DataSheet1.ZIP › Supplementary Materials/Figure S20.HR-ESI-MS of Metabolite 6.pdf]

CARBON\_01  
VNS-600 CARBON M14 IN cdcl3 Sep 25 2020

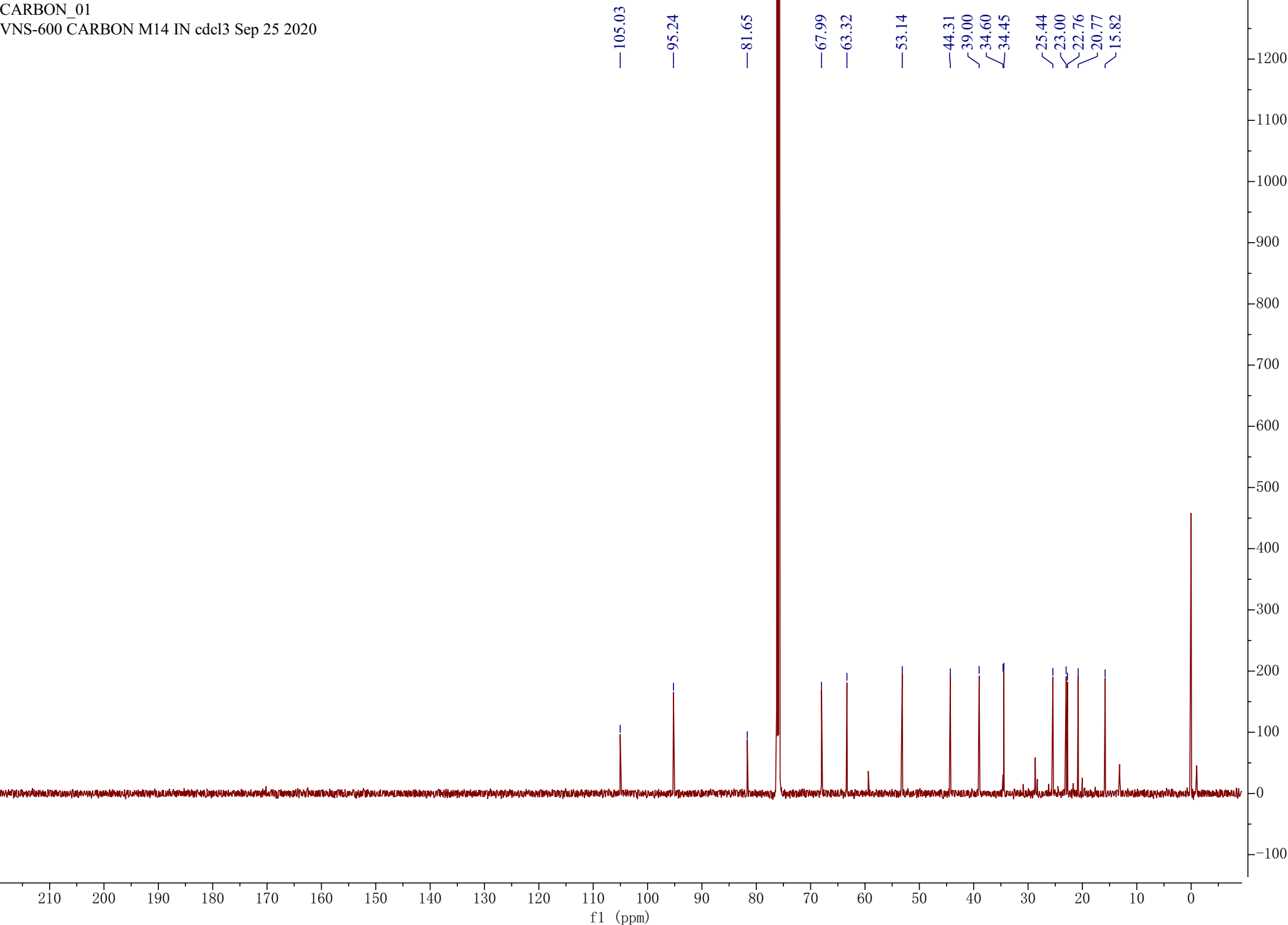

Supplement: Supplementary file 1 [file DataSheet1.ZIP › Supplementary Materials/Figure S21.C-NMR of Metabolite 7.pdf]

PROTON\_01  
VNS-600 PROTON M14 IN cdcl3 Sep 25 2020

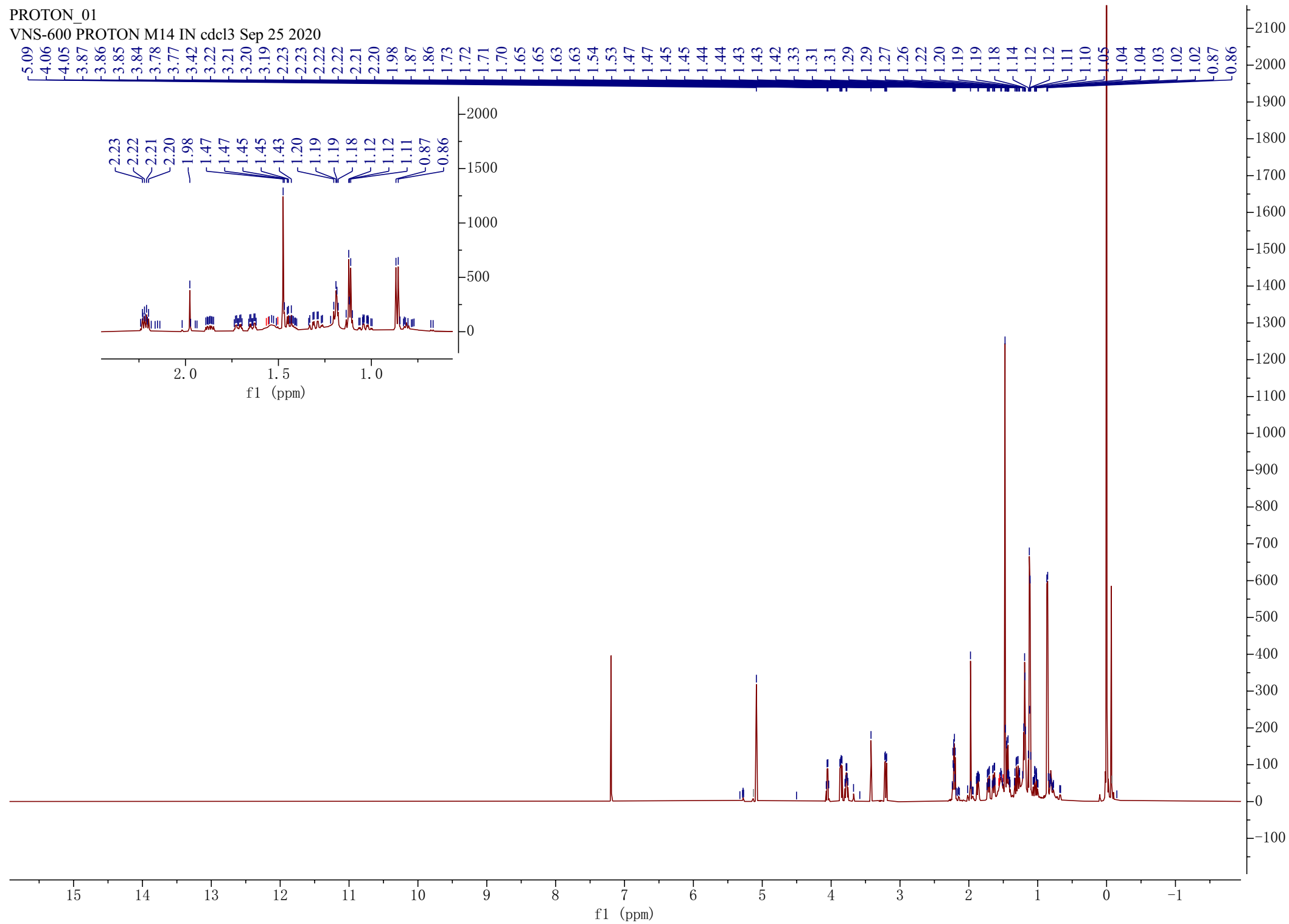

Supplement: Supplementary file 1 [file DataSheet1.ZIP › Supplementary Materials/Figure S22.H-NMR of Metabolite 7.pdf]

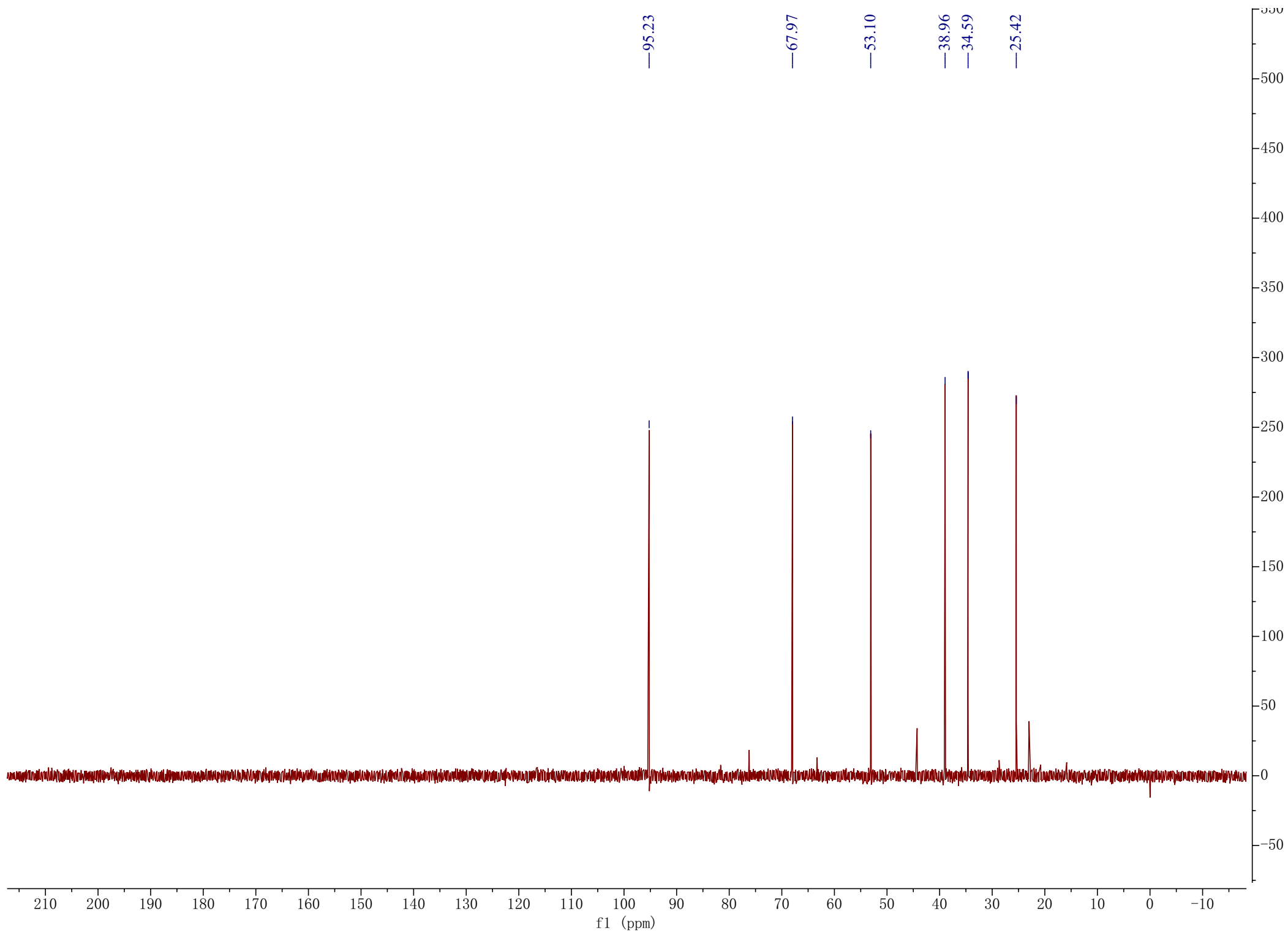

Supplement: Supplementary file 1 [file DataSheet1.ZIP › Supplementary Materials/Figure S23.DEPT(90) of Metabolite 7.pdf]

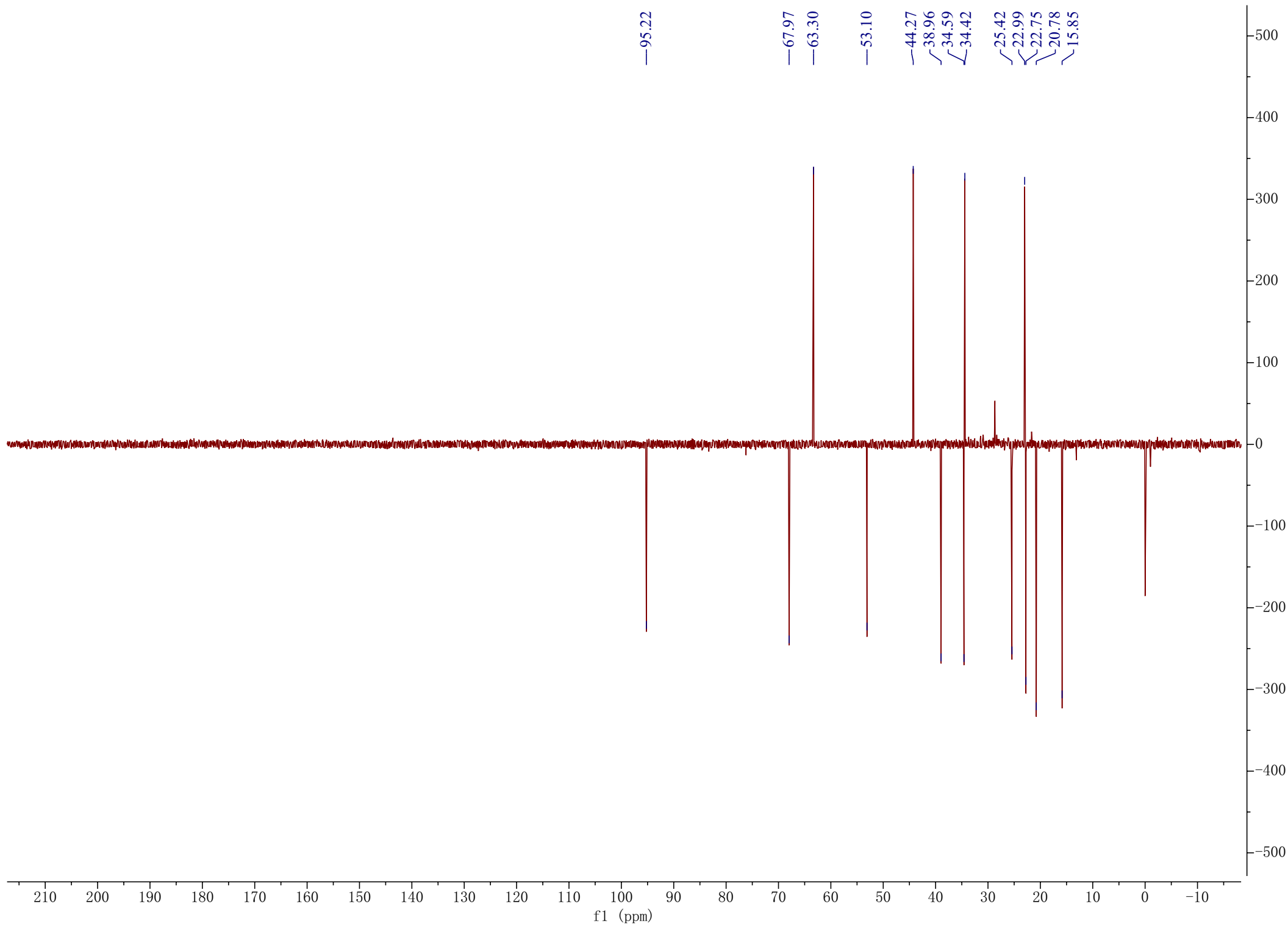

Supplement: Supplementary file 1 [file DataSheet1.ZIP › Supplementary Materials/Figure S24.DEPT(135) of Metabolite 7.pdf]

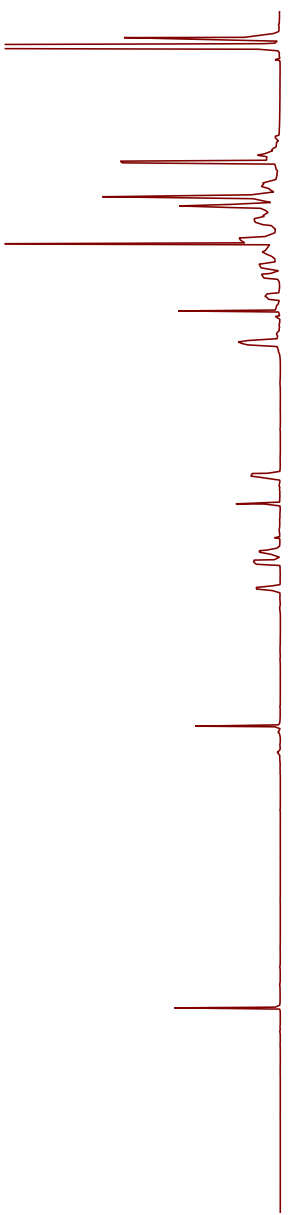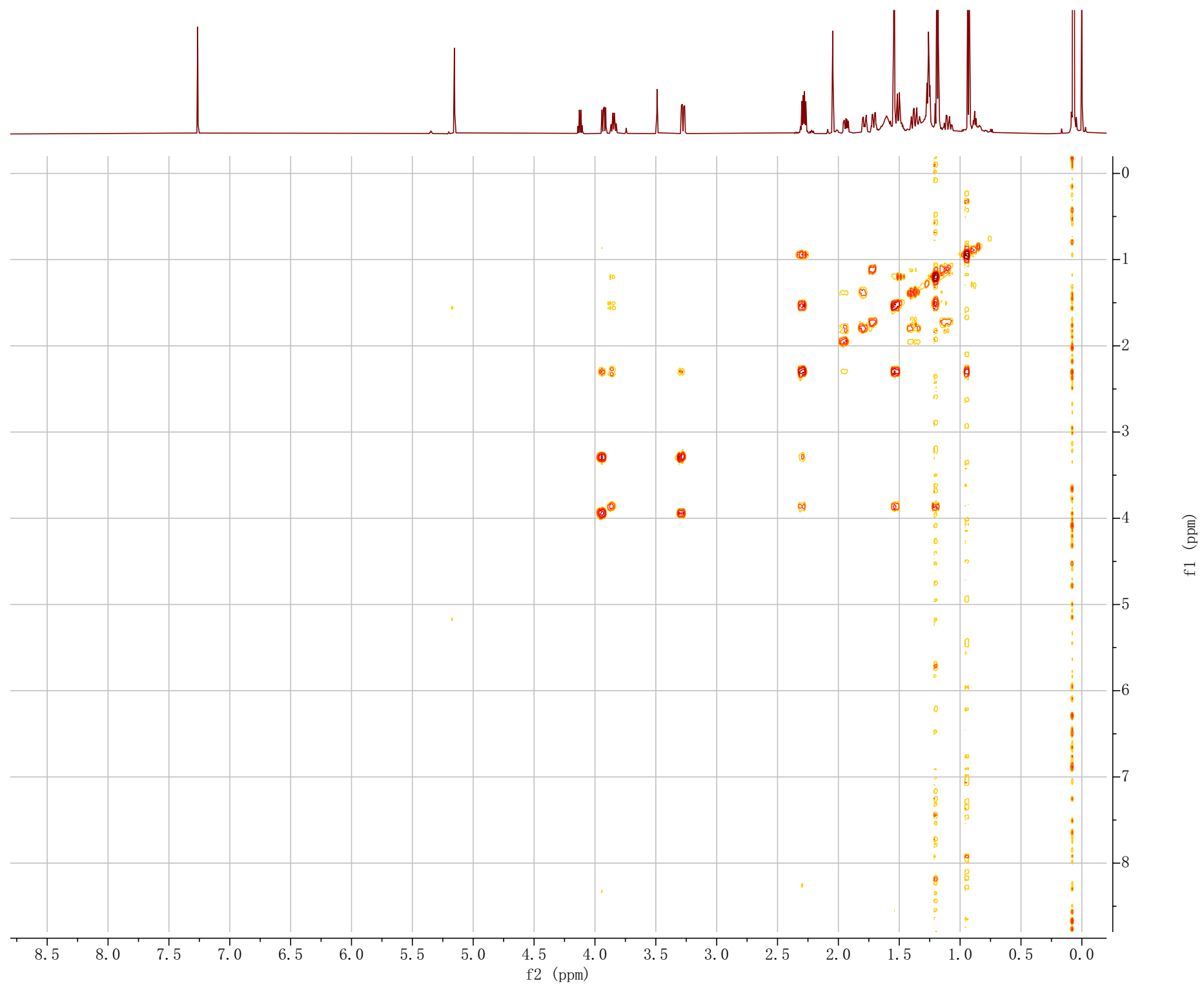

Supplement: Supplementary file 1 [file DataSheet1.ZIP › Supplementary Materials/Figure S25.H-H COSY of Metabolite 7.pdf]

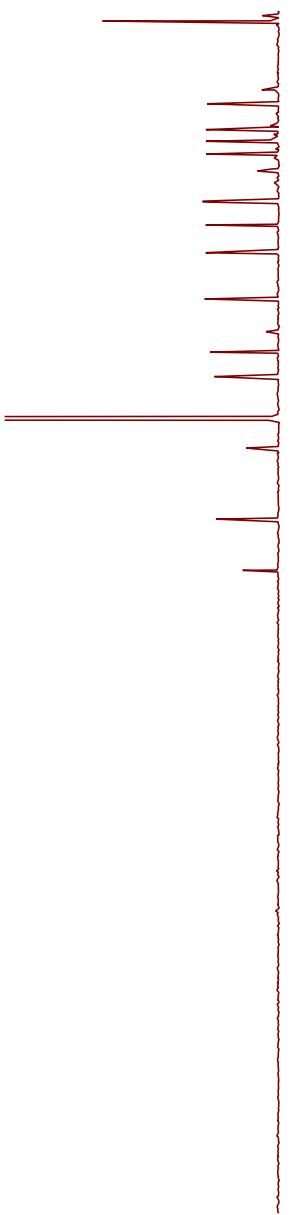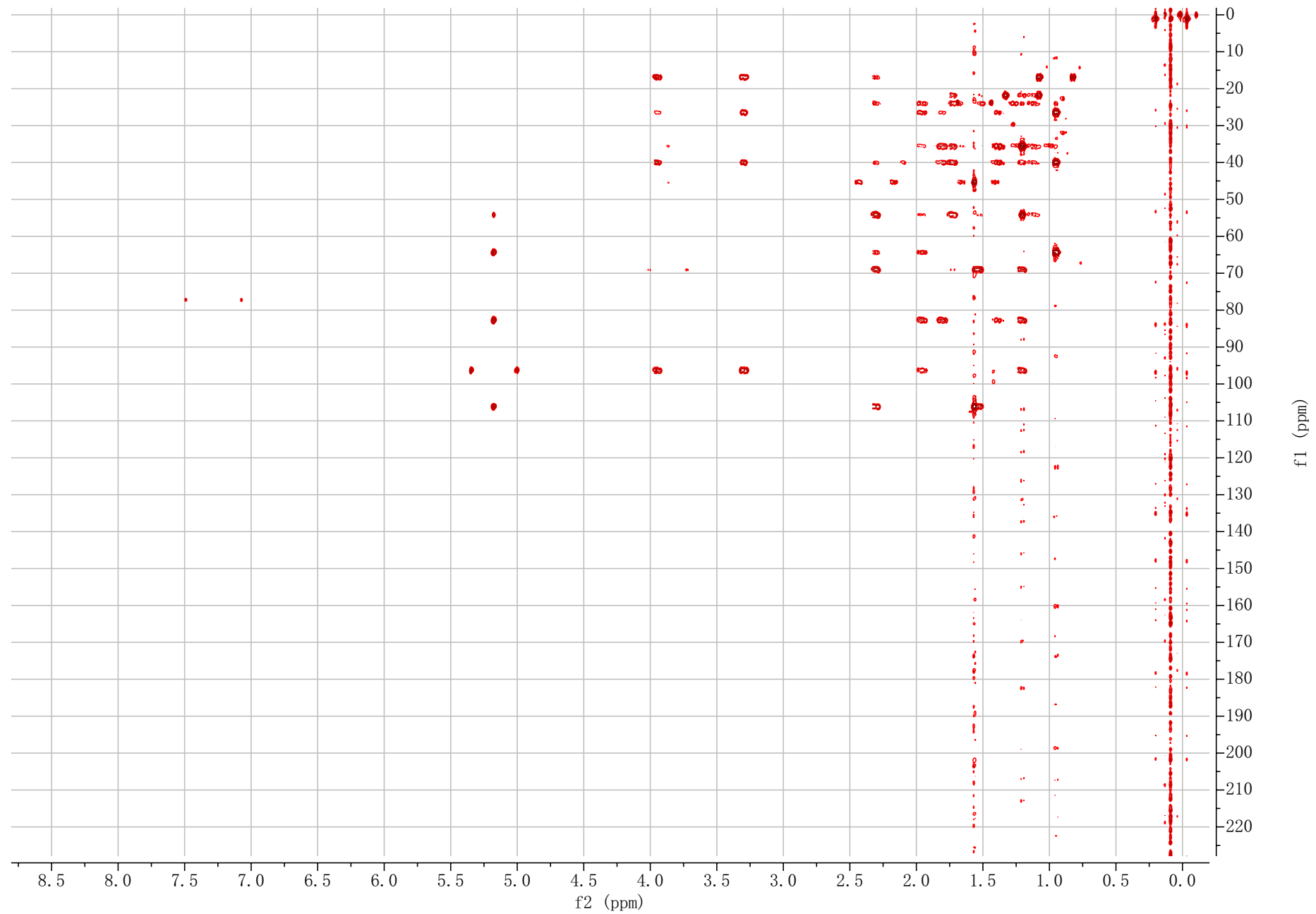

Supplement: Supplementary file 1 [file DataSheet1.ZIP › Supplementary Materials/Figure S26.HMBC of Metabolite 7.pdf]

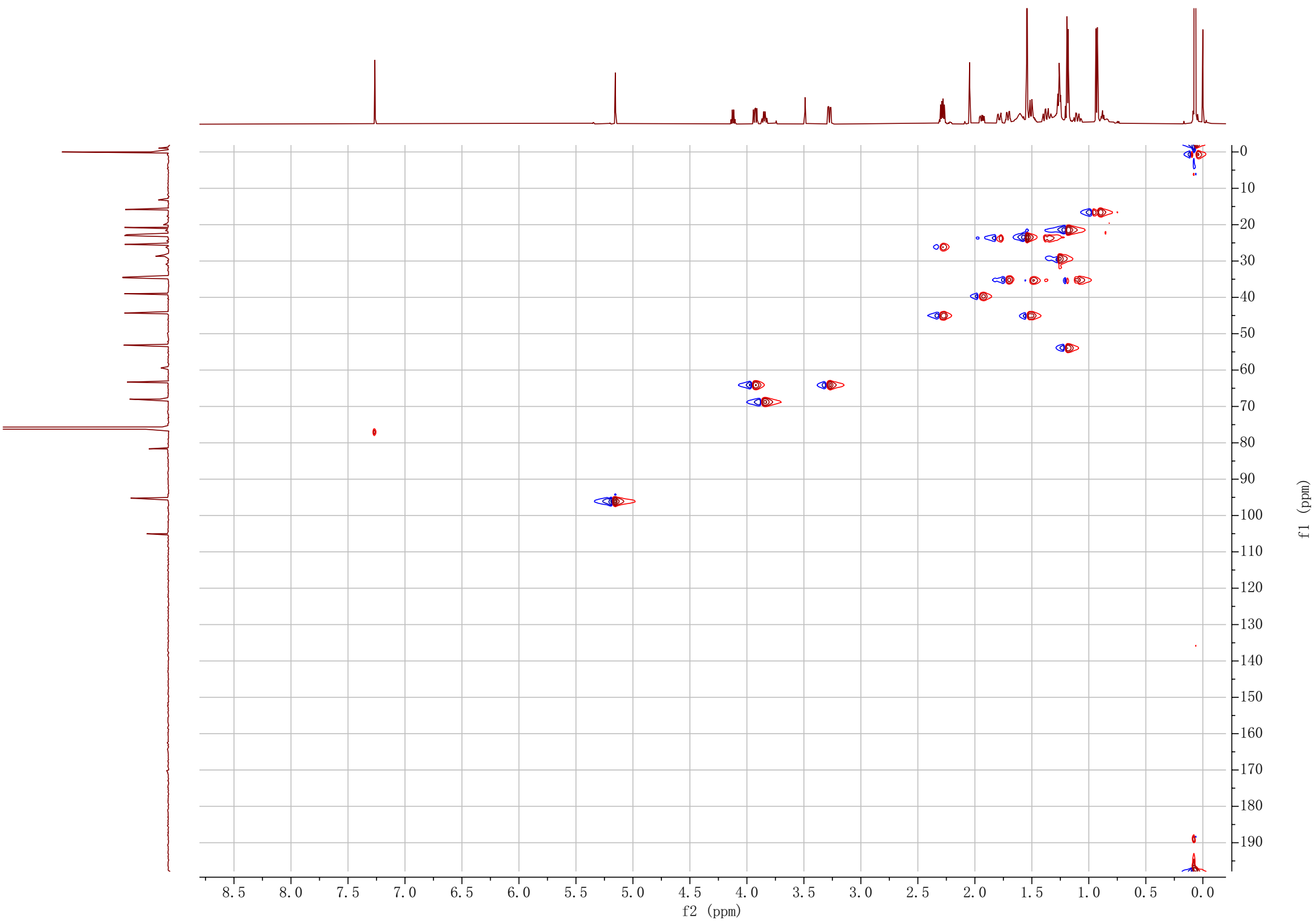

Supplement: Supplementary file 1 [file DataSheet1.ZIP › Supplementary Materials/Figure S27.HSQC of Metabolite 7.pdf]

28

BY\_20210130M14A\_P\_2 694 (5.174) AM2 (Ar,22000.0,556.28,0.00,LS 10)

1: TOF MS ES+  
2.01e5

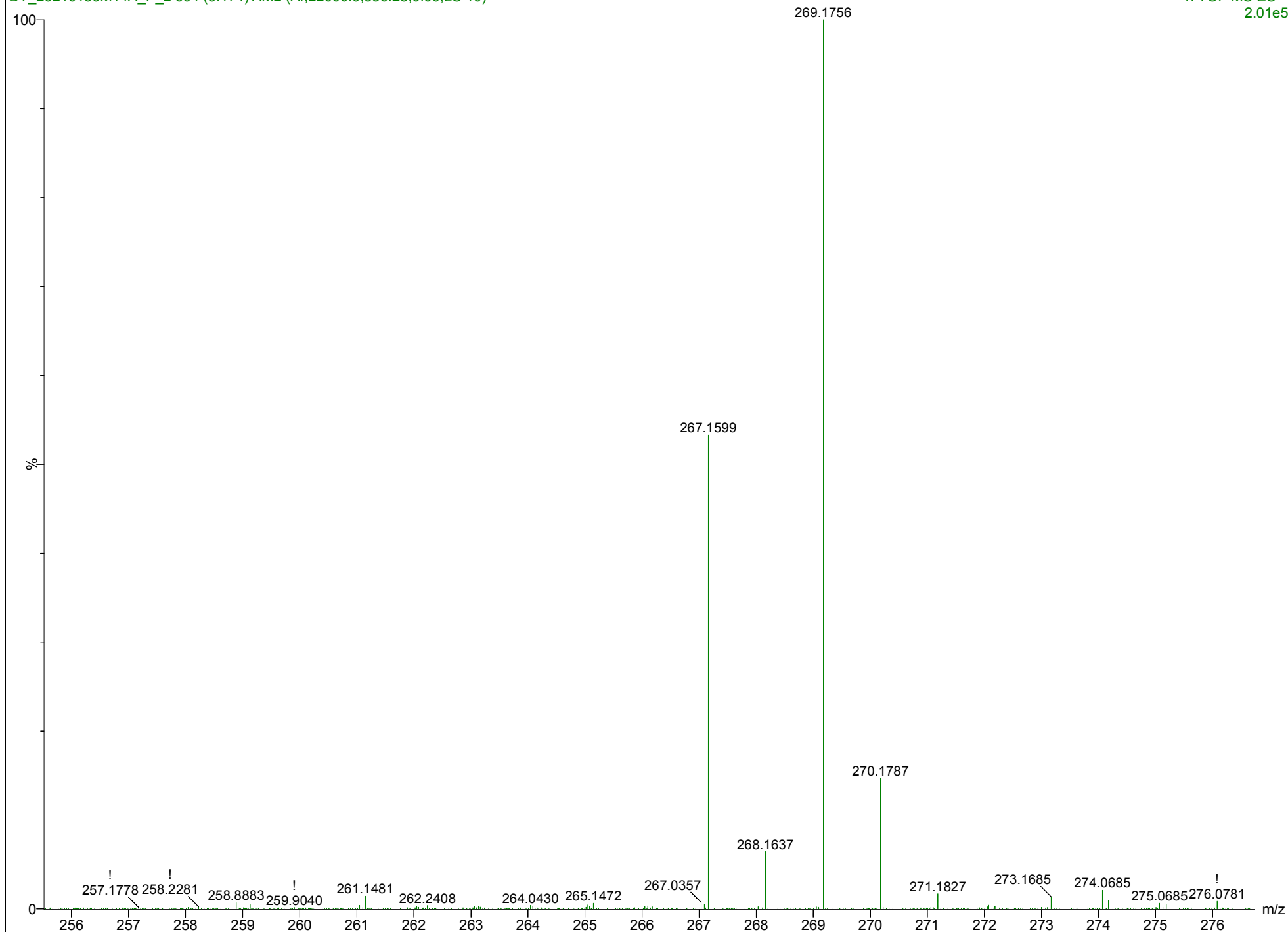

Supplement: Supplementary file 1 [file DataSheet1.ZIP › Supplementary Materials/Figure S28.HR-ESI-MS of Metabolite 7.pdf]

M20-MT3.2.fid  
Bruker AVIII HD 600  
C13 CDCl3 D:\DATA2021 22

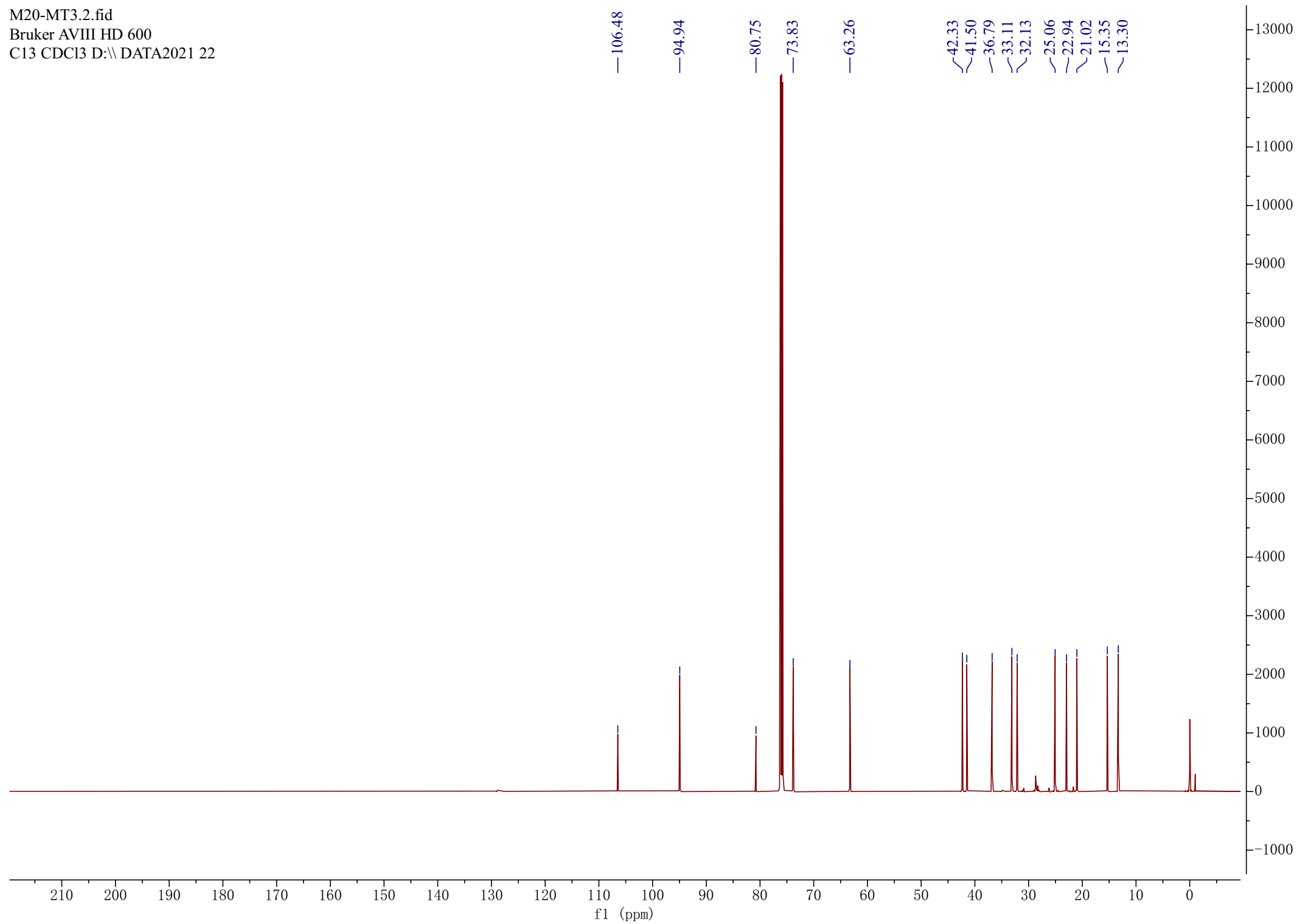

Supplement: Supplementary file 1 [file DataSheet1.ZIP › Supplementary Materials/Figure S29.C-NMR of Metabolite 8.pdf]

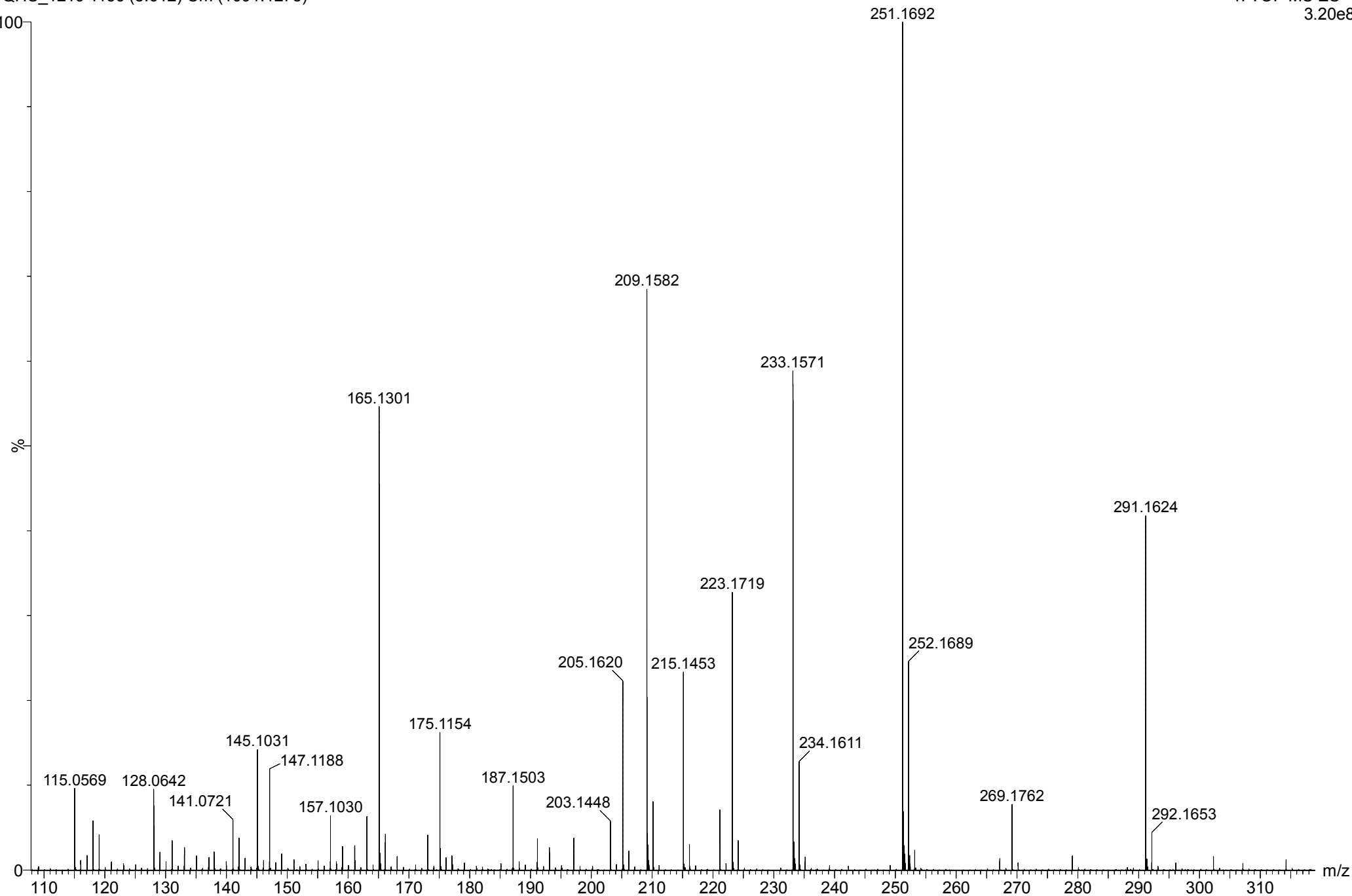

Supplement: Supplementary file 1 [file DataSheet1.ZIP › Supplementary Materials/Figure S3.HR-ESI-MS of Metabolite 2.pdf]

M20-MT3.1.fid  
Bruker AVIII HD 600  
PROTON CDC13 D:\DATA2021 22

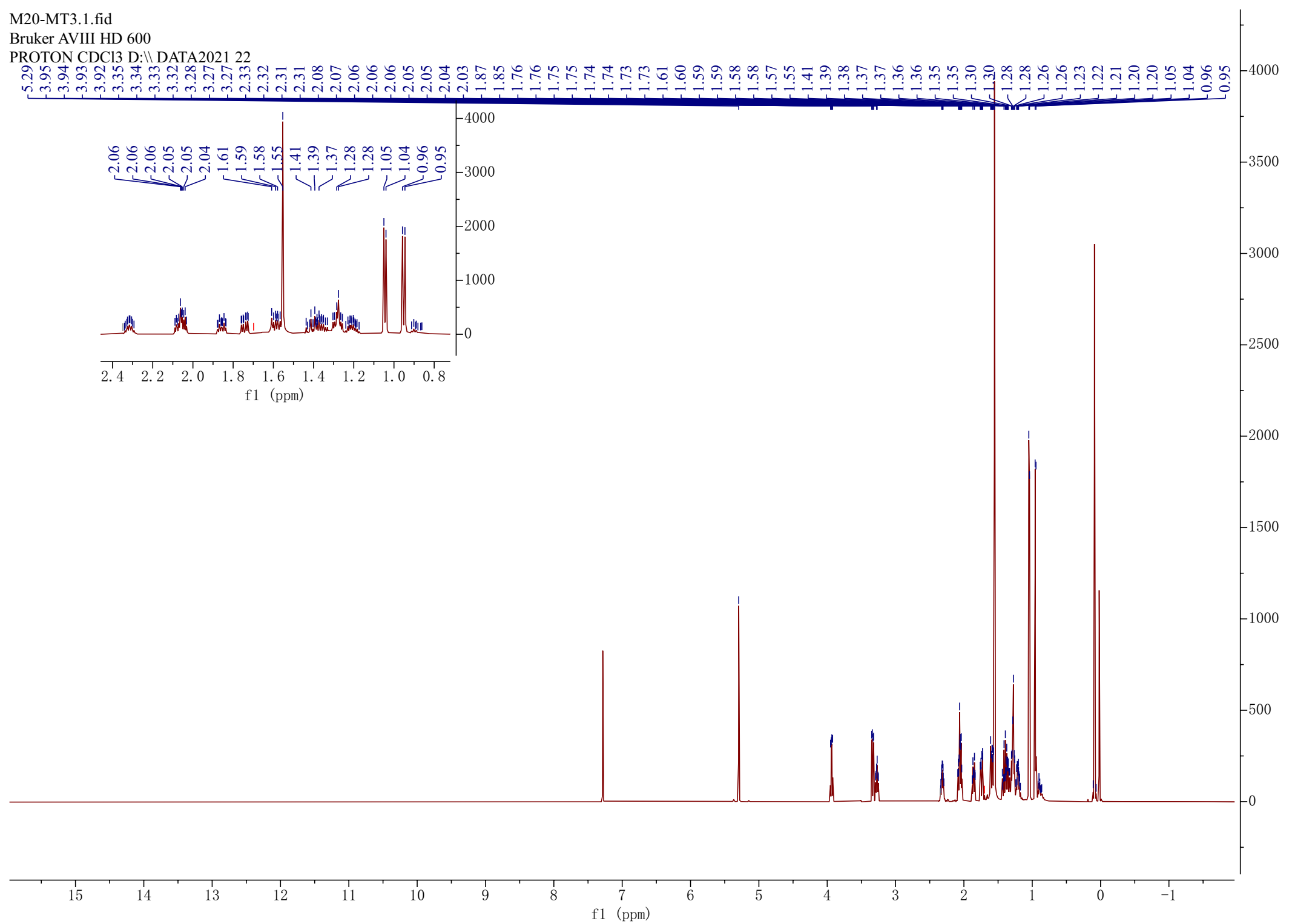

Supplement: Supplementary file 1 [file DataSheet1.ZIP › Supplementary Materials/Figure S30.H-NMR of Metabolite 8.pdf]

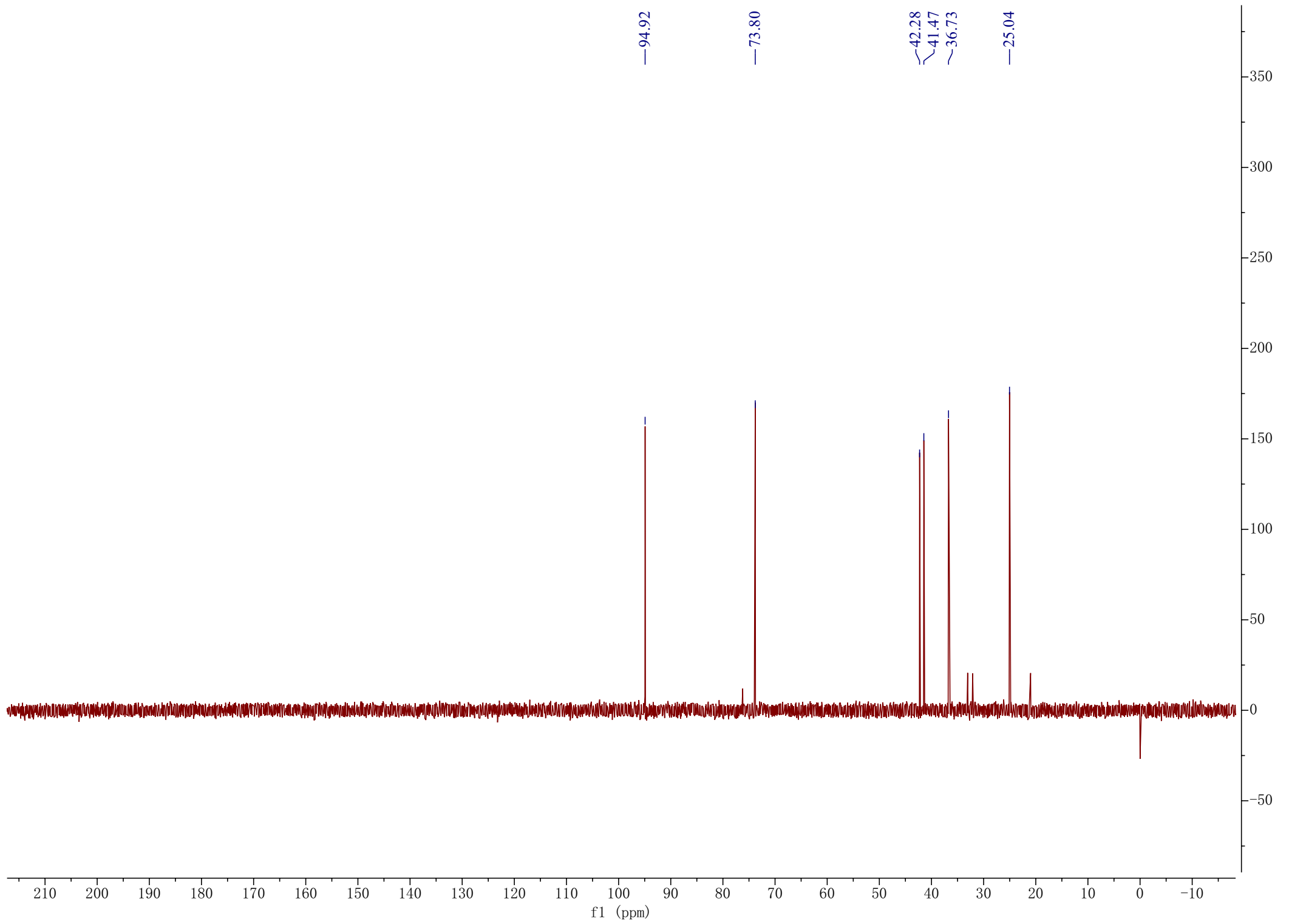

Supplement: Supplementary file 1 [file DataSheet1.ZIP › Supplementary Materials/Figure S31.DEPT(90) of Metabolite 8.pdf]

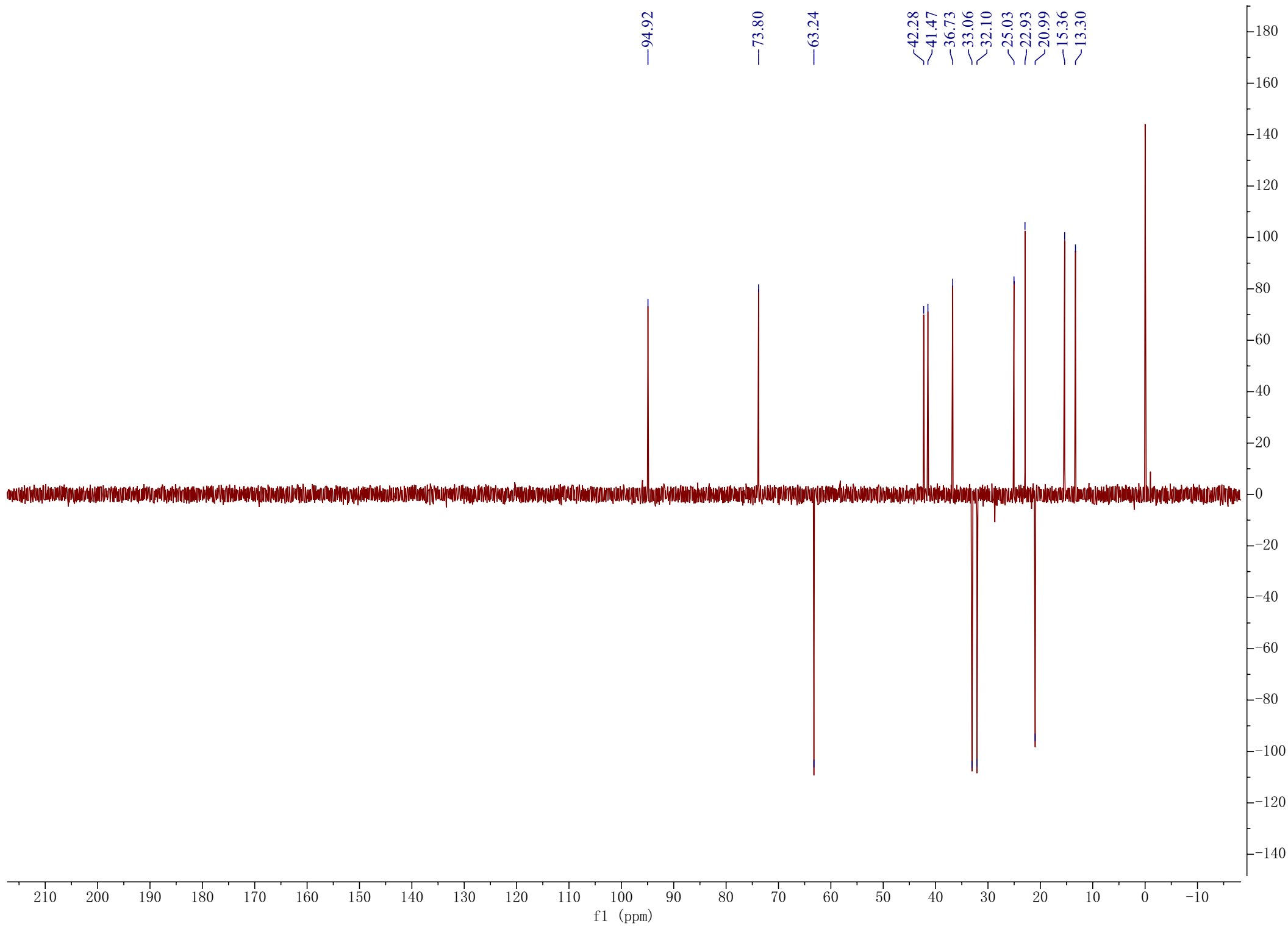

Supplement: Supplementary file 1 [file DataSheet1.ZIP › Supplementary Materials/Figure S32.DEPT(135) of Metabolite 8.pdf]

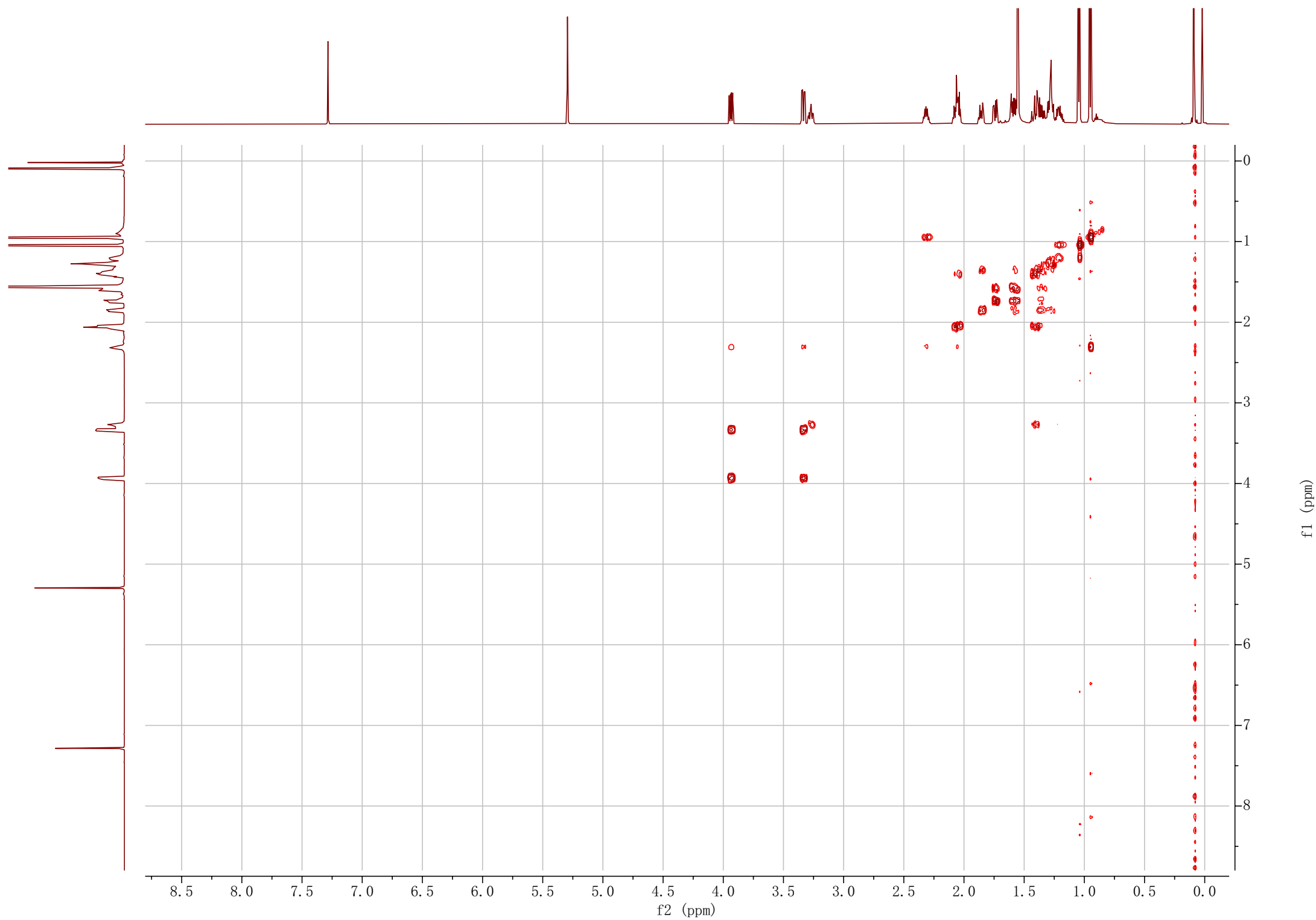

Supplement: Supplementary file 1 [file DataSheet1.ZIP › Supplementary Materials/Figure S33.H-H COSY of Metabolite 8.pdf]

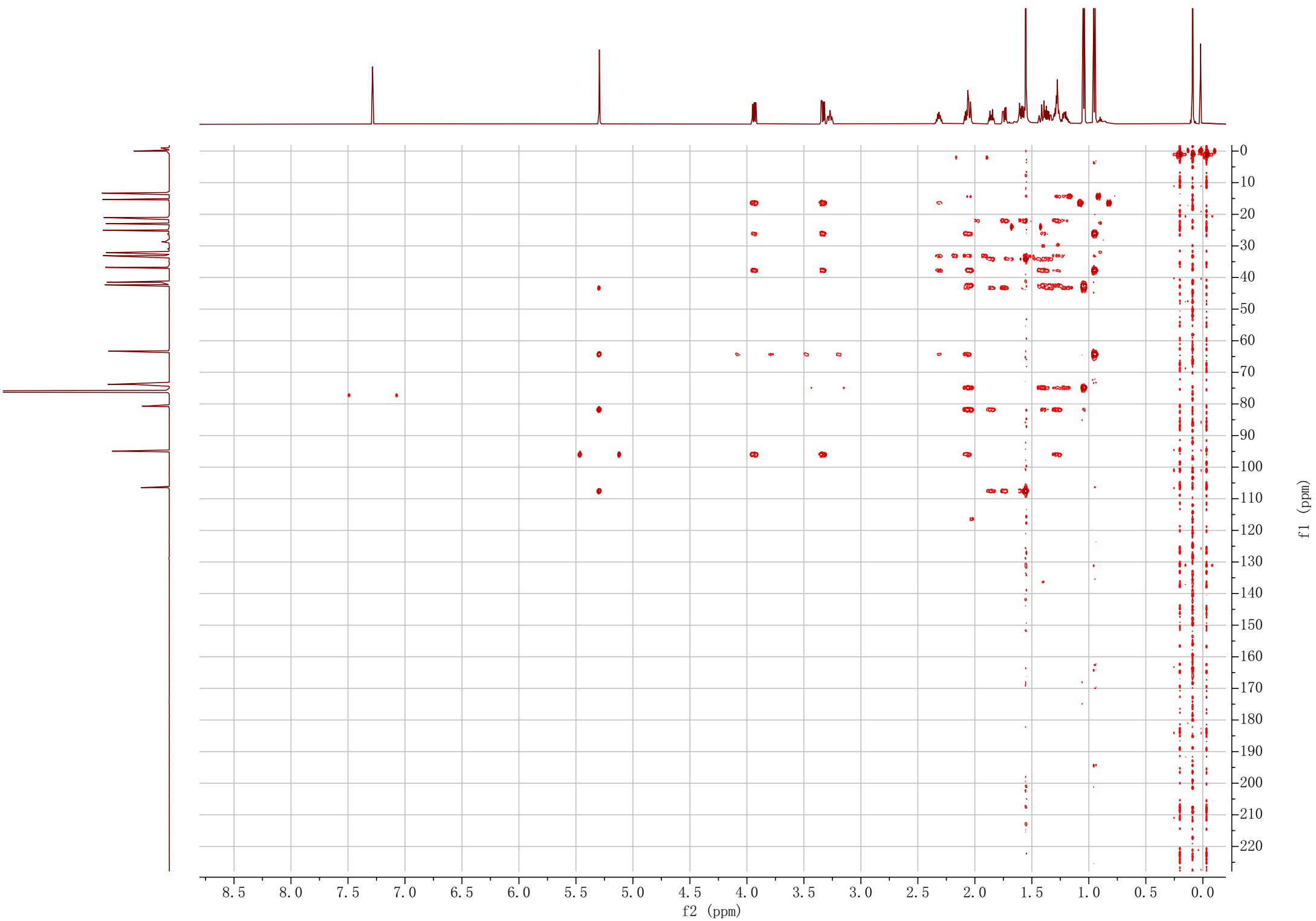

Supplement: Supplementary file 1 [file DataSheet1.ZIP › Supplementary Materials/Figure S34.HMBC of Metabolite 8.pdf]

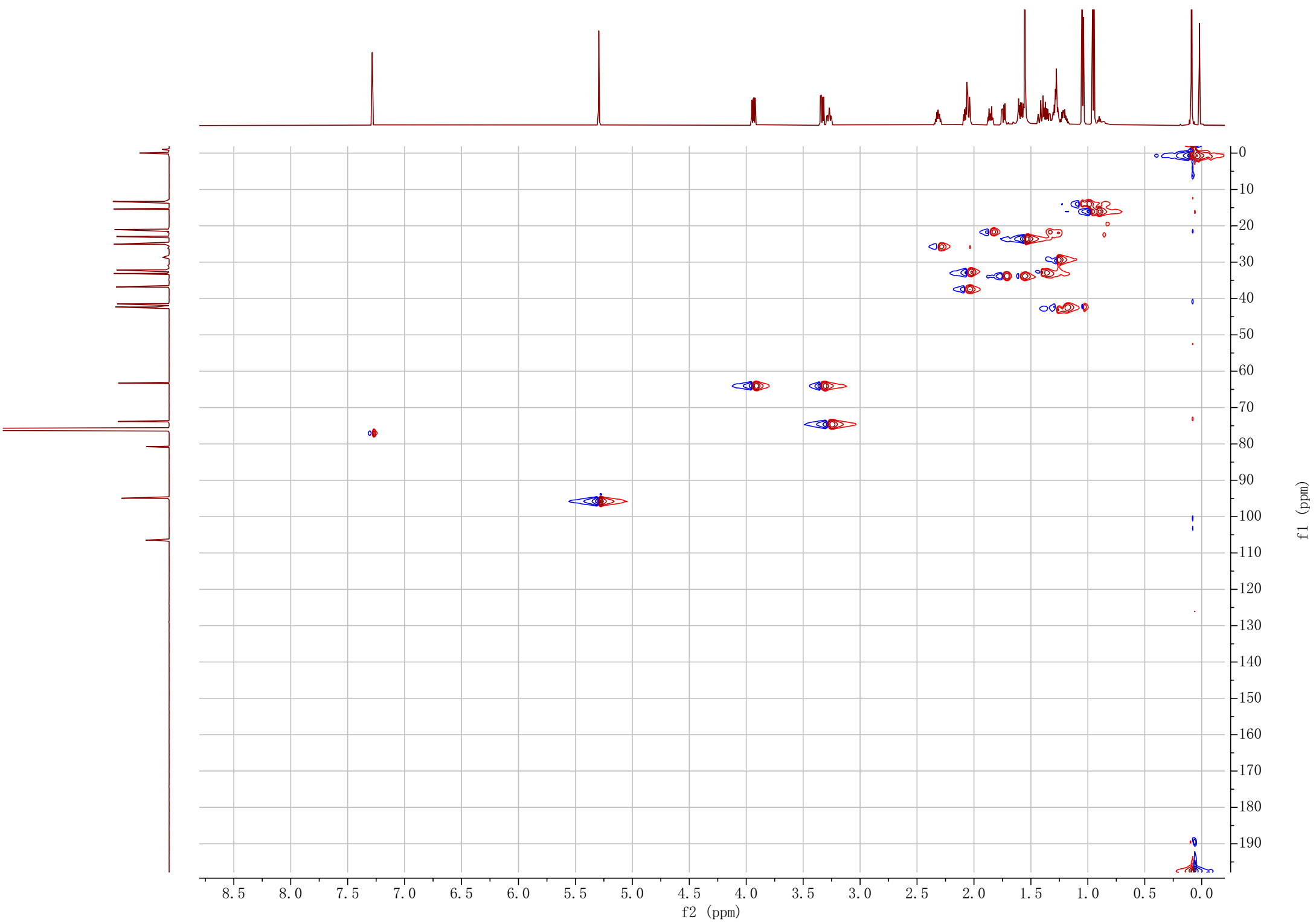

Supplement: Supplementary file 1 [file DataSheet1.ZIP › Supplementary Materials/Figure S35.HSQC of Metabolite 8.pdf]

11

BY\_20200107M20P 594 (4.433) AM2 (Ar,22000.0,556.28,0.00,LS 10)

1: TOF MS ES+  
1.09e5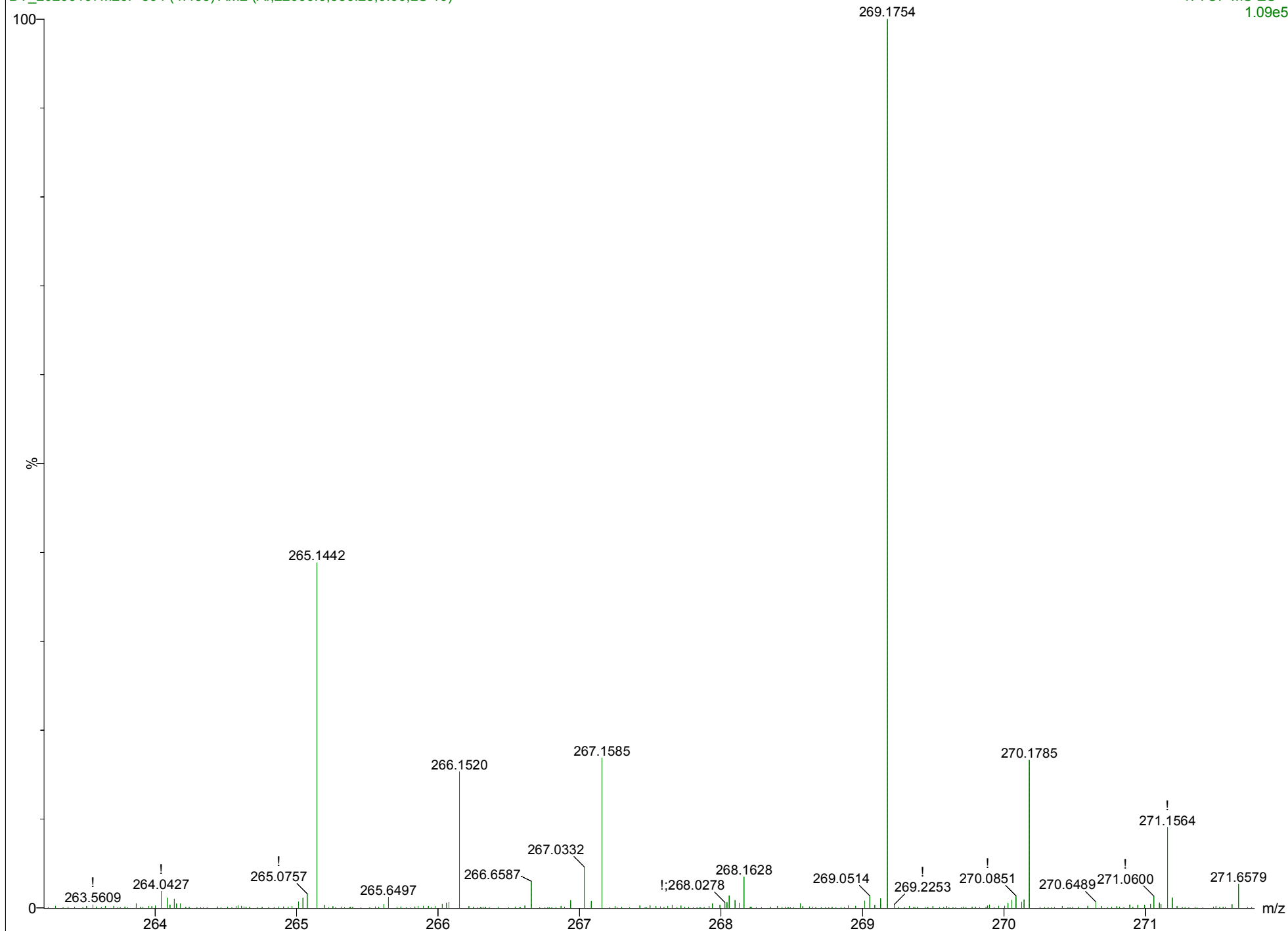

Supplement: Supplementary file 1 [file DataSheet1.ZIP › Supplementary Materials/Figure S36.HR-ESI-MS of Metabolite 8.pdf]

M21-MT3.2.fid  
C13CPD MeOD D:\ nmrsu 30

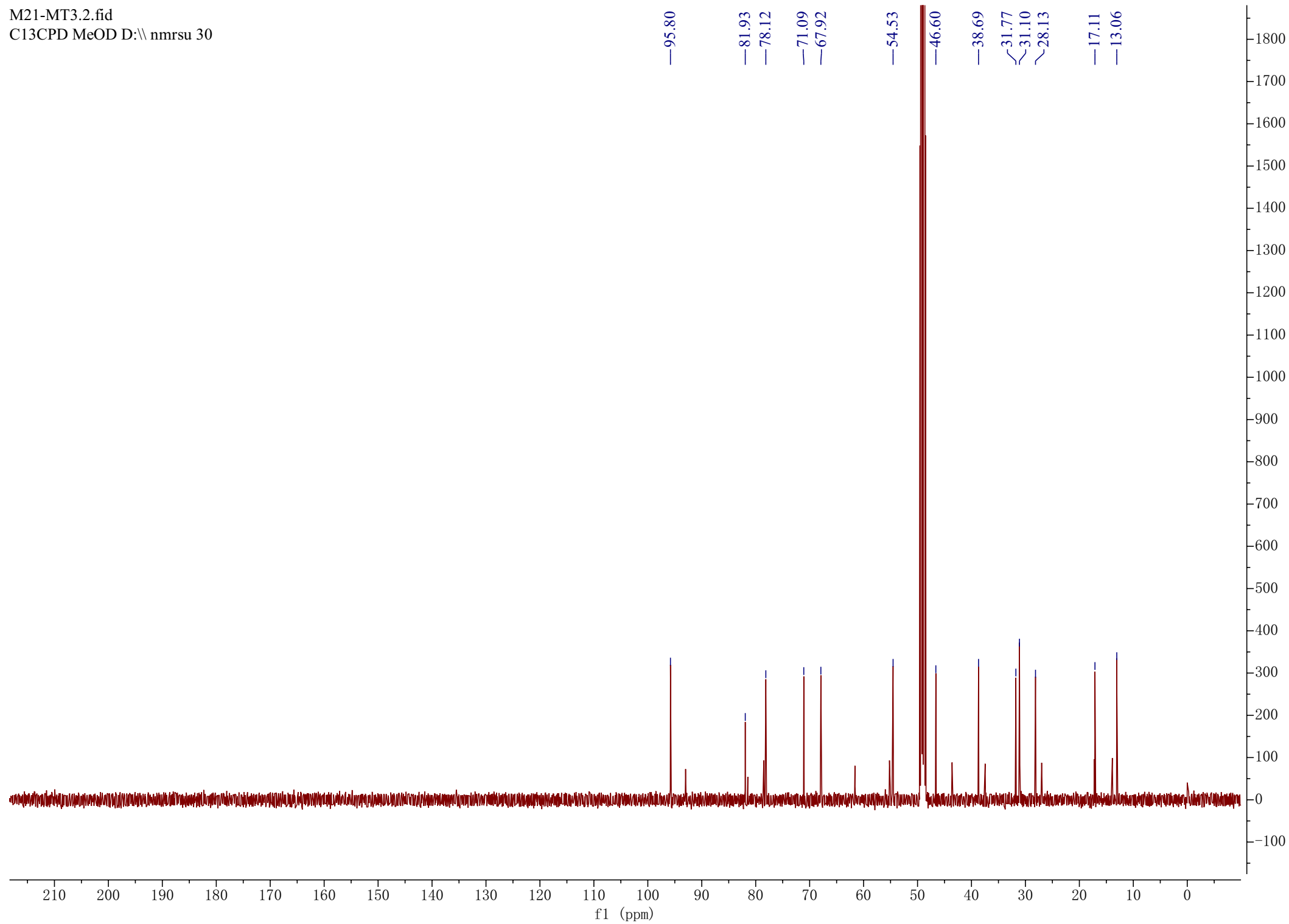

Supplement: Supplementary file 1 [file DataSheet1.ZIP › Supplementary Materials/Figure S37.C-NMR of Metabolite 9.pdf]

M21-MT3.1.fid  
PROTON MeOD D:\\ nmrsu 30

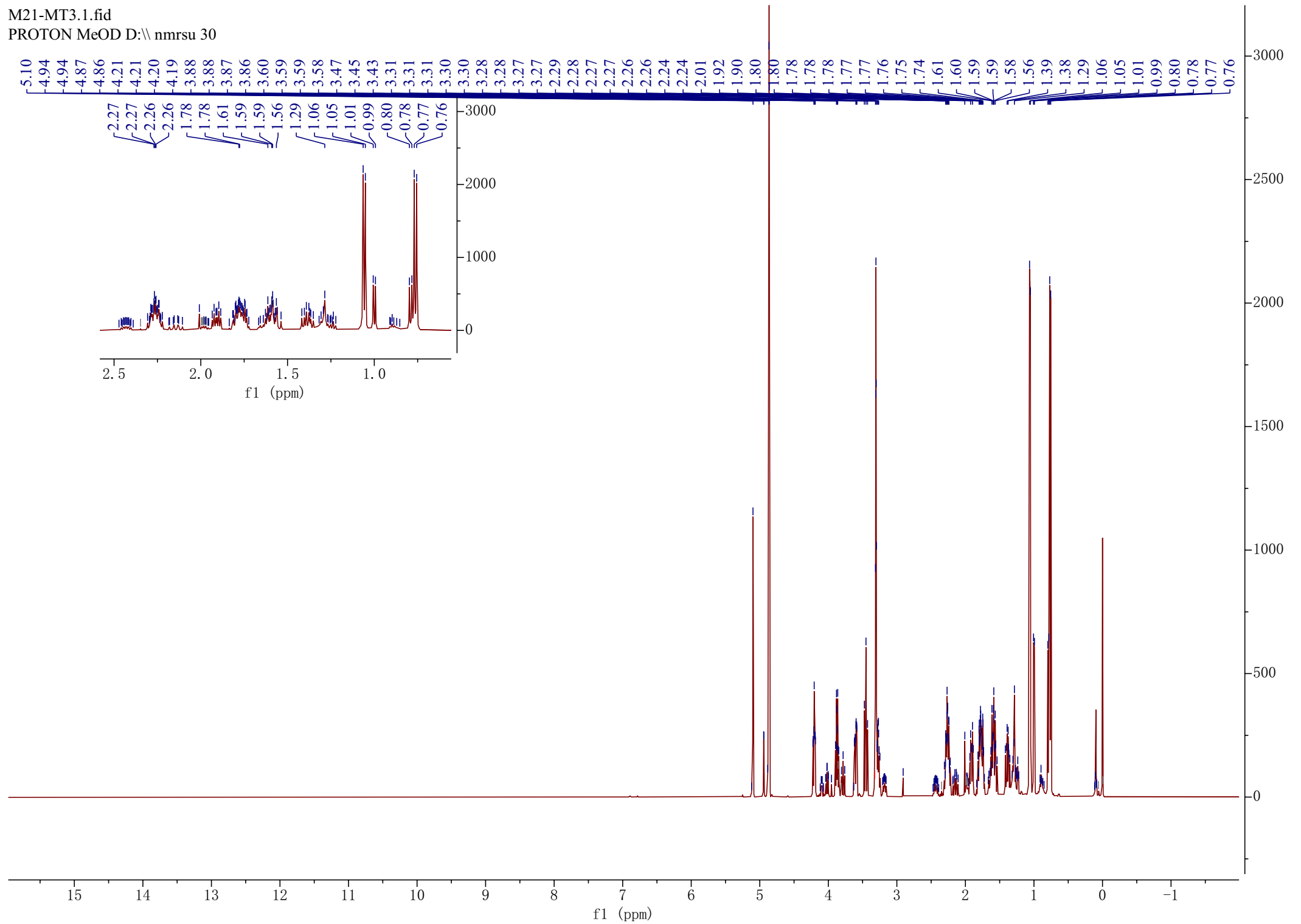

Supplement: Supplementary file 1 [file DataSheet1.ZIP › Supplementary Materials/Figure S38.H-NMR of Metabolite 9.pdf]

29

BY\_20210130M21A\_P\_2 248 (1.857) AM2 (Ar,22000.0,556.28,0.00,LS 10)

1: TOF MS ES+  
3.91e5

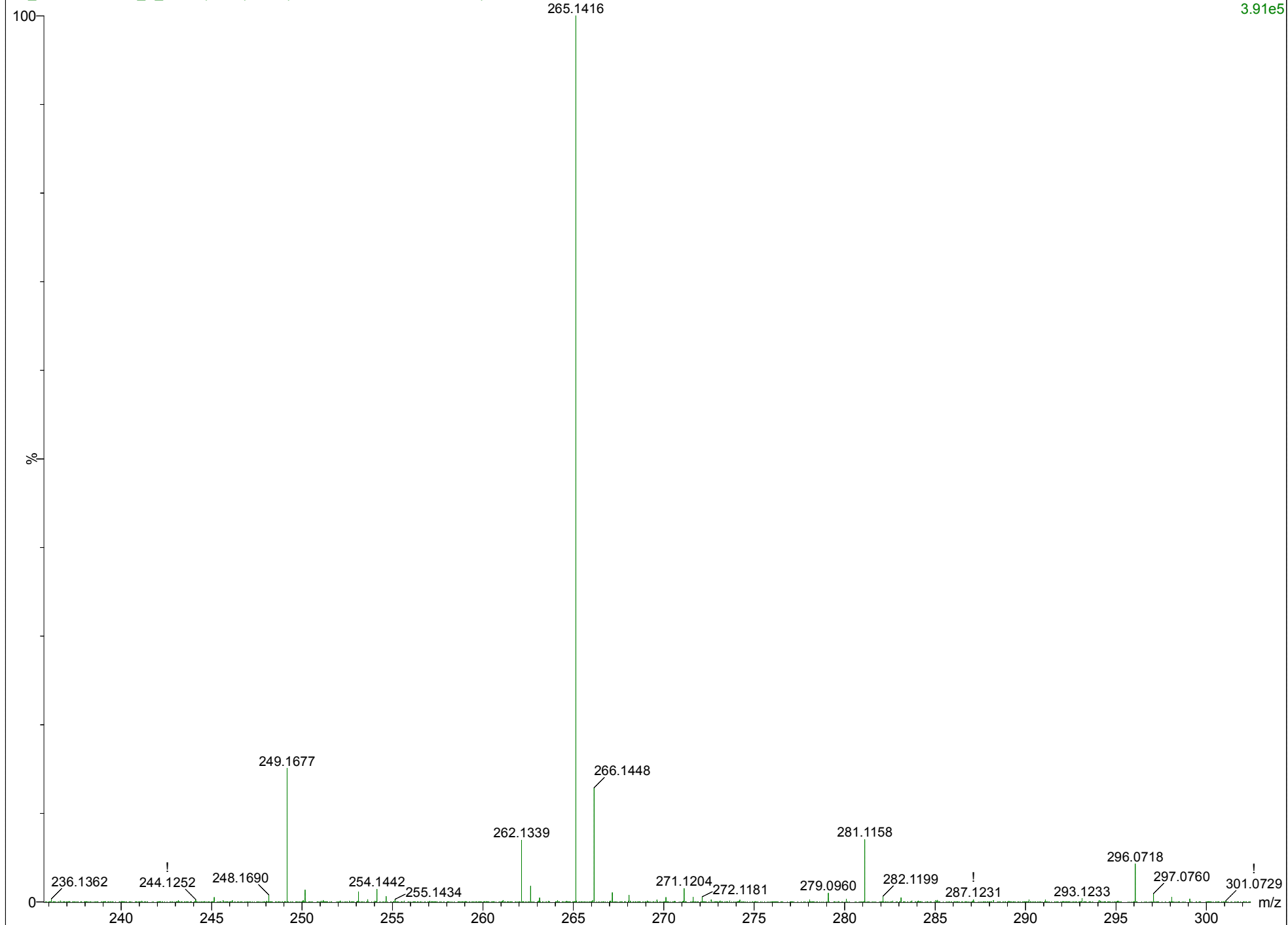

Supplement: Supplementary file 1 [file DataSheet1.ZIP › Supplementary Materials/Figure S39.HR-ESI-MS of Metabolite 9.pdf]

CARBON\_01  
VNS-600 CARBON Z1 IN dmso Jan 10 2019

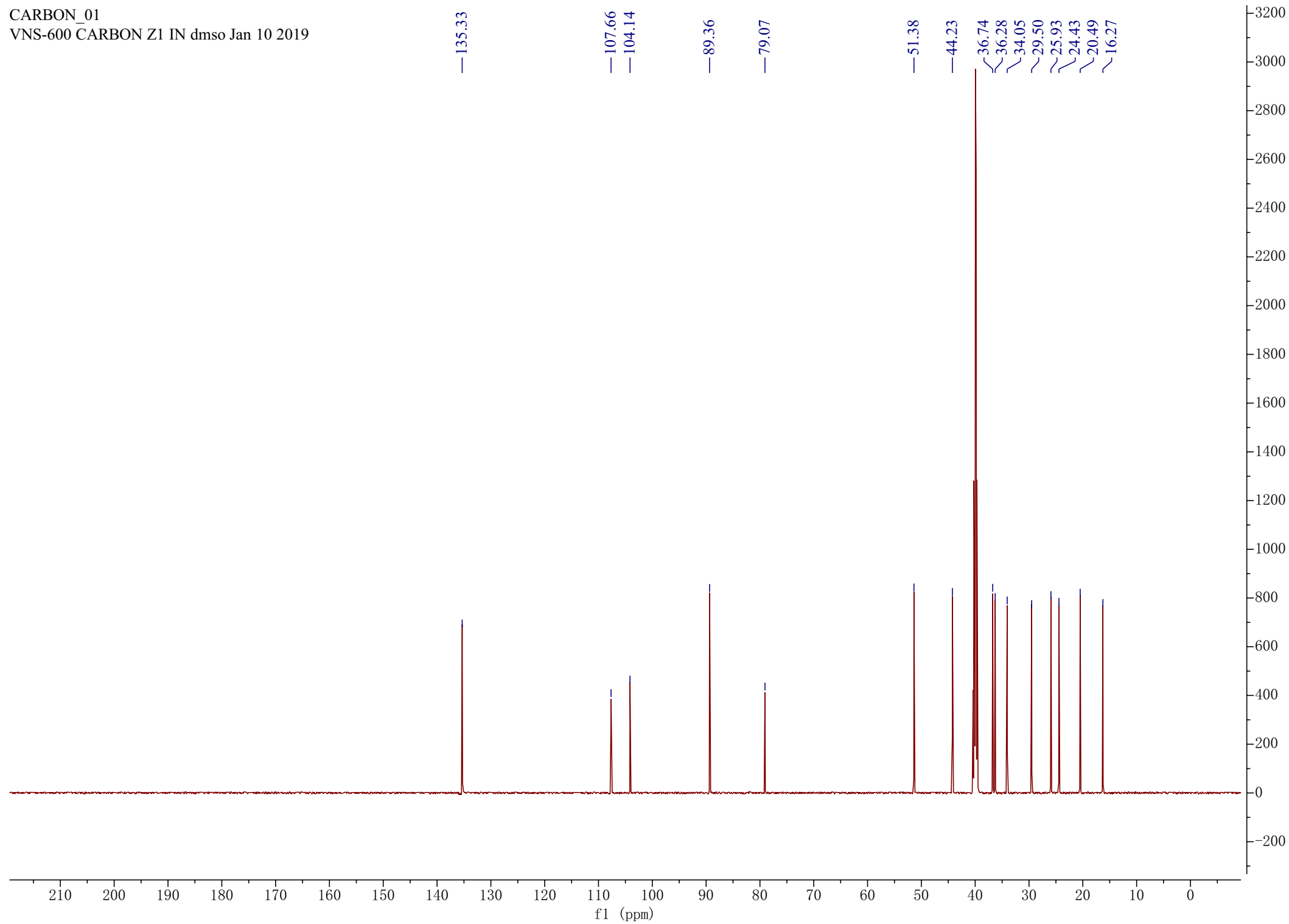

Supplement: Supplementary file 1 [file DataSheet1.ZIP › Supplementary Materials/Figure S4.C-NMR of Metabolite 3.pdf]

CARBON\_01  
VNS-600 CARBON 1110M23 IN cdcl3 Nov 12 2020

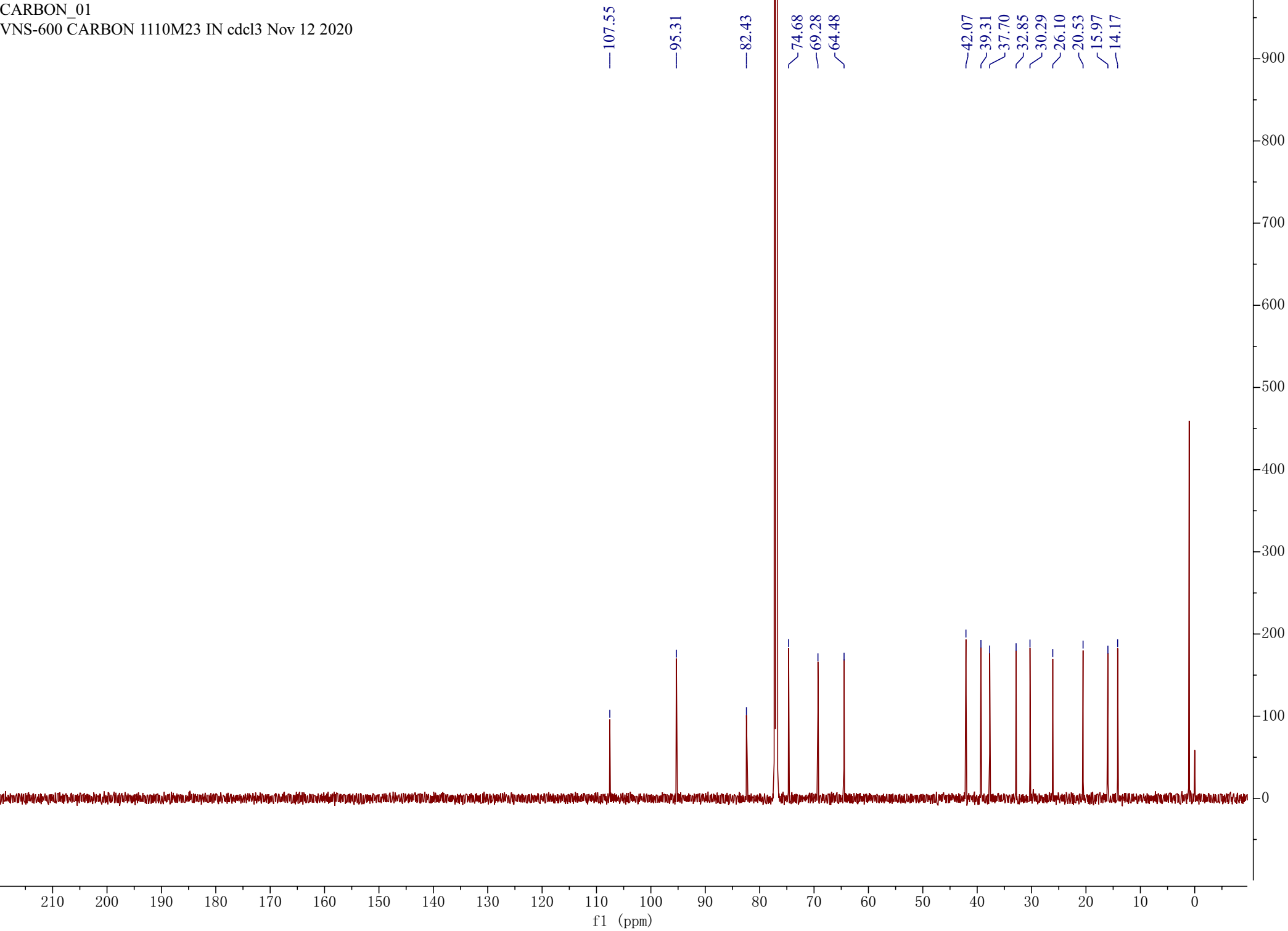

Supplement: Supplementary file 1 [file DataSheet1.ZIP › Supplementary Materials/Figure S40.C-NMR of Metabolite 10.pdf]

PROTON\_01  
VNS-600 PROTON LEUM23INcdel3 Nov 12 2020

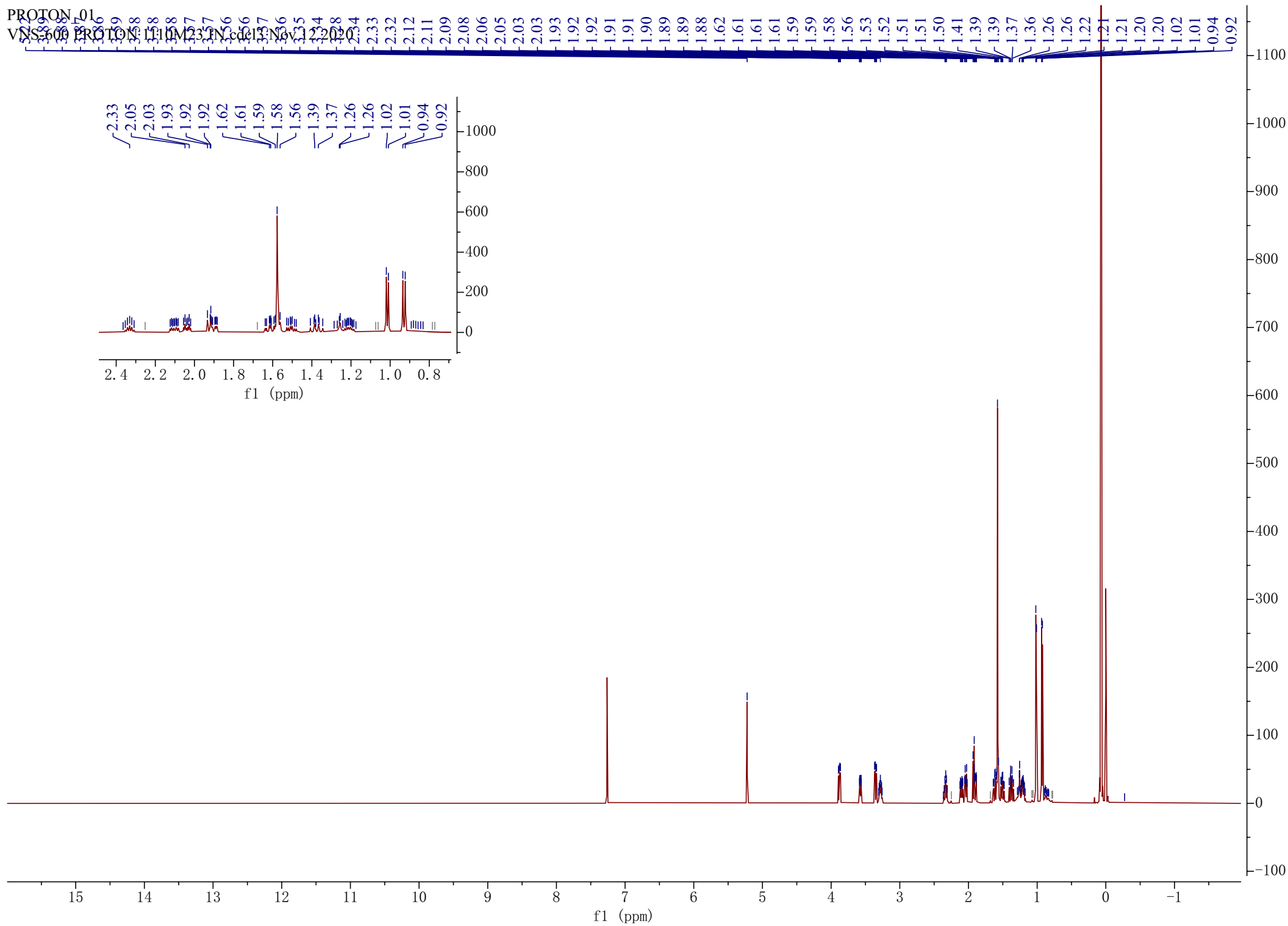

Supplement: Supplementary file 1 [file DataSheet1.ZIP › Supplementary Materials/Figure S41.H-NMR of Metabolite 10.pdf]

13

BY\_20200107M23P 273 (2.048) AM2 (Ar,22000.0,556.28,0.00,LS 10)

1: TOF MS ES+  
2.96e4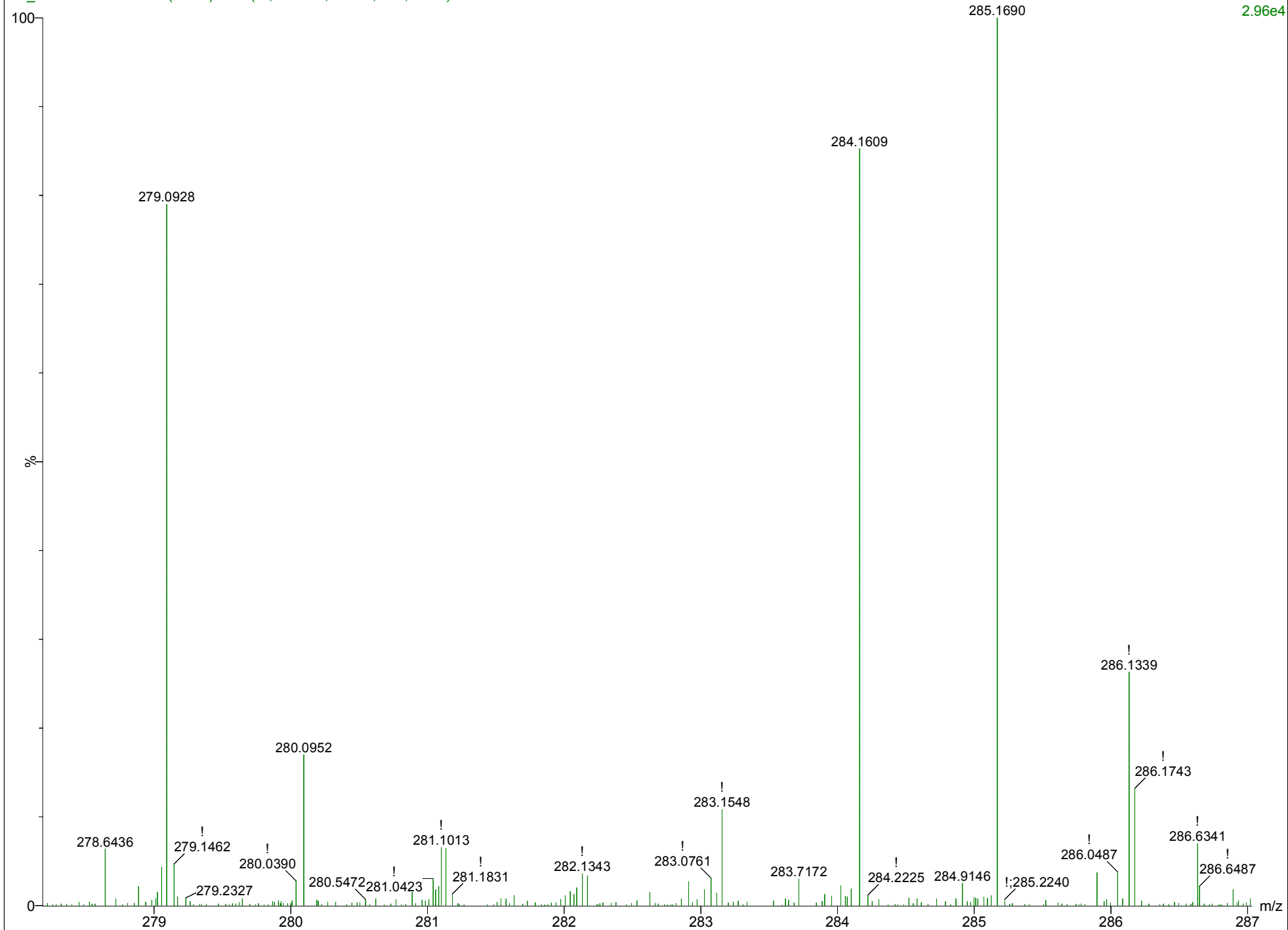

Supplement: Supplementary file 1 [file DataSheet1.ZIP › Supplementary Materials/Figure S42.HR-ESI-MS of Metabolite 10.pdf]

CARBON\_01  
VNS-600 CARBON MT4-M1 IN cdcl3 Sep 10 2019

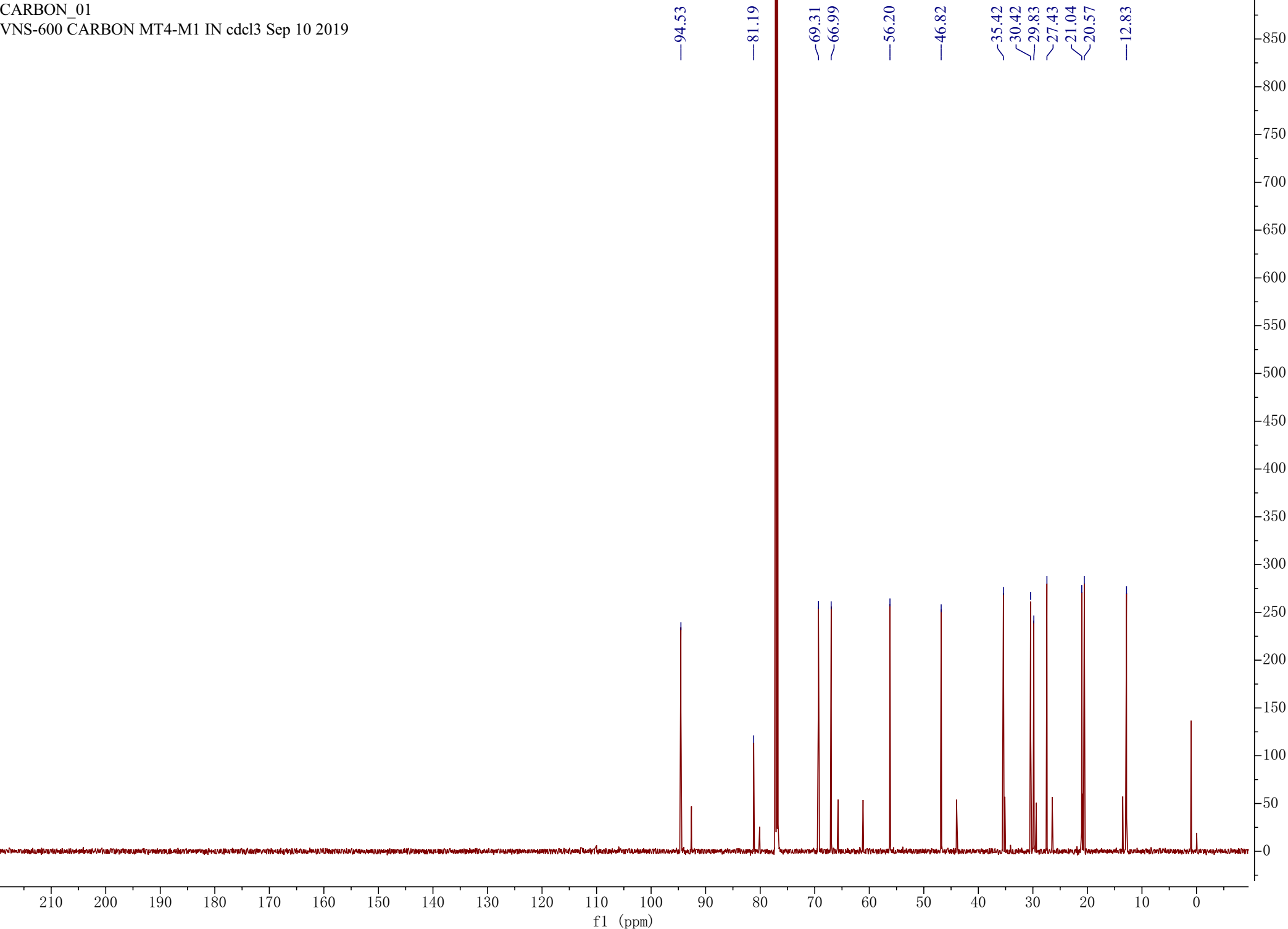

Supplement: Supplementary file 1 [file DataSheet1.ZIP › Supplementary Materials/Figure S43.C-NMR of Metabolite 11.pdf]

PROTON\_01  
VNS-600 PROTON MT4-M1 IN cdcl3 Sep 10 2019

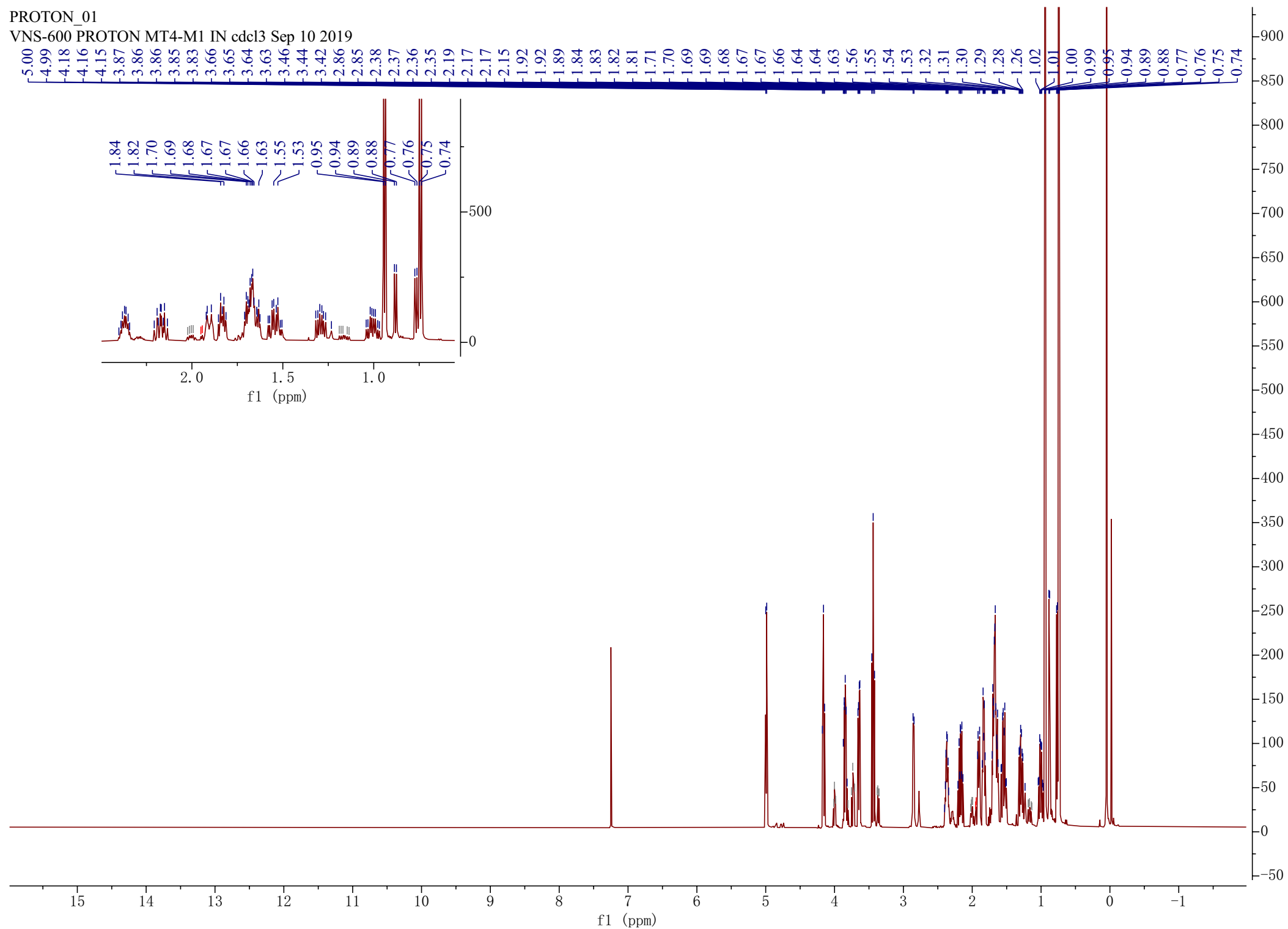

Supplement: Supplementary file 1 [file DataSheet1.ZIP › Supplementary Materials/Figure S44.H-NMR of Metabolite 11.pdf]

1

BY\_MT4\_M1 814 (6.070) AM2 (Ar,22000.0,556.28,0.00,LS 10)

1: TOF MS ES+  
7.25e6

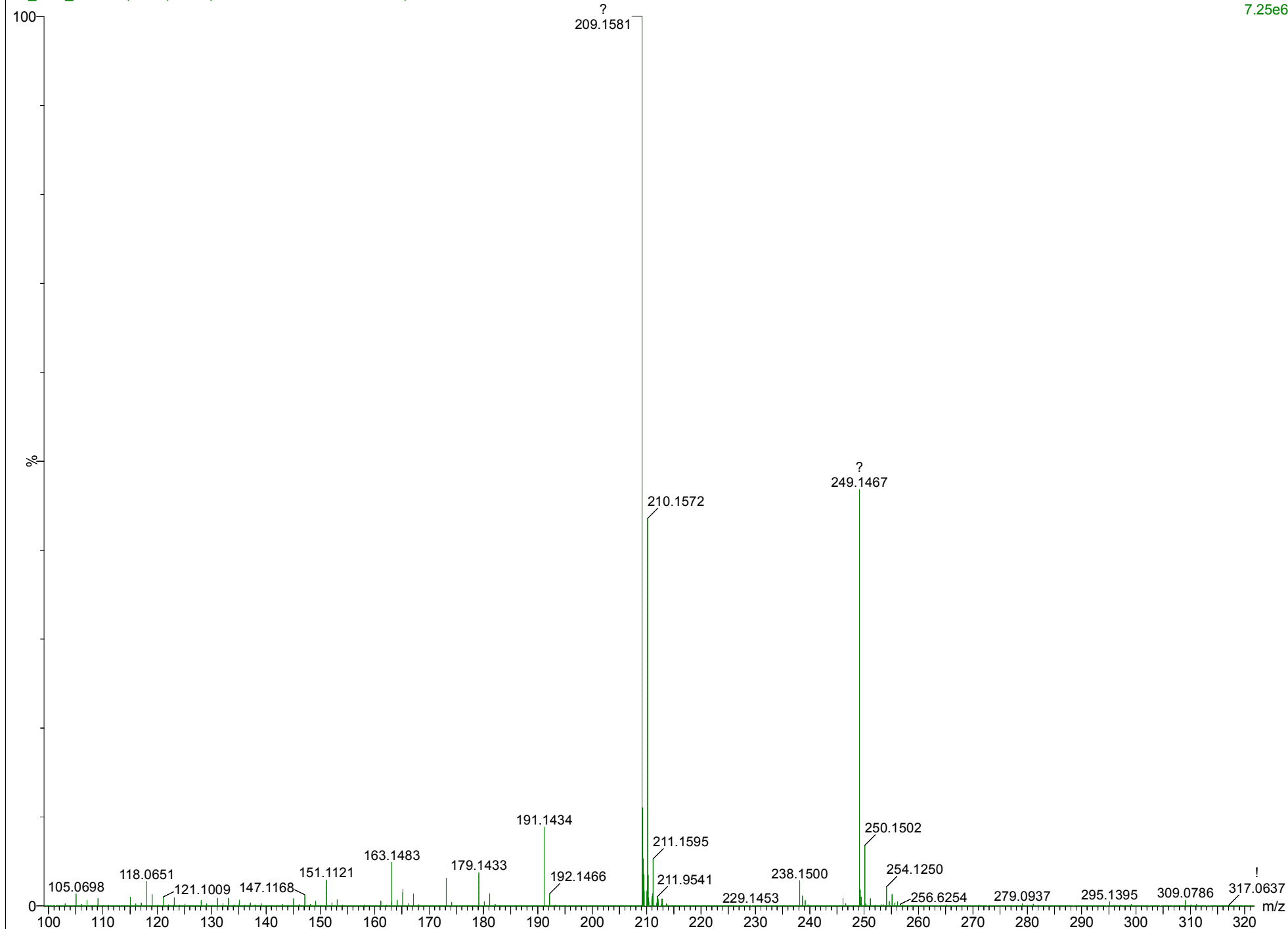

Supplement: Supplementary file 1 [file DataSheet1.ZIP › Supplementary Materials/Figure S45.HR-ESI-MS of Metabolite 11.pdf]

CARBON\_01  
VNS-600 CARBON 1118M5 IN cdcl3 Dec 26 2019

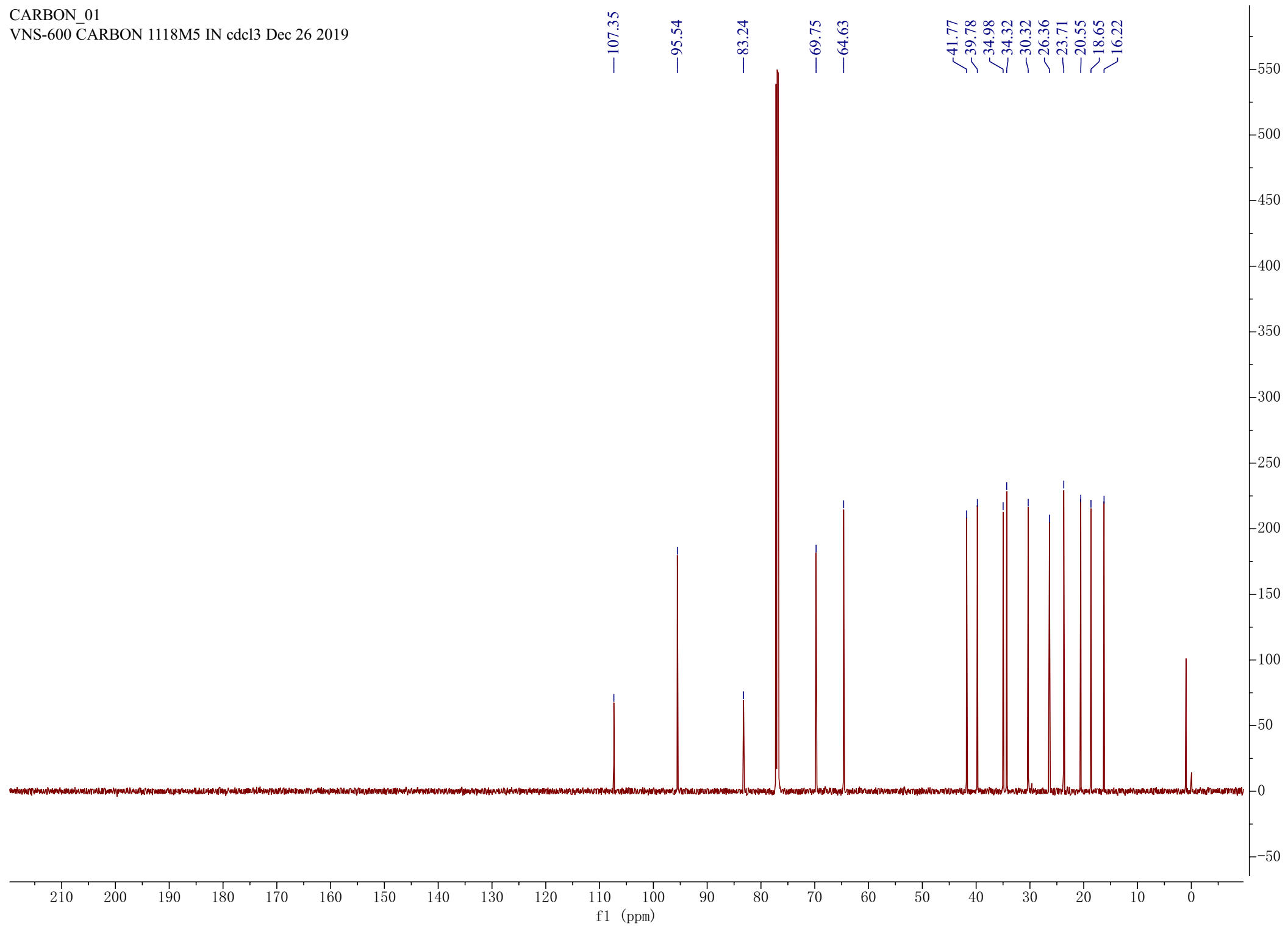

Supplement: Supplementary file 1 [file DataSheet1.ZIP › Supplementary Materials/Figure S46.C-NMR of Metabolite 12.pdf]

PROTON\_01  
VNS-600 PROTON 1118M5 IN cdc13 Nov 22 2019

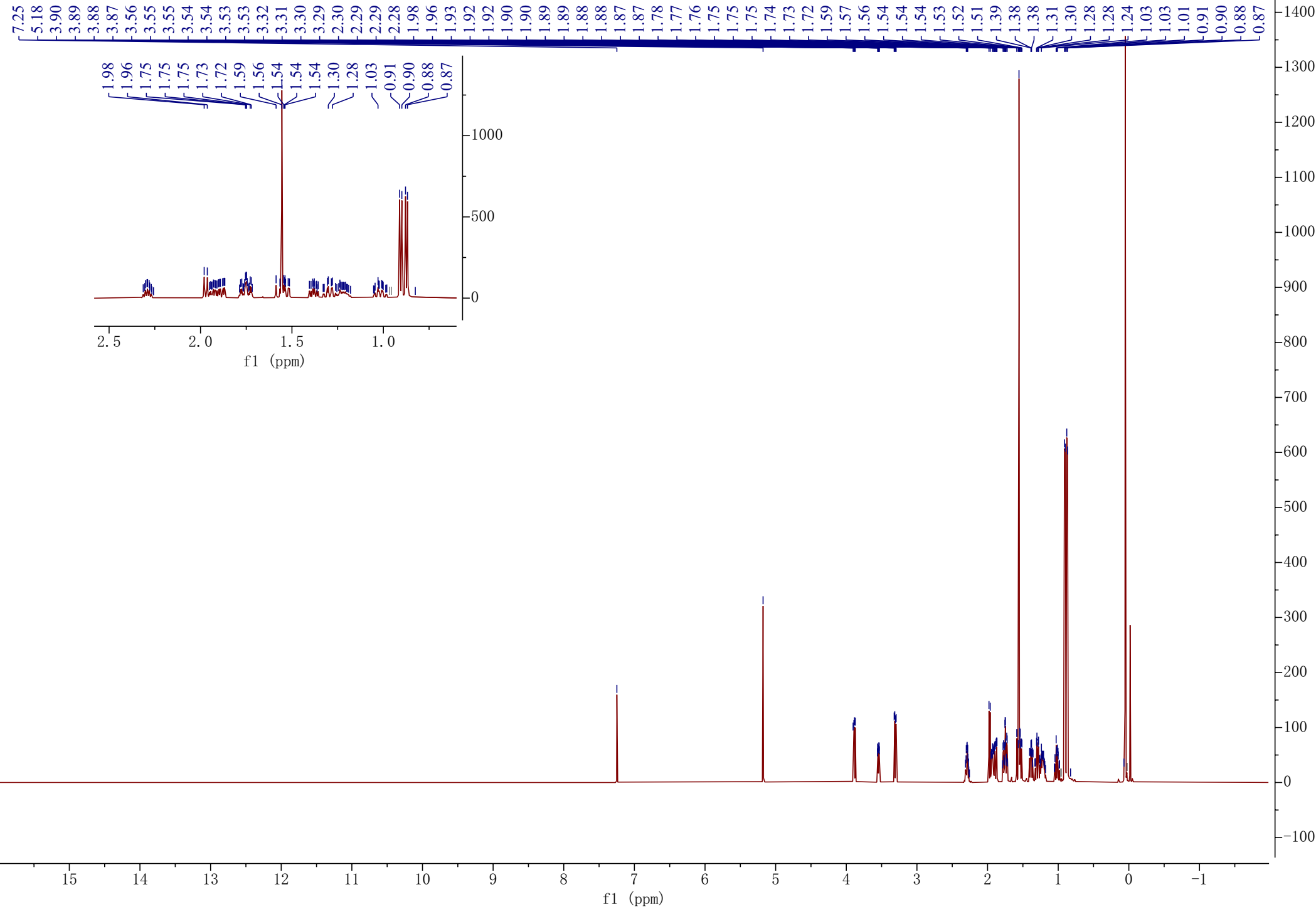

Supplement: Supplementary file 1 [file DataSheet1.ZIP › Supplementary Materials/Figure S47.H-NMR of Metabolite 12.pdf]

4

BY\_20200107M5P 726 (5.416) AM2 (Ar,22000.0,556.28,0.00,LS 10)

1: TOF MS ES+  
5.49e4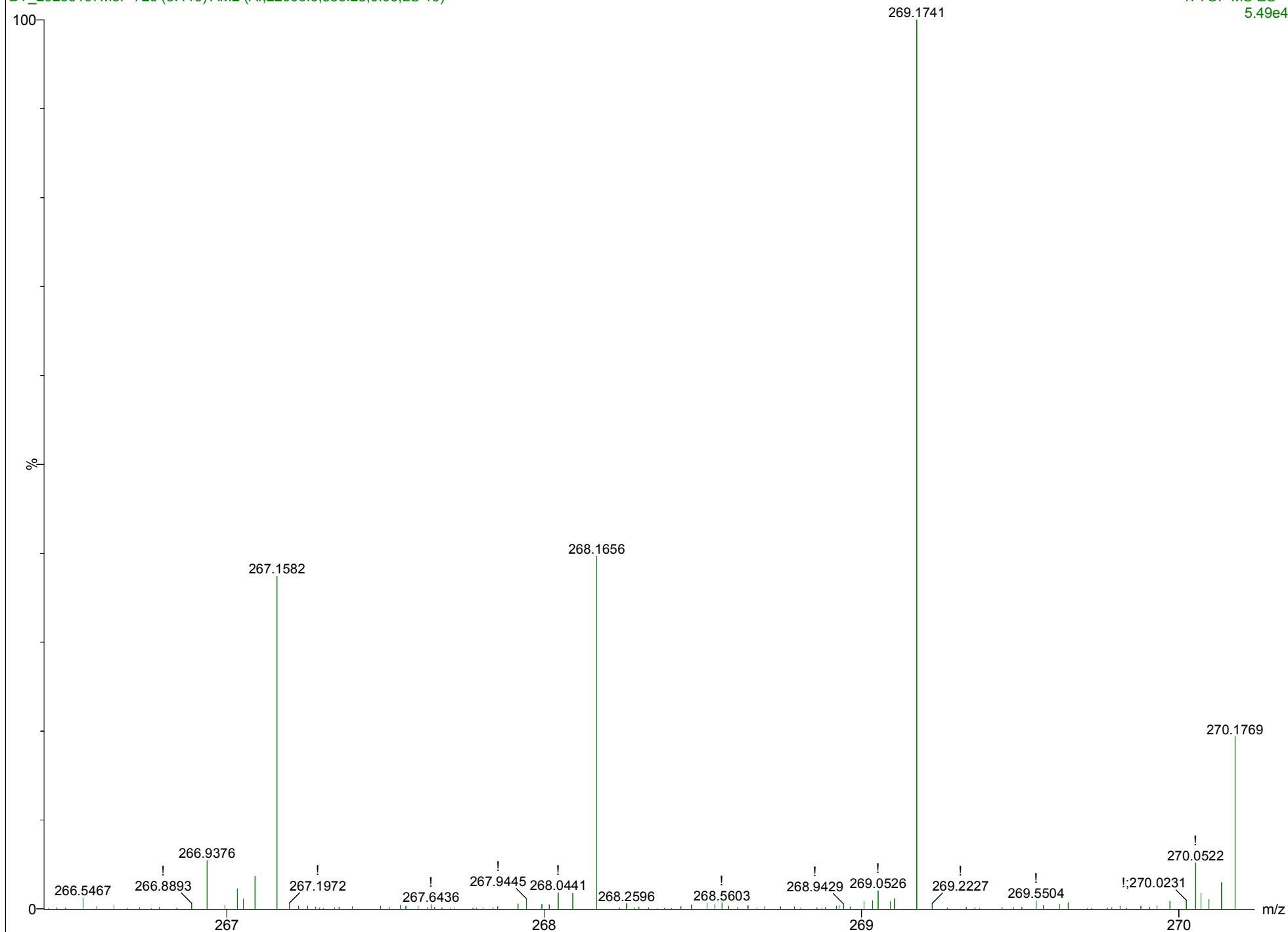

Supplement: Supplementary file 1 [file DataSheet1.ZIP › Supplementary Materials/Figure S48.HR-ESI-MS of Metabolite 12.pdf]

CARBON\_01  
VNS-600 CARBON 1216M6 IN cdcl3 Dec 25 2019

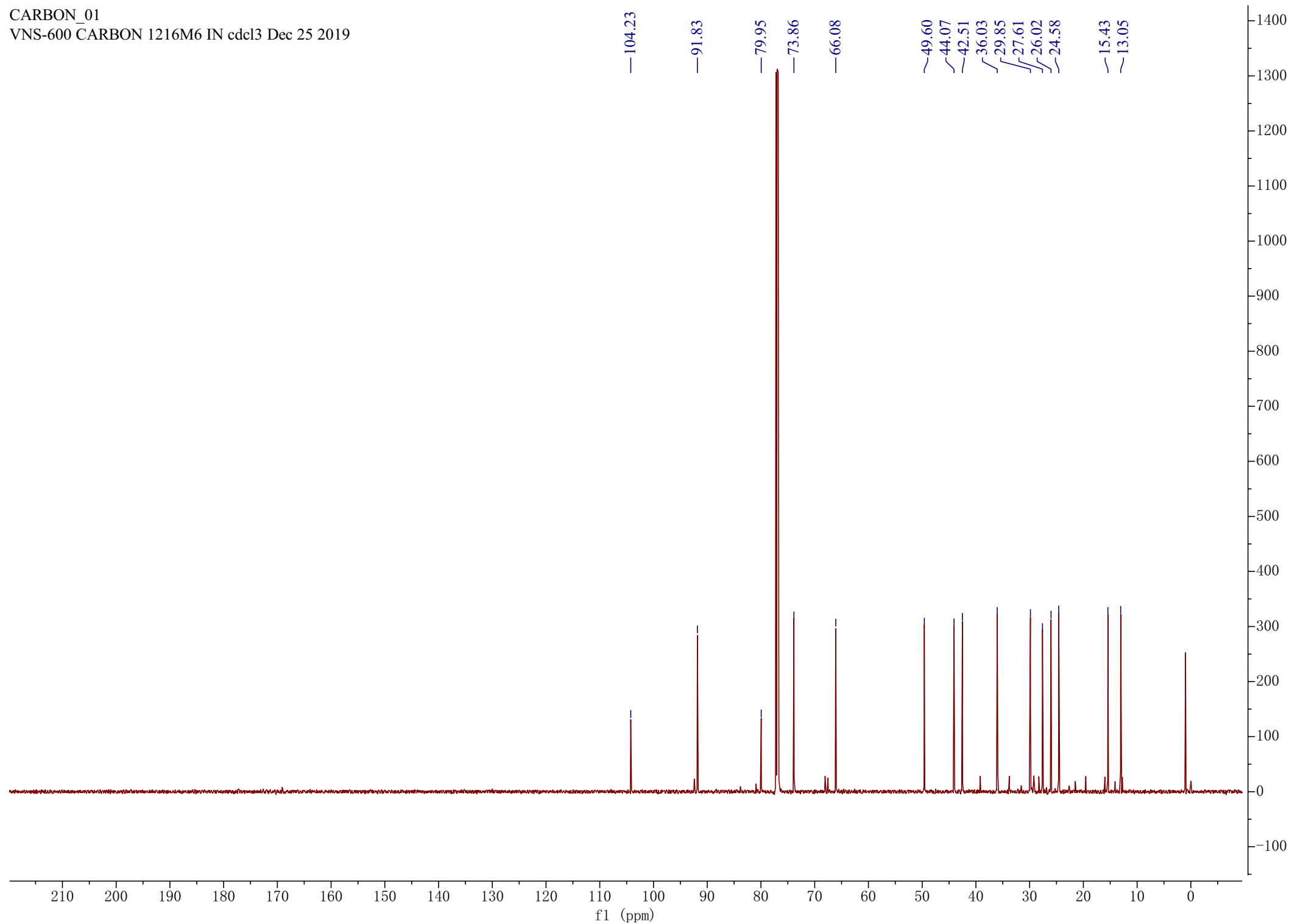

Supplement: Supplementary file 1 [file DataSheet1.ZIP › Supplementary Materials/Figure S49.C-NMR of Metabolite 13.pdf]

PROTON\_01  
VNS-600 PROTON Z1 IN dmso Jan 10 2019

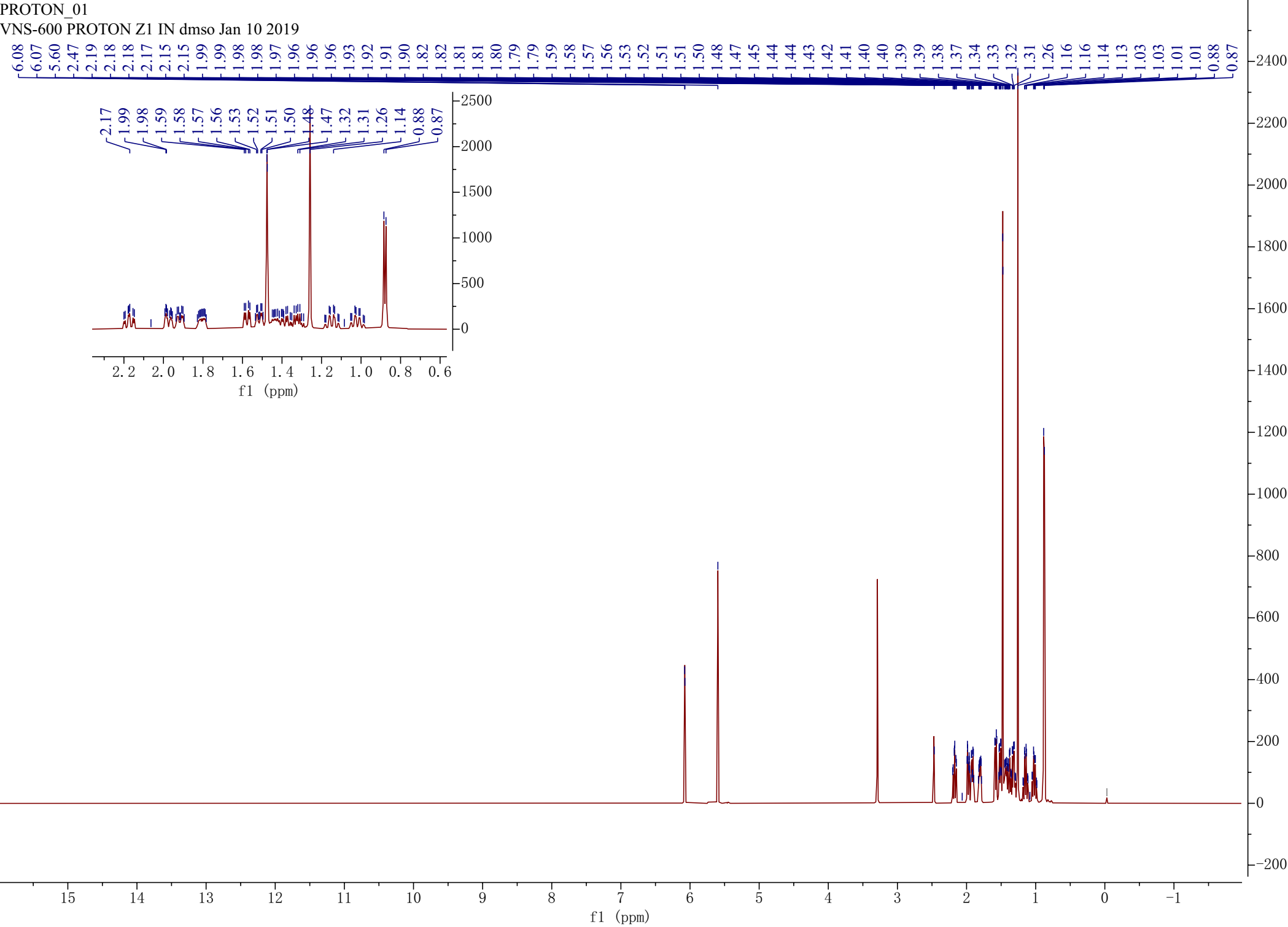

Supplement: Supplementary file 1 [file DataSheet1.ZIP › Supplementary Materials/Figure S5.H-NMR of Metabolite 3.pdf]

PROTON\_01  
VNS-600 PROTON 1216M6 IN cdcl3 Dec 25 2019

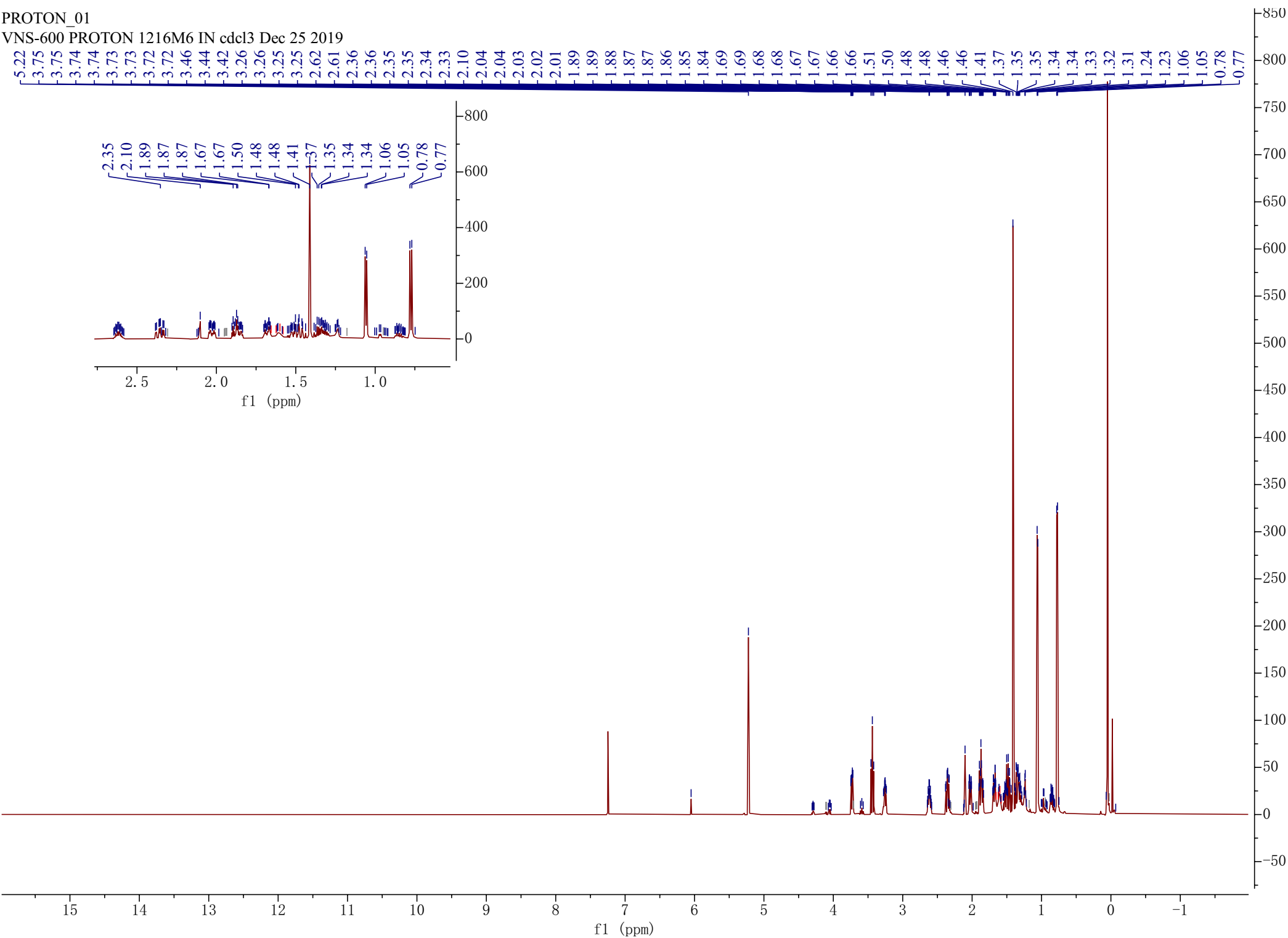

Supplement: Supplementary file 1 [file DataSheet1.ZIP › Supplementary Materials/Figure S50.H-NMR of Metabolite 13.pdf]

BY\_MT4 M6 628 (4.682) AM2 (Ar,22000.0,556.28,0.00,LS 10)

1: TOF MS ES+  
5.69e6

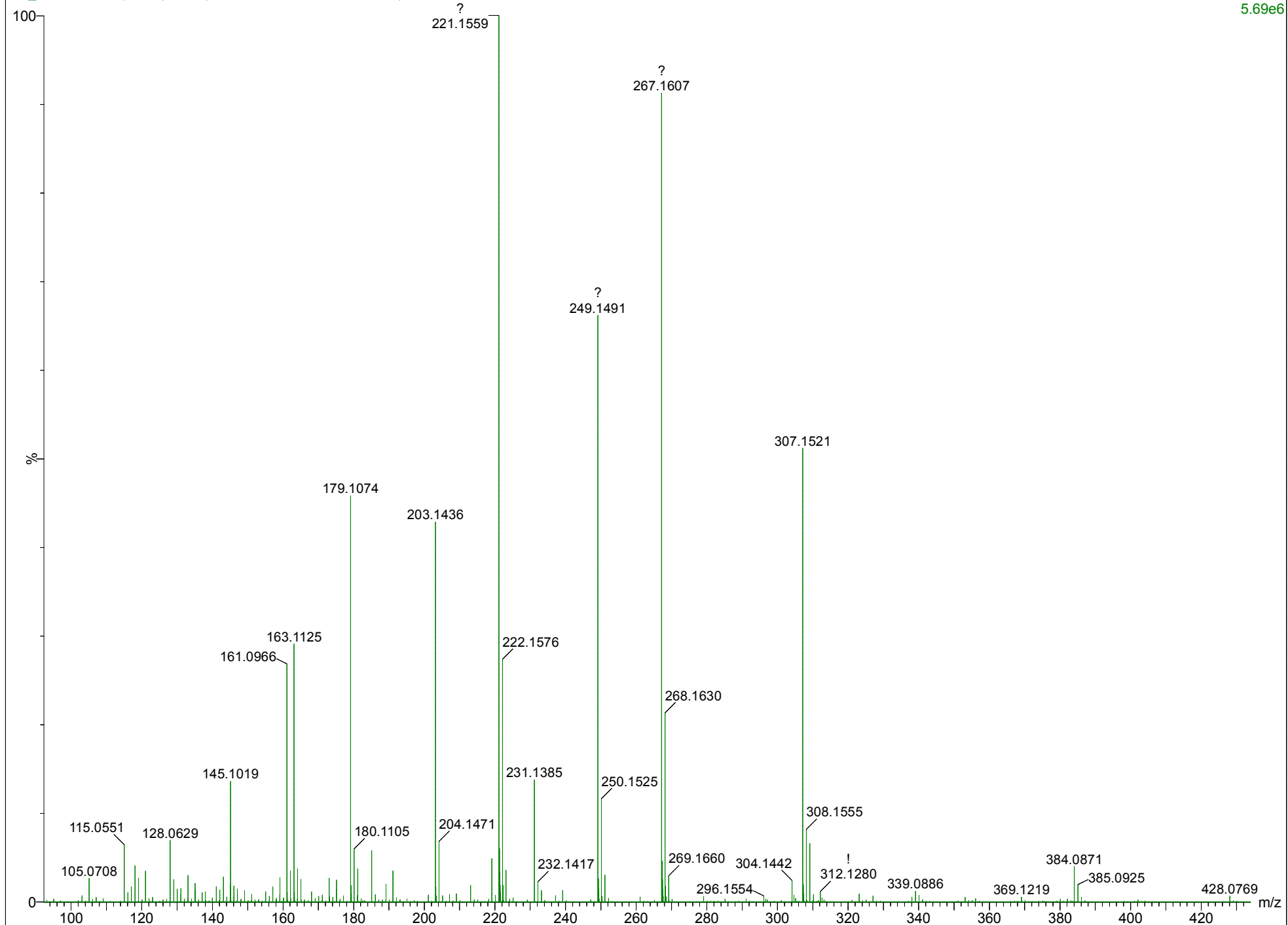

Supplement: Supplementary file 1 [file DataSheet1.ZIP › Supplementary Materials/Figure S51.HR-ESI-MS of Metabolite 13.pdf]

CARBON\_01  
VNS-600 CARBON 1216M8 IN cdcl3 Dec 26 2019

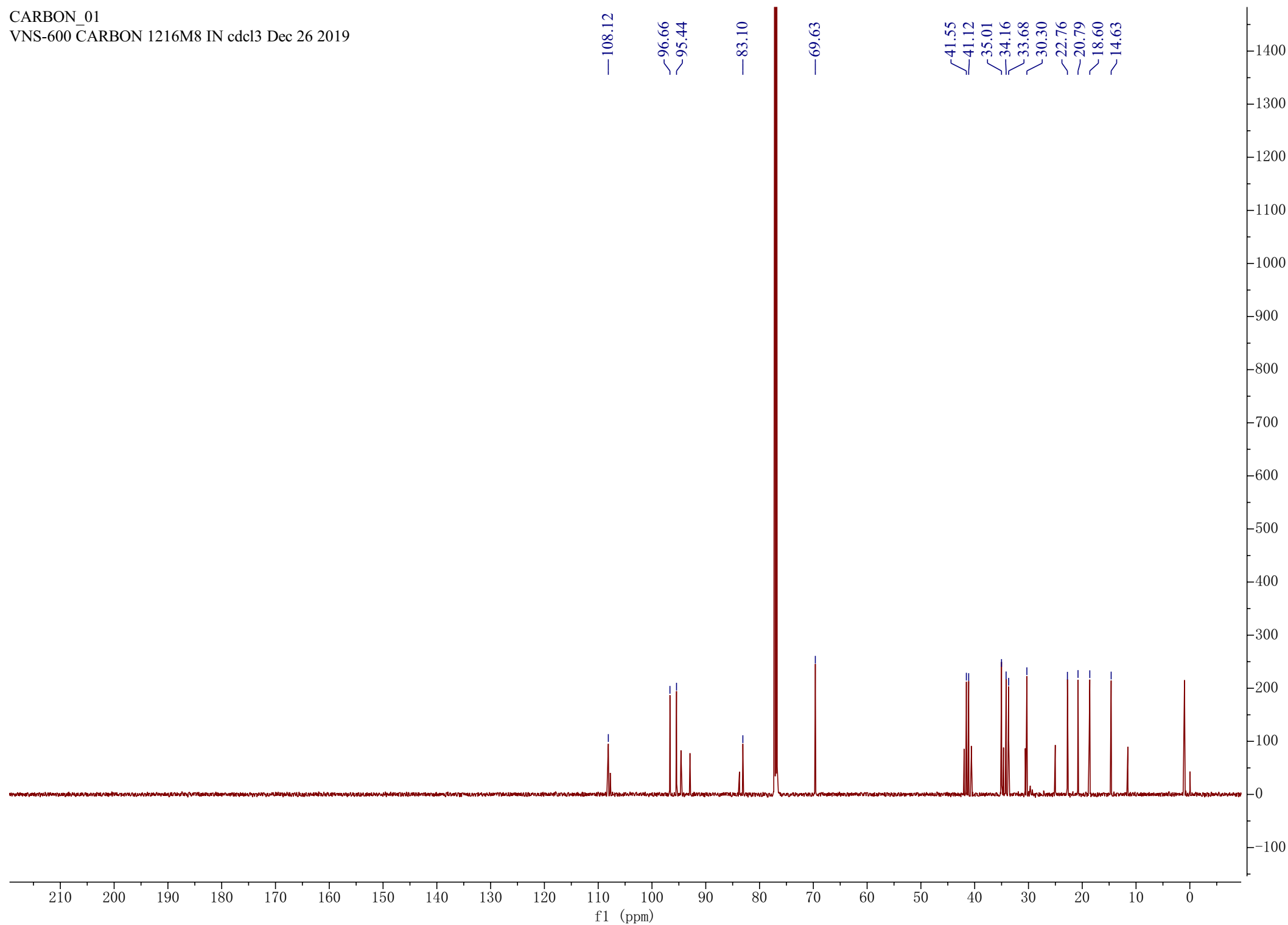

Supplement: Supplementary file 1 [file DataSheet1.ZIP › Supplementary Materials/Figure S52.C-NMR of Metabolite 14.pdf]

PROTON\_01  
VNS-600 PROTON 1216M8 IN cdcl3 Dec 26 2019

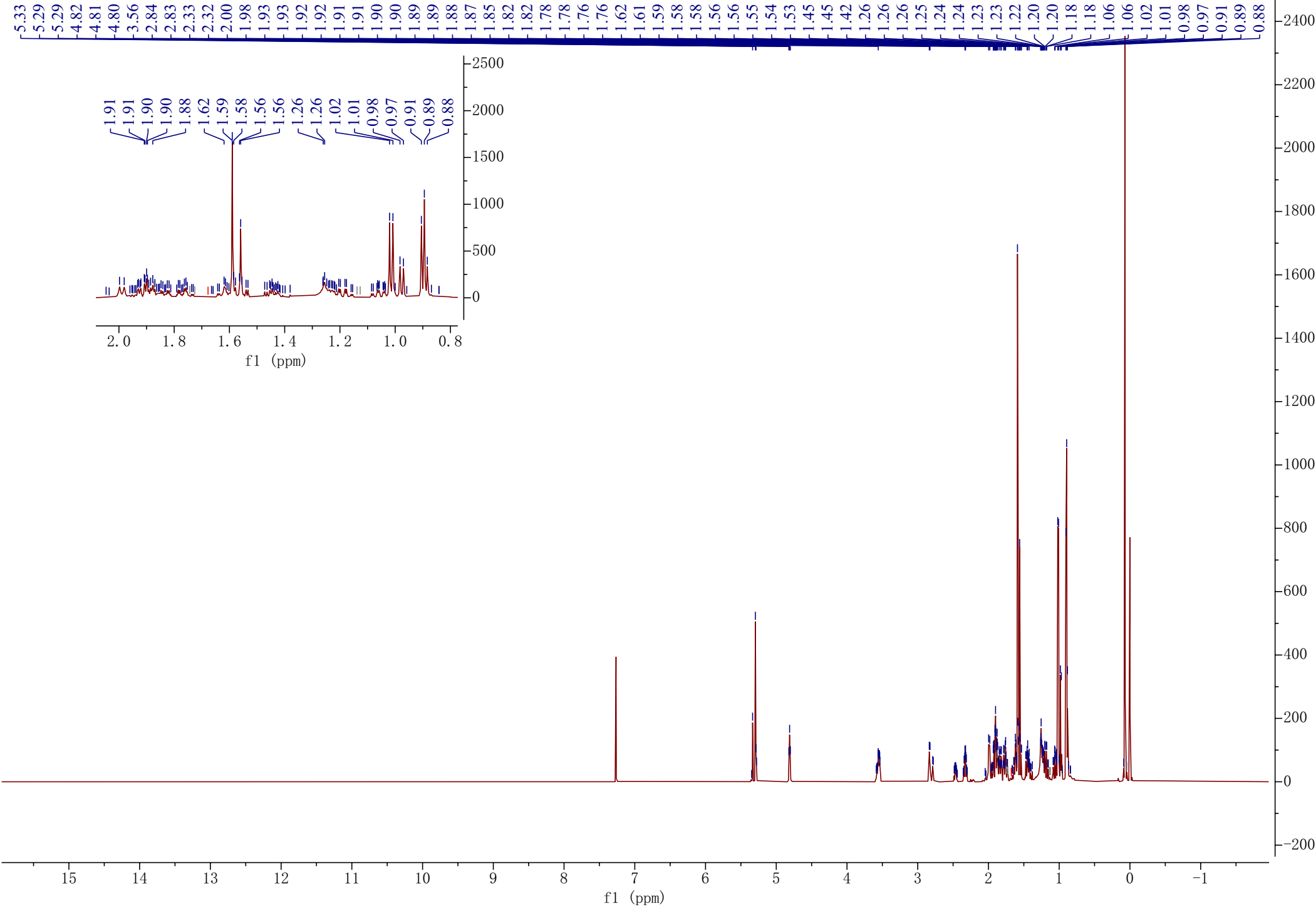

Supplement: Supplementary file 1 [file DataSheet1.ZIP › Supplementary Materials/Figure S53.H-NMR of Metabolite 14.pdf]

BY\_MT4 M8 558 (4.163) AM2 (Ar,22000.0,556.28,0.00,LS 10)

1: TOF MS ES+  
5.71e6

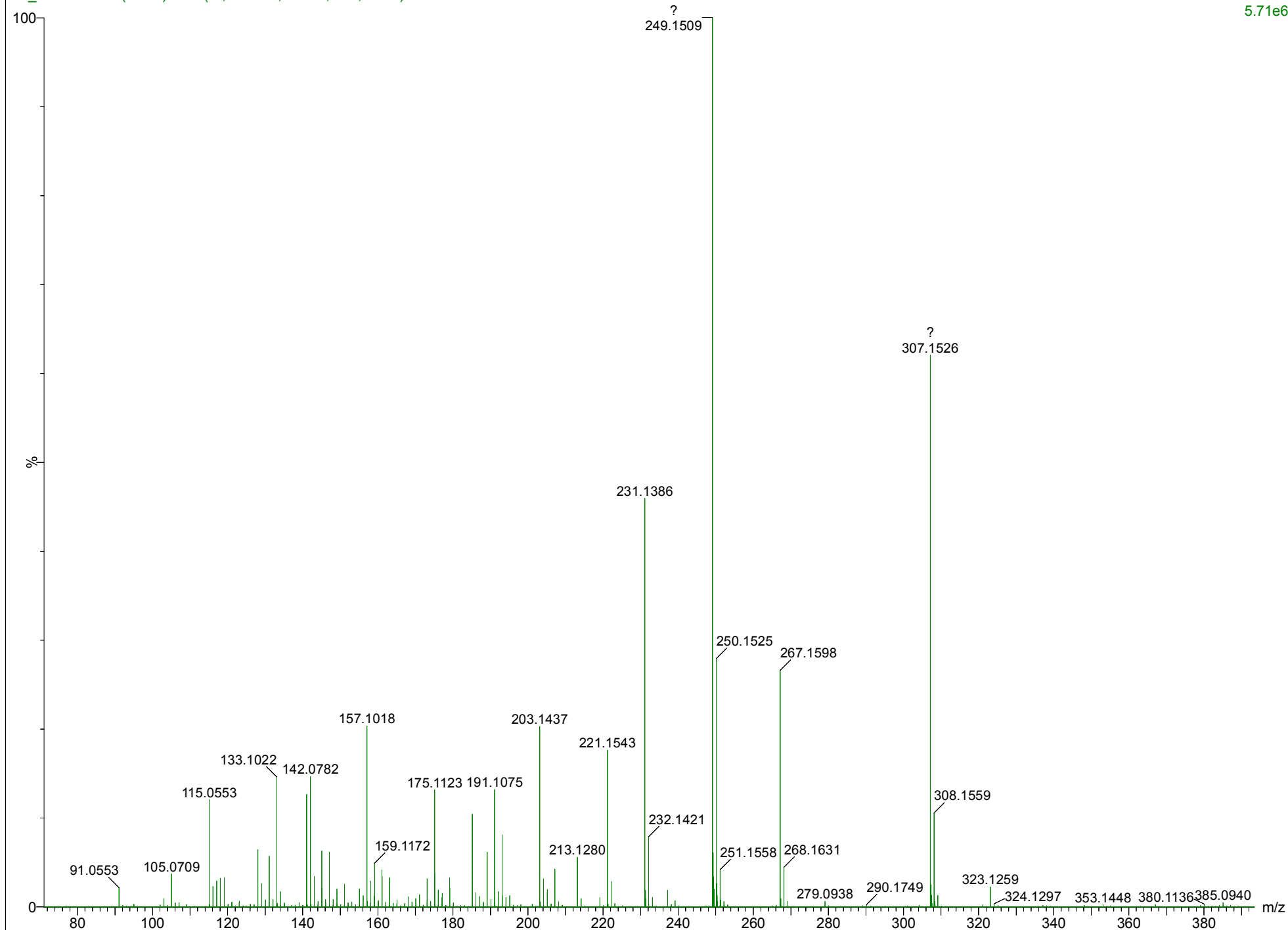

Supplement: Supplementary file 1 [file DataSheet1.ZIP › Supplementary Materials/Figure S54.HR-ESI-MS of Metabolite 14.pdf]

CARBON\_01  
VNS-600 CARBON M16 IN cdcl3 Sep 25 2020

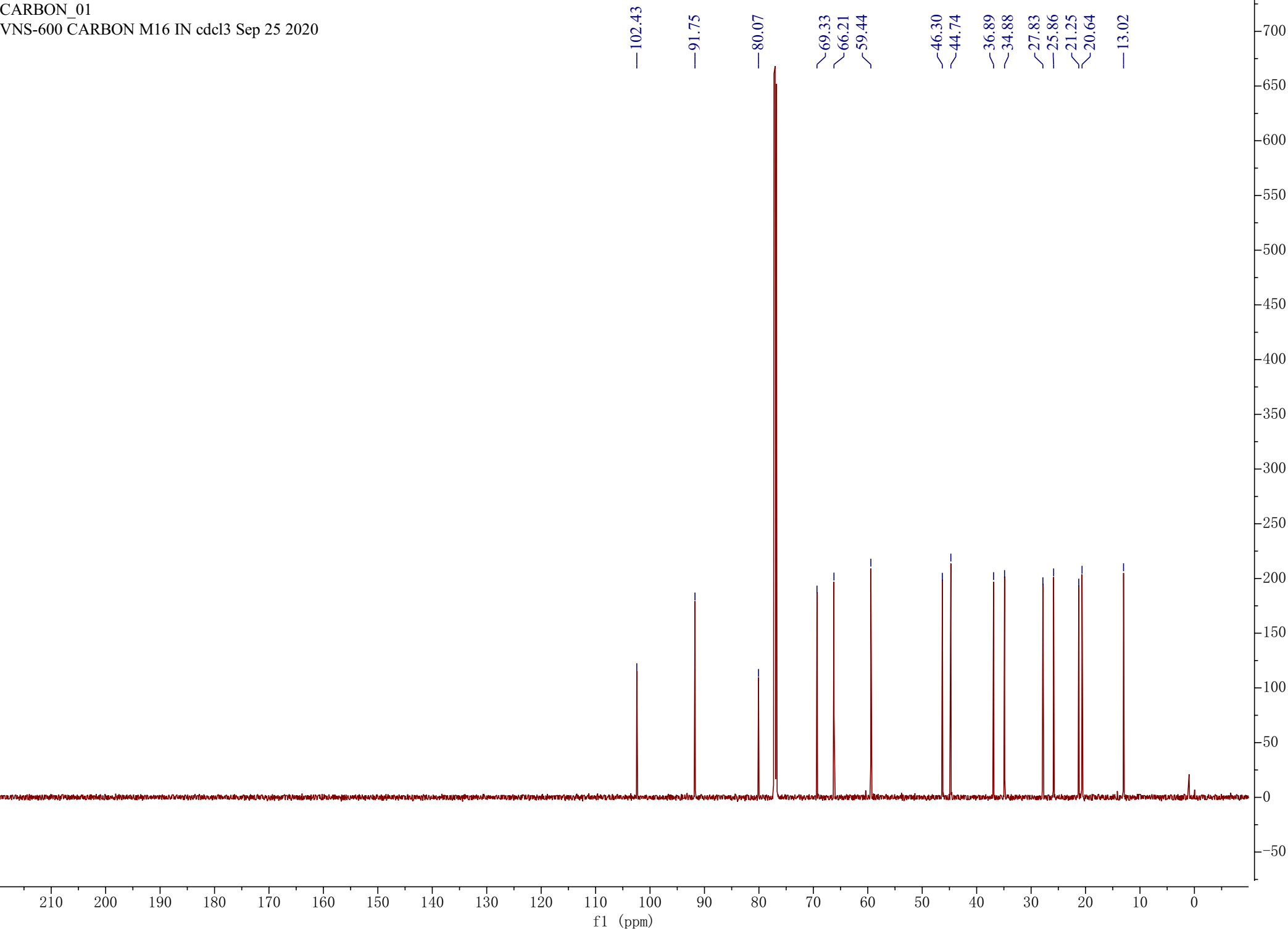

Supplement: Supplementary file 1 [file DataSheet1.ZIP › Supplementary Materials/Figure S55.C-NMR of Metabolite 15.pdf]

PROTON\_01  
VNS-600 PROTON M16 IN cdcl3 Sep 25 2020

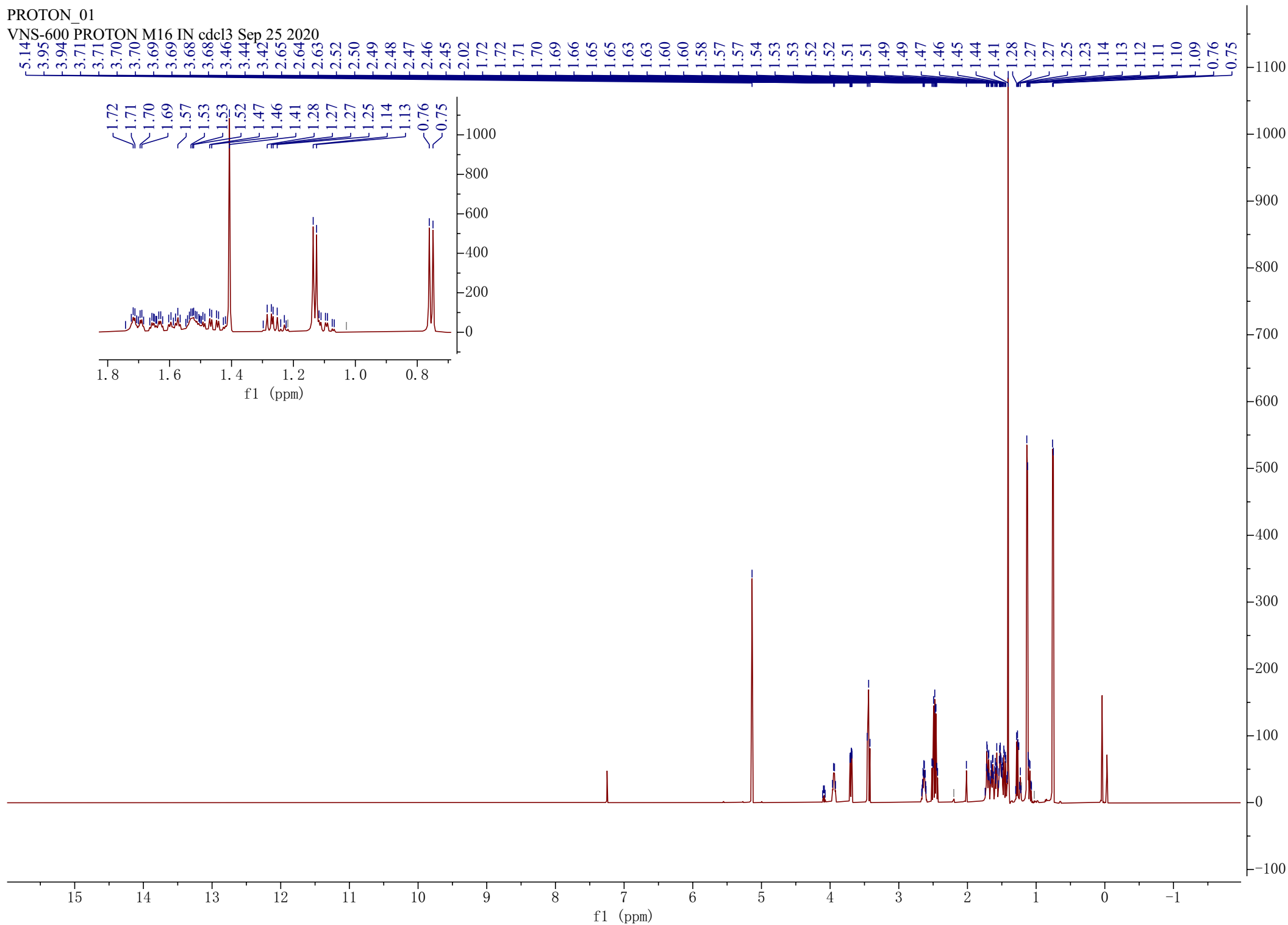

Supplement: Supplementary file 1 [file DataSheet1.ZIP › Supplementary Materials/Figure S56.H-NMR of Metabolite 15.pdf]

7

BY\_20200107M16P 663 (4.946) AM2 (Ar,22000.0,556.28,0.00,LS 10)

1: TOF MS ES+  
2.57e6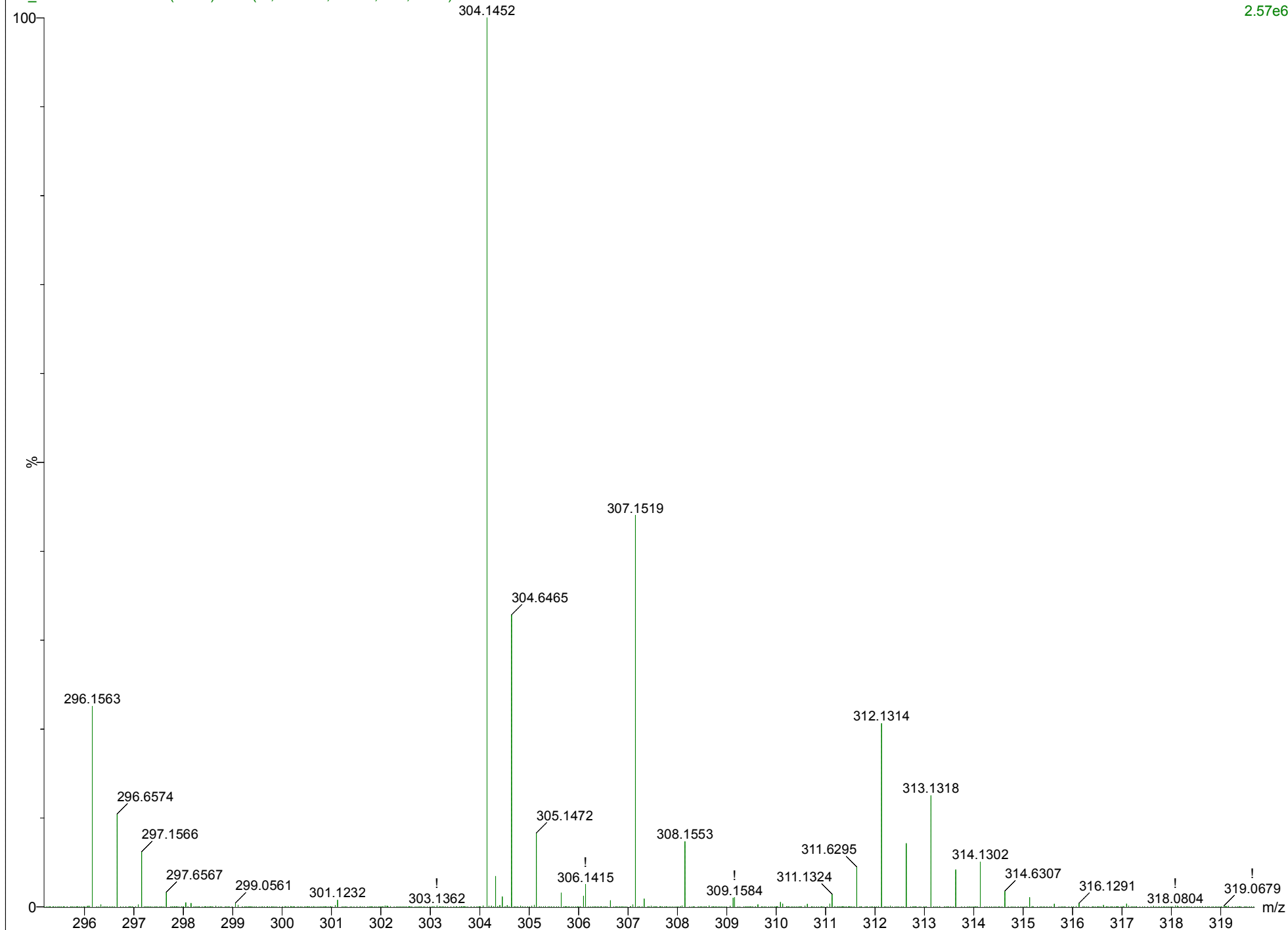

Supplement: Supplementary file 1 [file DataSheet1.ZIP › Supplementary Materials/Figure S57.HR-ESI-MS of Metabolite 15.pdf]

20201021 1020M19/2  
Bruker AVIII HD 600  
C13 CDC13 D:\ DATA2020 14

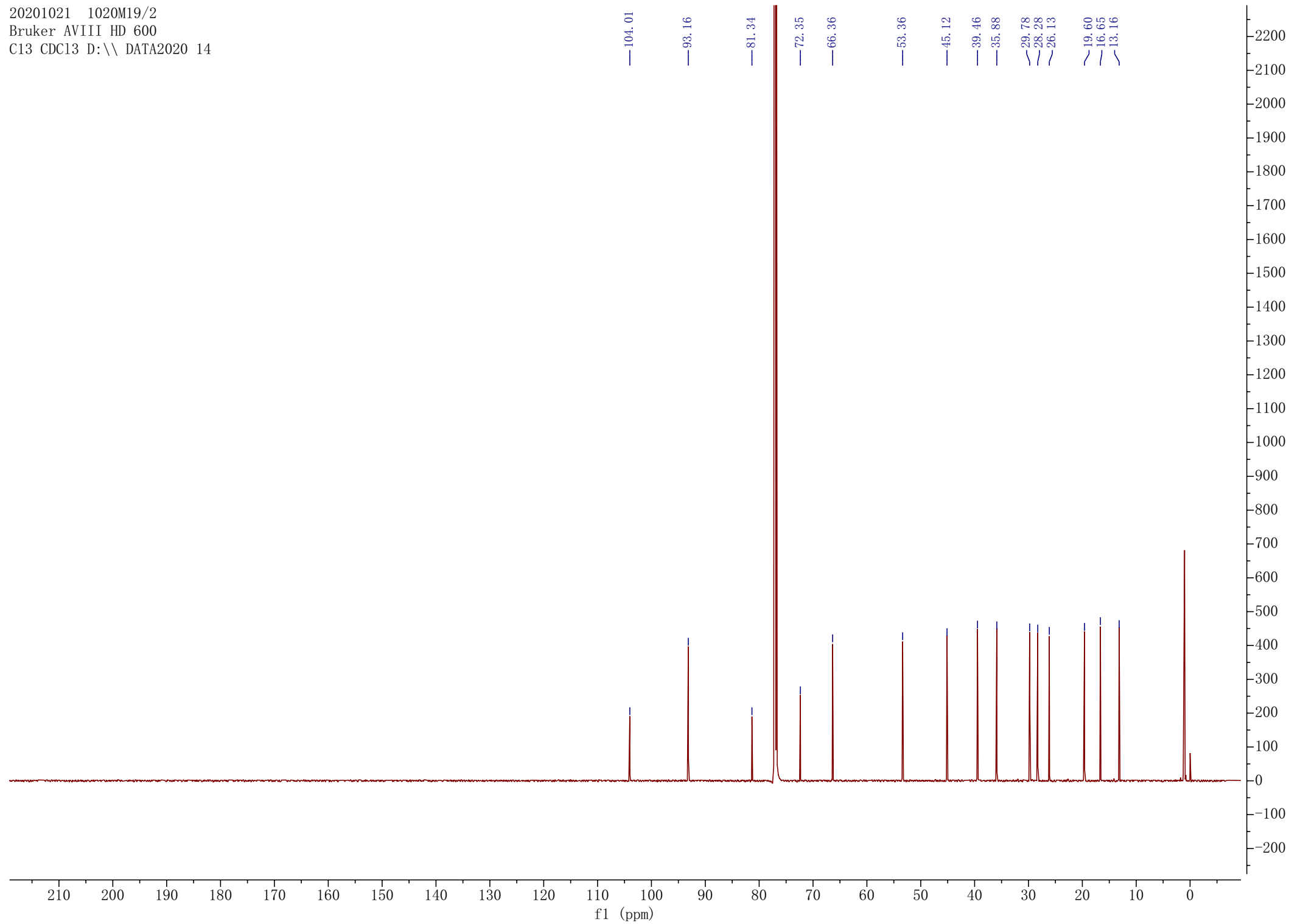

Supplement: Supplementary file 1 [file DataSheet1.ZIP › Supplementary Materials/Figure S58.C-NMR of Metabolite 16.pdf]

M19-MT3.1.fid  
Bruker AVIII HD 600  
PROTON CDCI3 D:\DATA2020 14

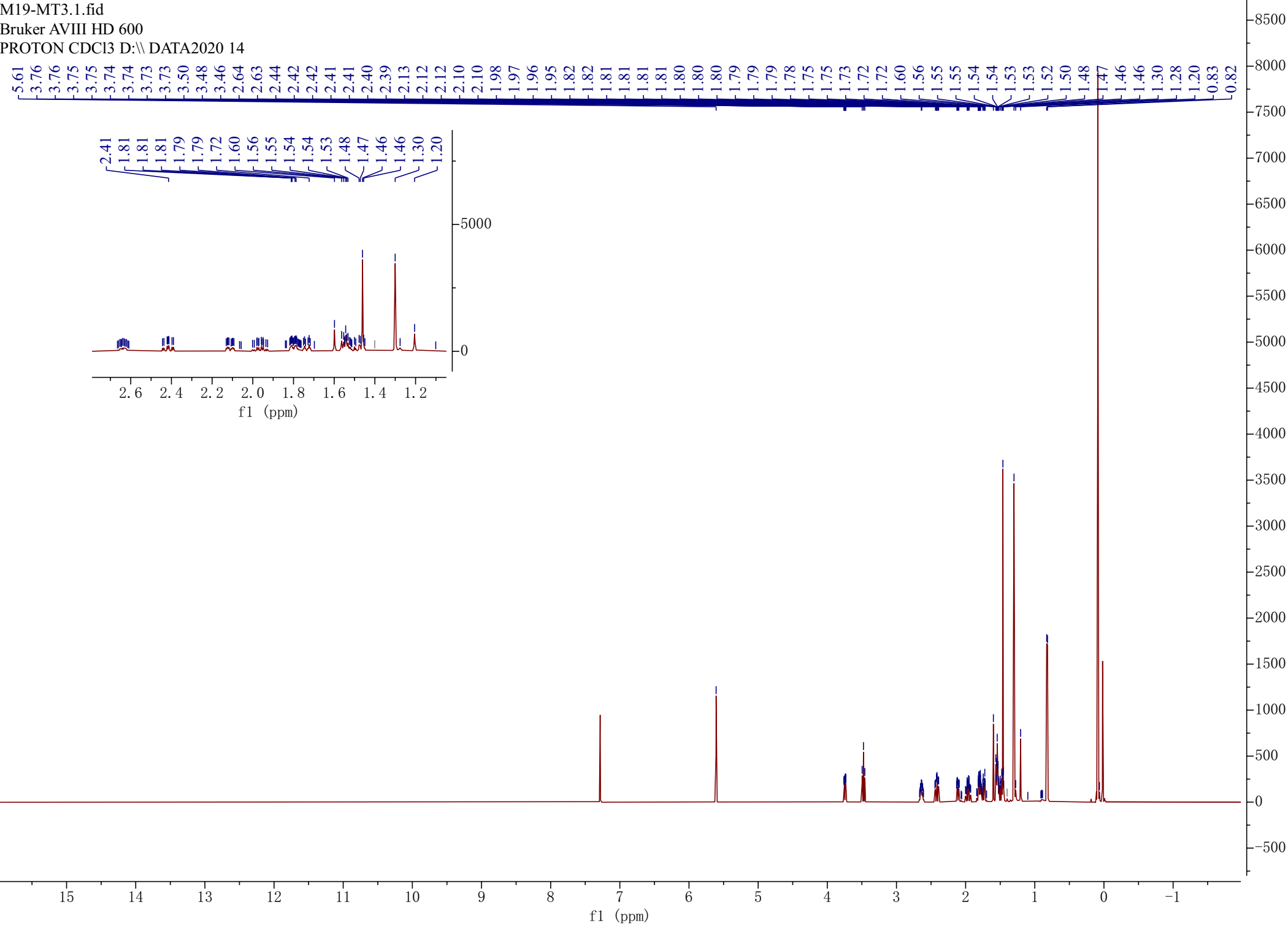

Supplement: Supplementary file 1 [file DataSheet1.ZIP › Supplementary Materials/Figure S59.H-NMR of Metabolite 16.pdf]

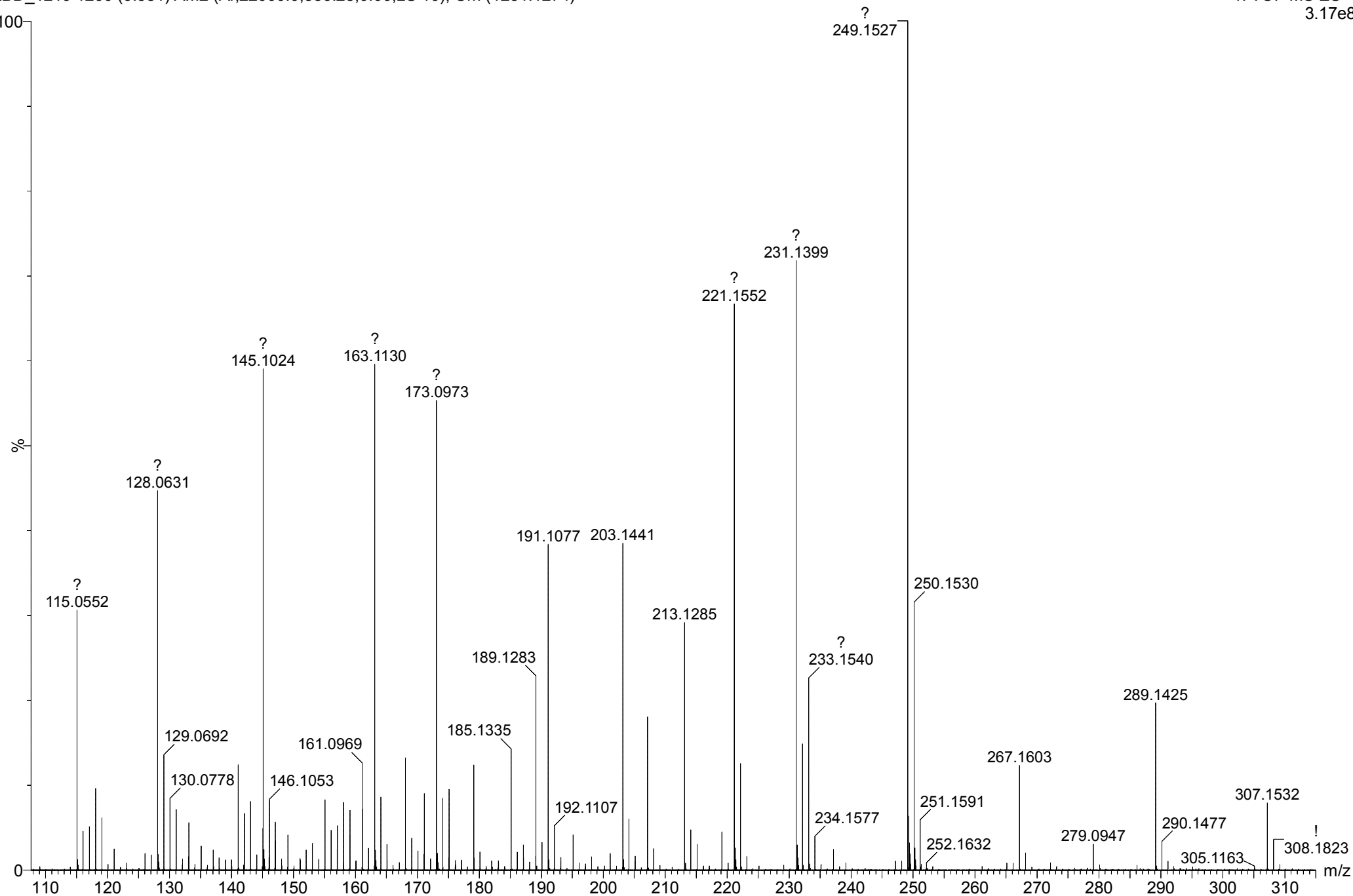

Supplement: Supplementary file 1 [file DataSheet1.ZIP › Supplementary Materials/Figure S6.HR-ESI-MS of Metabolite 3.pdf]

10

BY\_20200107M19P 666 (4.967) AM2 (Ar,22000.0,556.28,0.00,LS 10)

1: TOF MS ES+  
3.46e6

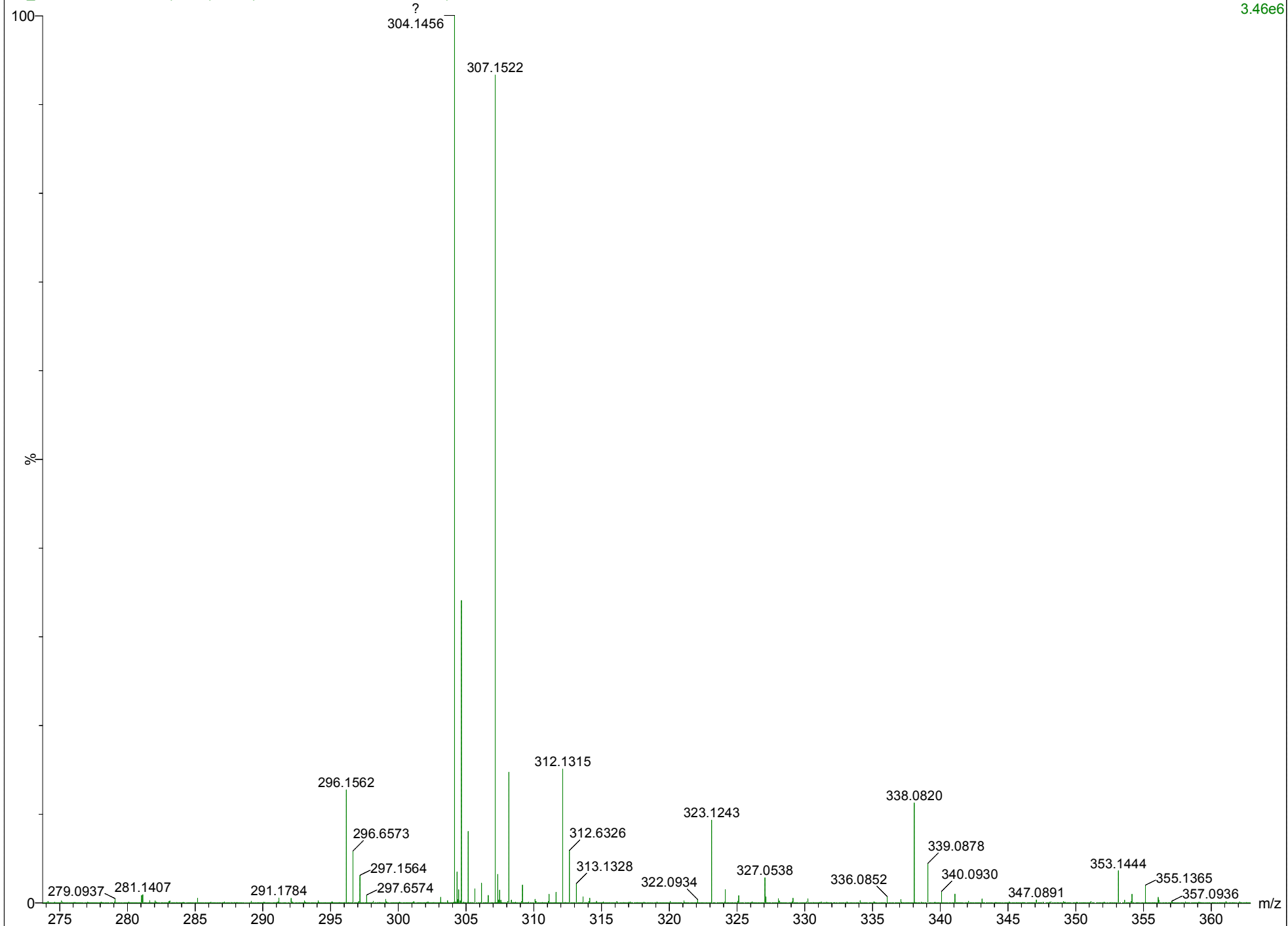

Supplement: Supplementary file 1 [file DataSheet1.ZIP › Supplementary Materials/Figure S60.HR-ESI-MS of Metabolite 16.pdf]

CARBON\_01  
VNS-600 CARBON 1110M22 IN cdcl3 Nov 12 2020

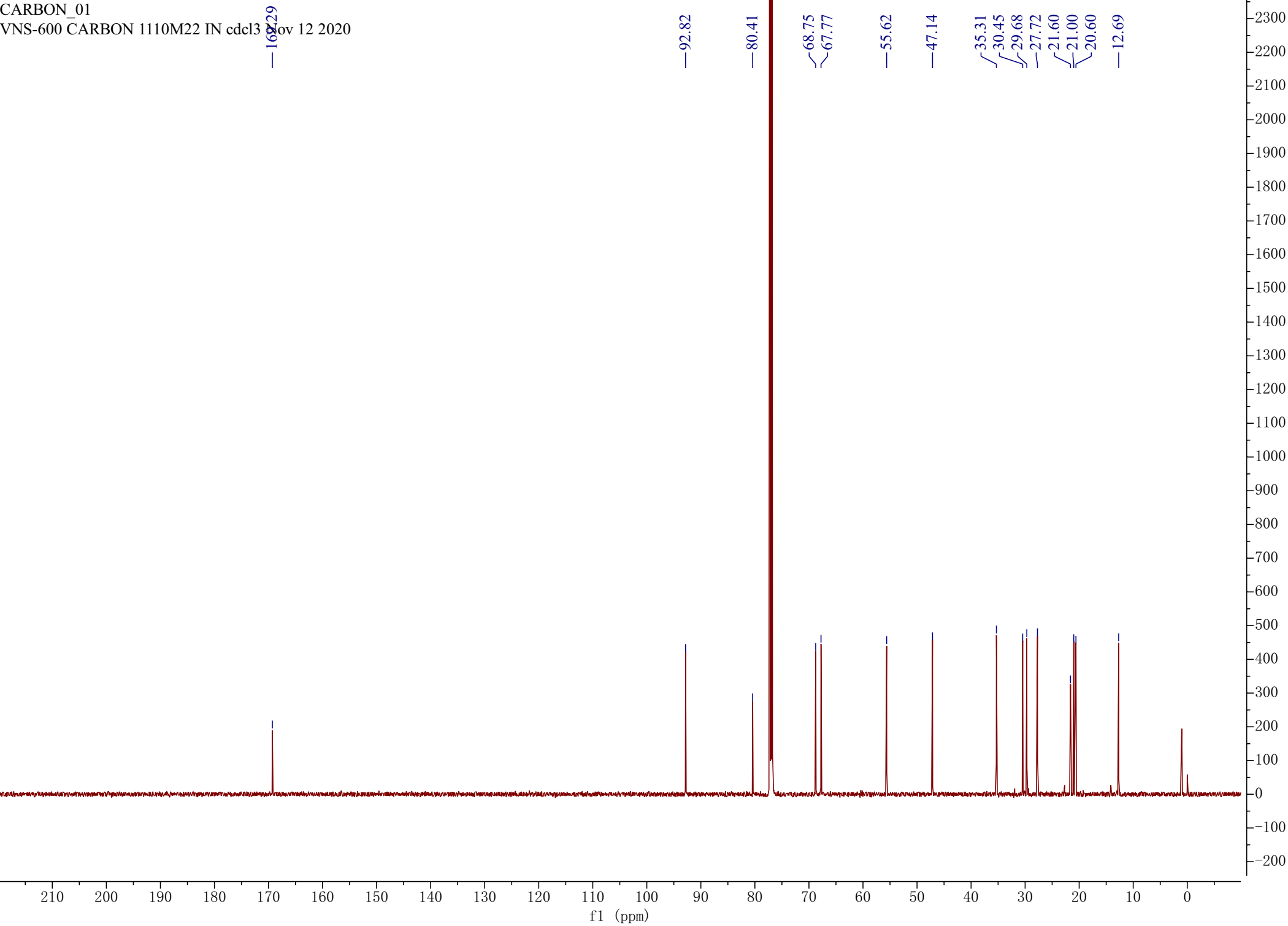

Supplement: Supplementary file 1 [file DataSheet1.ZIP › Supplementary Materials/Figure S61.C-NMR of Metabolite 17.pdf]

PROTON\_01  
VNS-600 PROTON 1110M22 IN cdcl3 Nov 12 2020

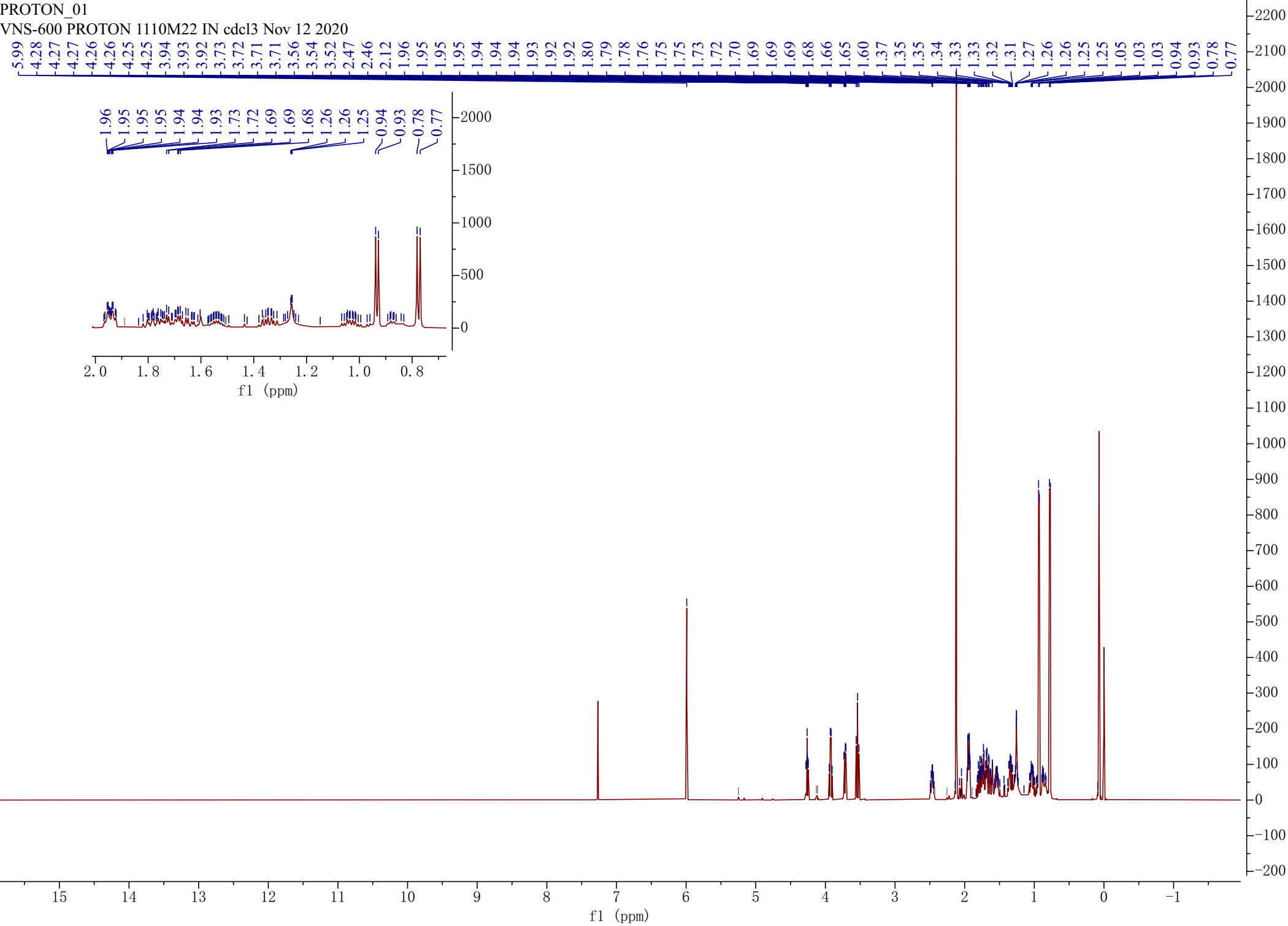

Supplement: Supplementary file 1 [file DataSheet1.ZIP › Supplementary Materials/Figure S62.H-NMR of Metabolite 17.pdf]

12

BY\_20200107M22P 916 (6.825) AM2 (Ar,22000.0,556.28,0.00,LS 10)

1: TOF MS ES+  
2.57e6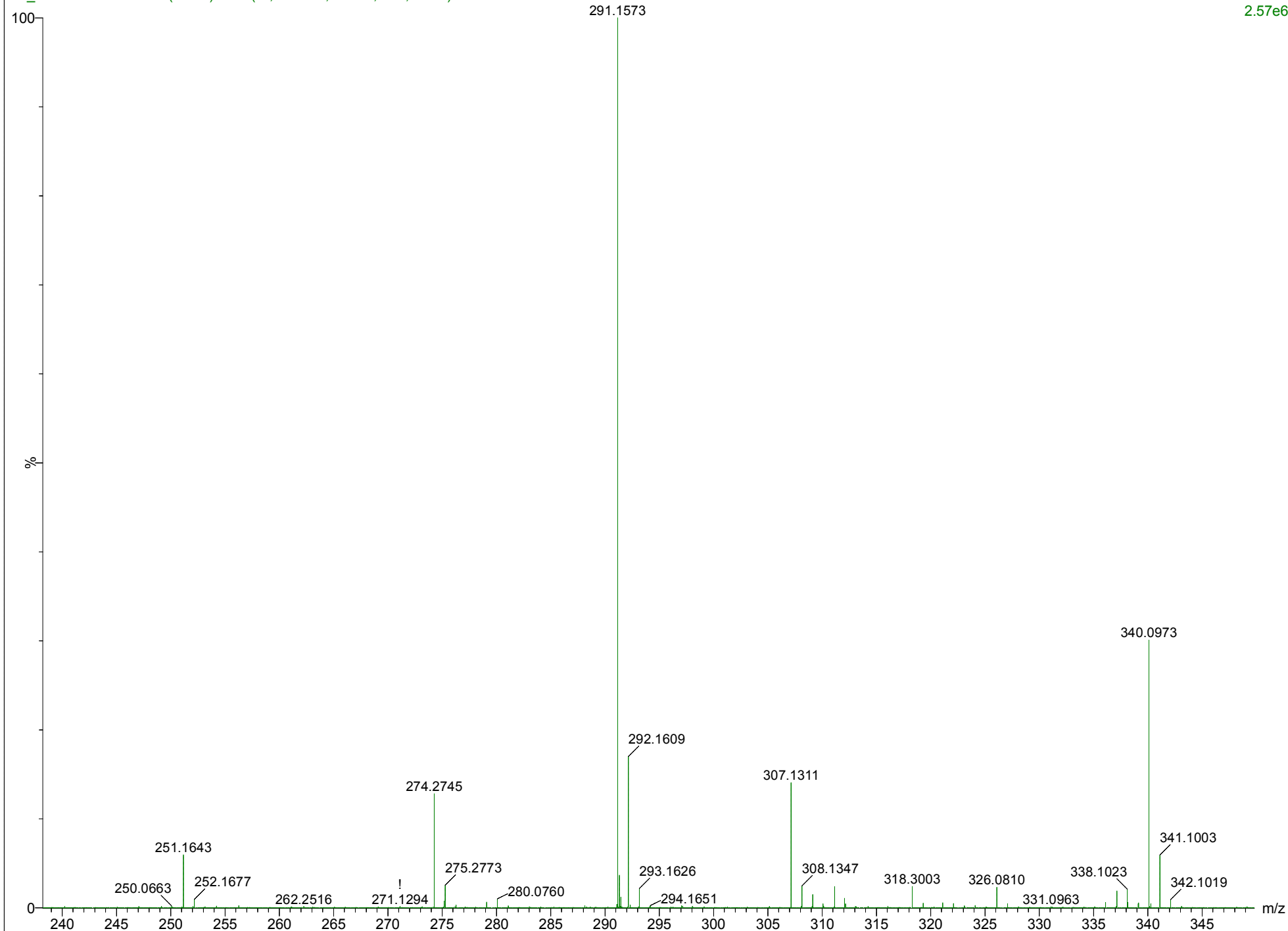

Supplement: Supplementary file 1 [file DataSheet1.ZIP › Supplementary Materials/Figure S63.HR-ESI-MS of Metabolite 17.pdf]

M24-MT3.2.fid  
C13CPD CDCl3 D:\nmrsu 27

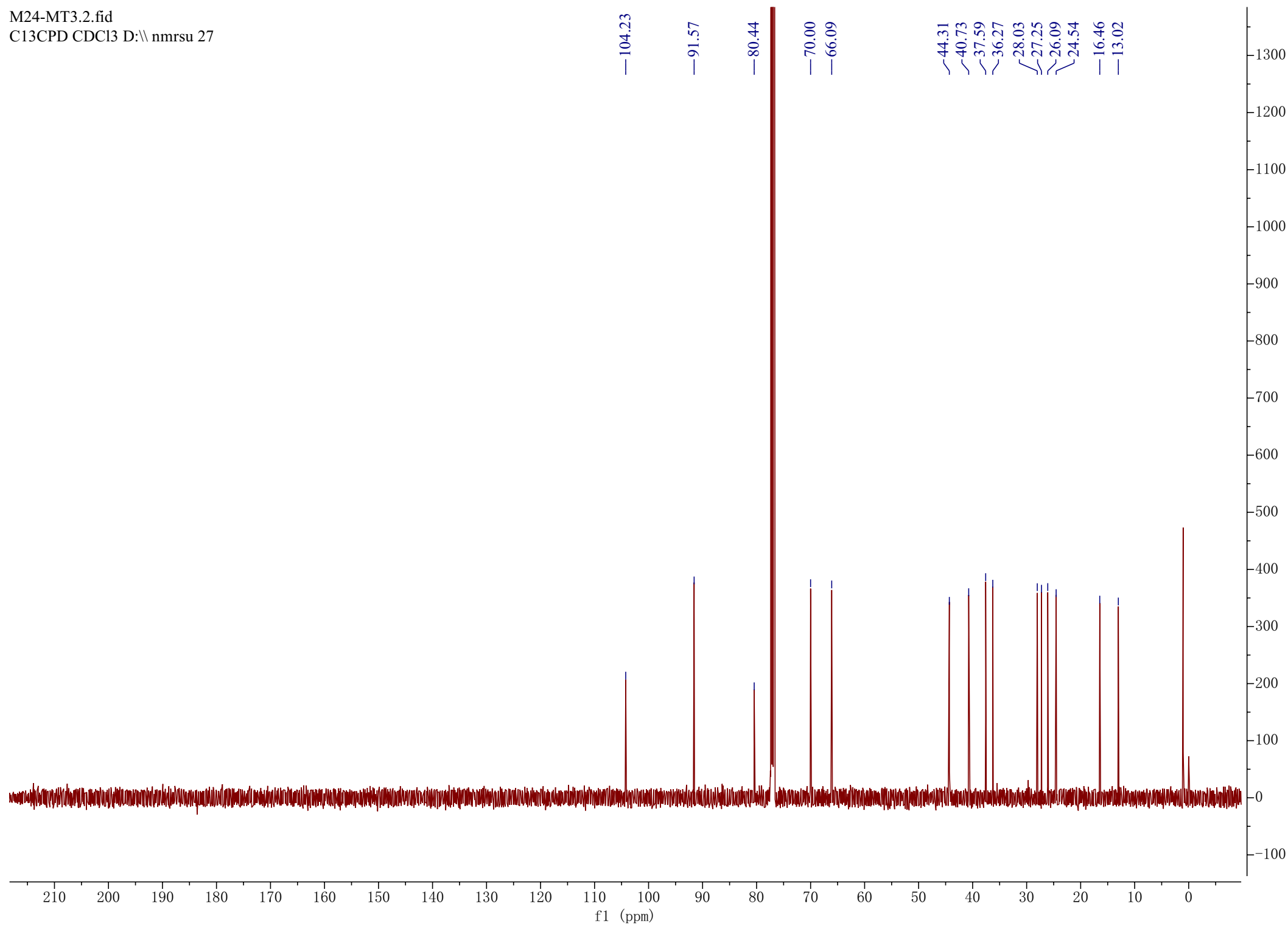

Supplement: Supplementary file 1 [file DataSheet1.ZIP › Supplementary Materials/Figure S64.C-NMR of Metabolite 18.pdf]

M24-MT3.1.fid  
PROTON CDC13 D:\nmrsu 27

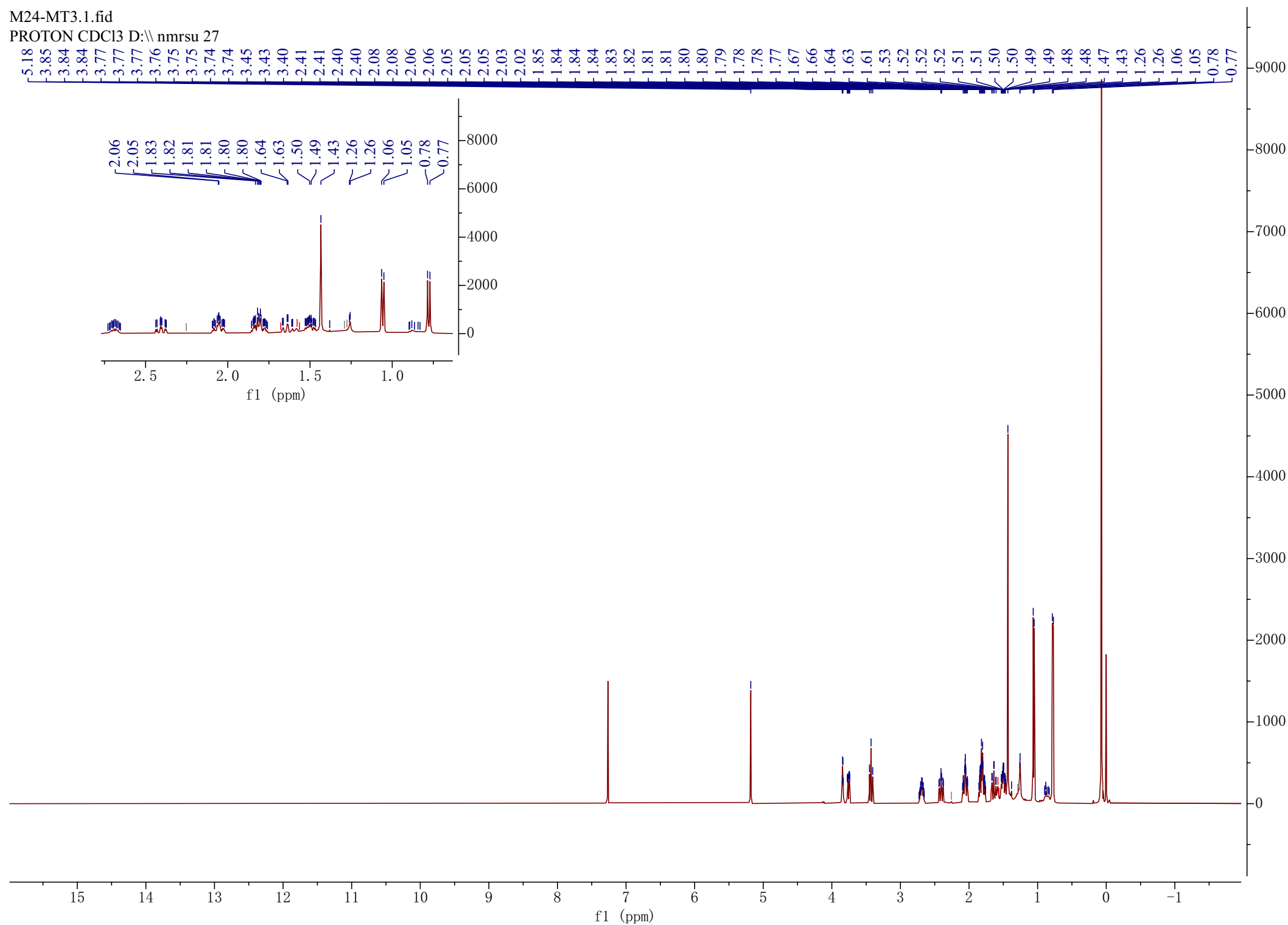

Supplement: Supplementary file 1 [file DataSheet1.ZIP › Supplementary Materials/Figure S65.H-NMR of Metabolite 18.pdf]

14

BY\_20200107M24P 513 (3.828) AM2 (Ar,22000.0,556.28,0.00,LS 10)

1: TOF MS ES+  
2.42e6

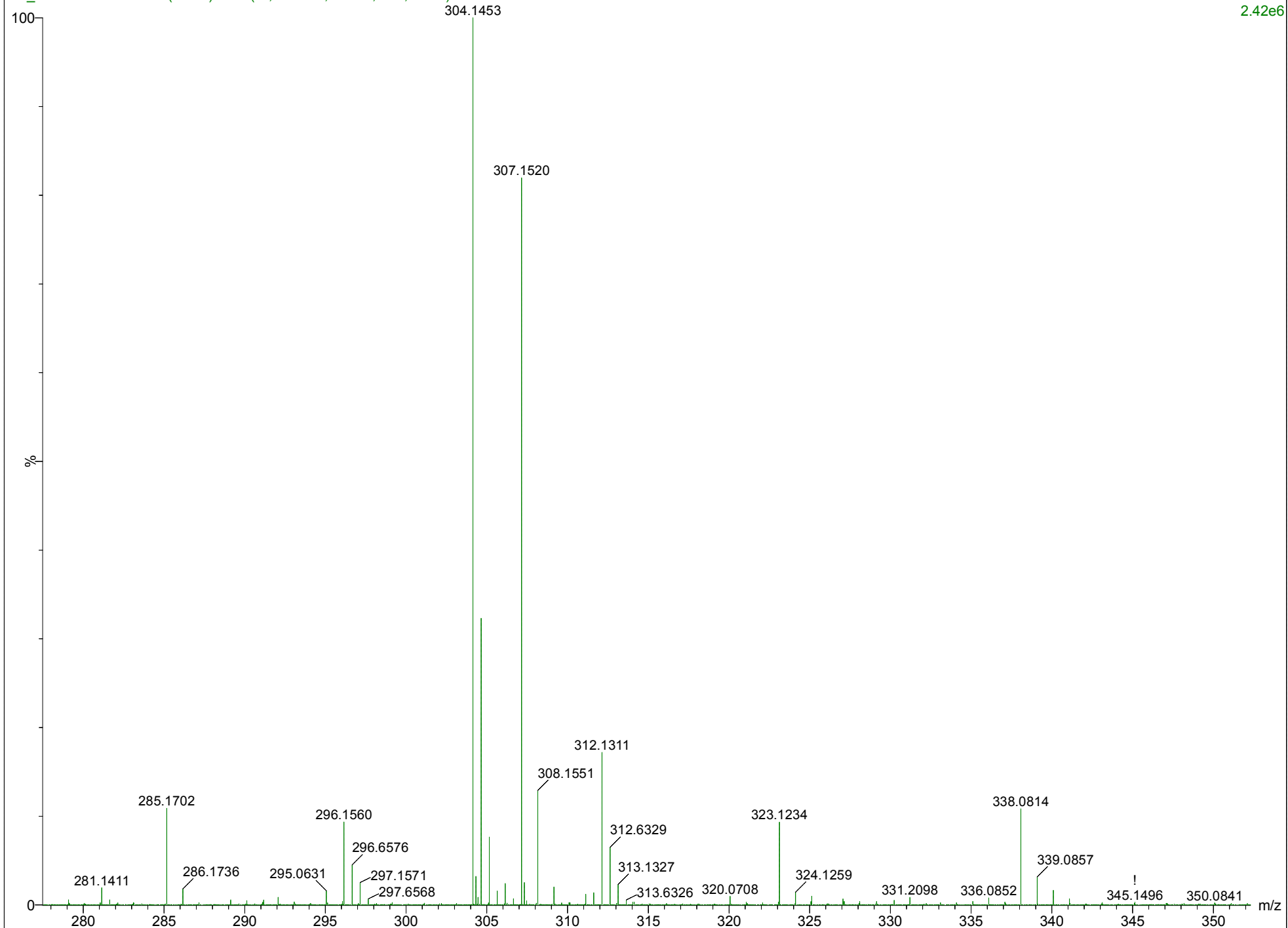

Supplement: Supplementary file 1 [file DataSheet1.ZIP › Supplementary Materials/Figure S66.HR-ESI-MS of Metabolite 18.pdf]

CARBON\_01  
VNS-600 CARBON 1216M11 IN cdcl3 Dec 26 2019

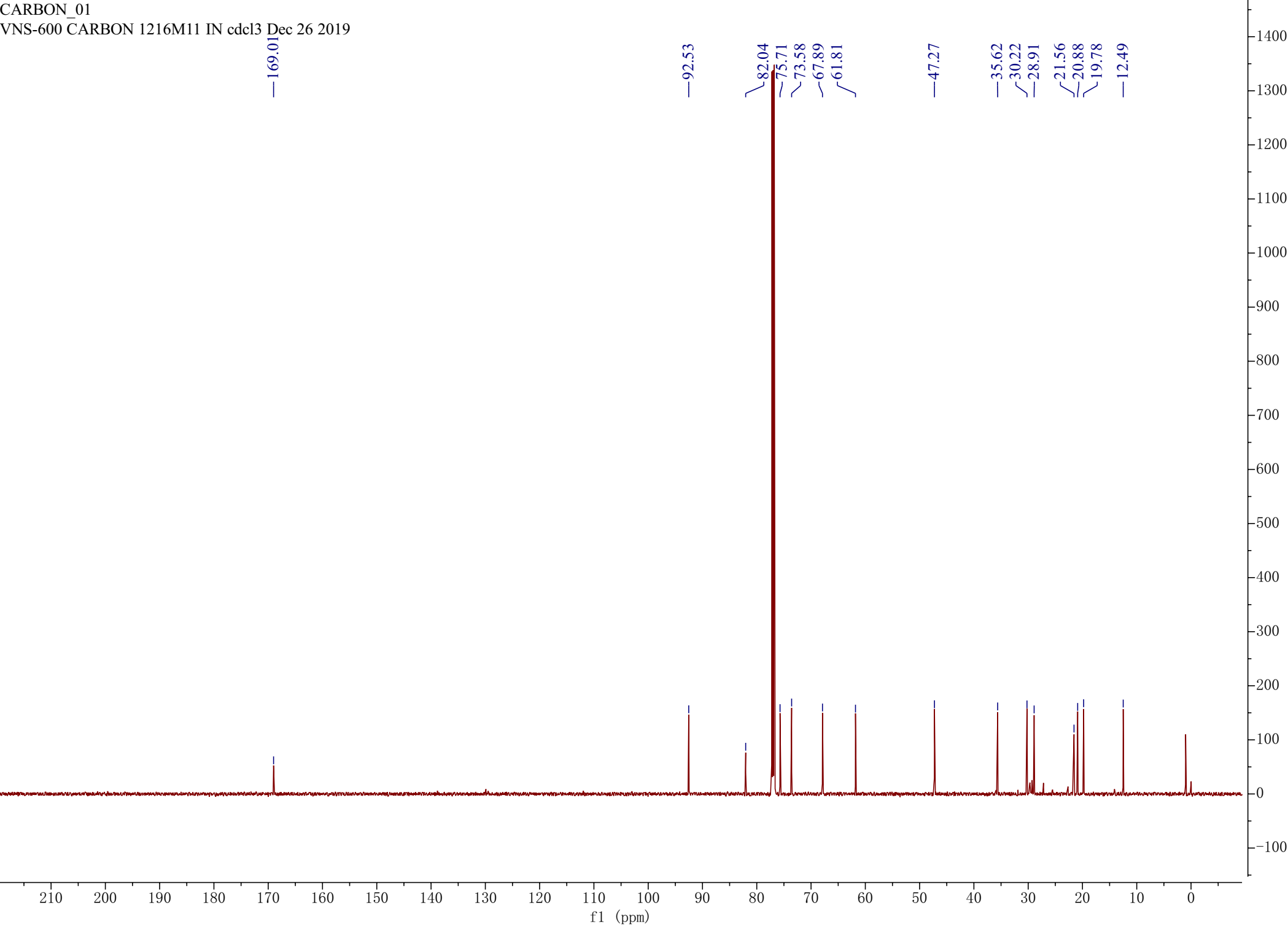

Supplement: Supplementary file 1 [file DataSheet1.ZIP › Supplementary Materials/Figure S7.C-NMR of Metabolite 4.pdf]

PROTON\_01  
VNS-600 PROTON 1216M11 IN ccdl3 Dec 26 2019

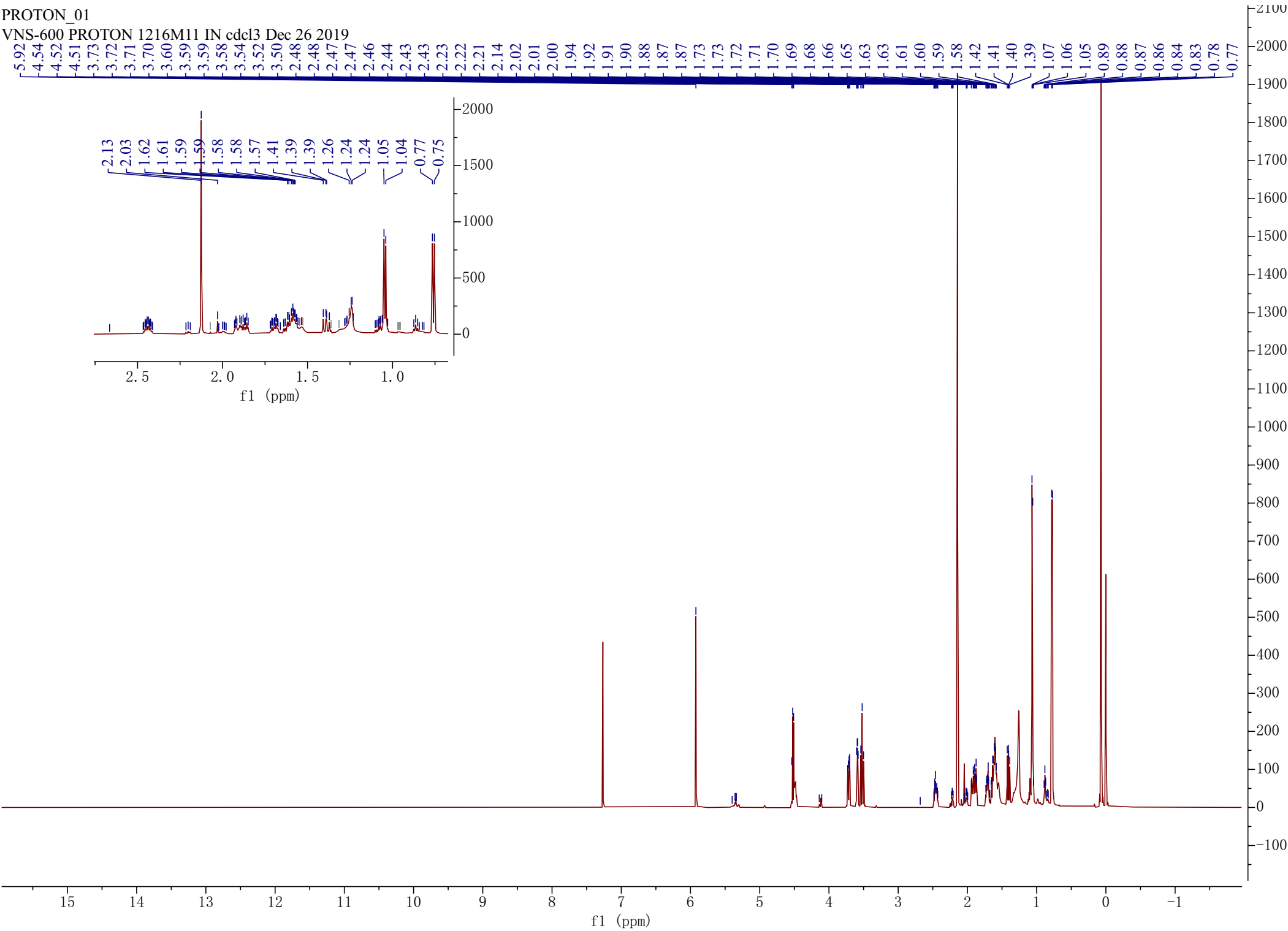

Supplement: Supplementary file 1 [file DataSheet1.ZIP › Supplementary Materials/Figure S8.H-NMR of Metabolite 4.pdf]

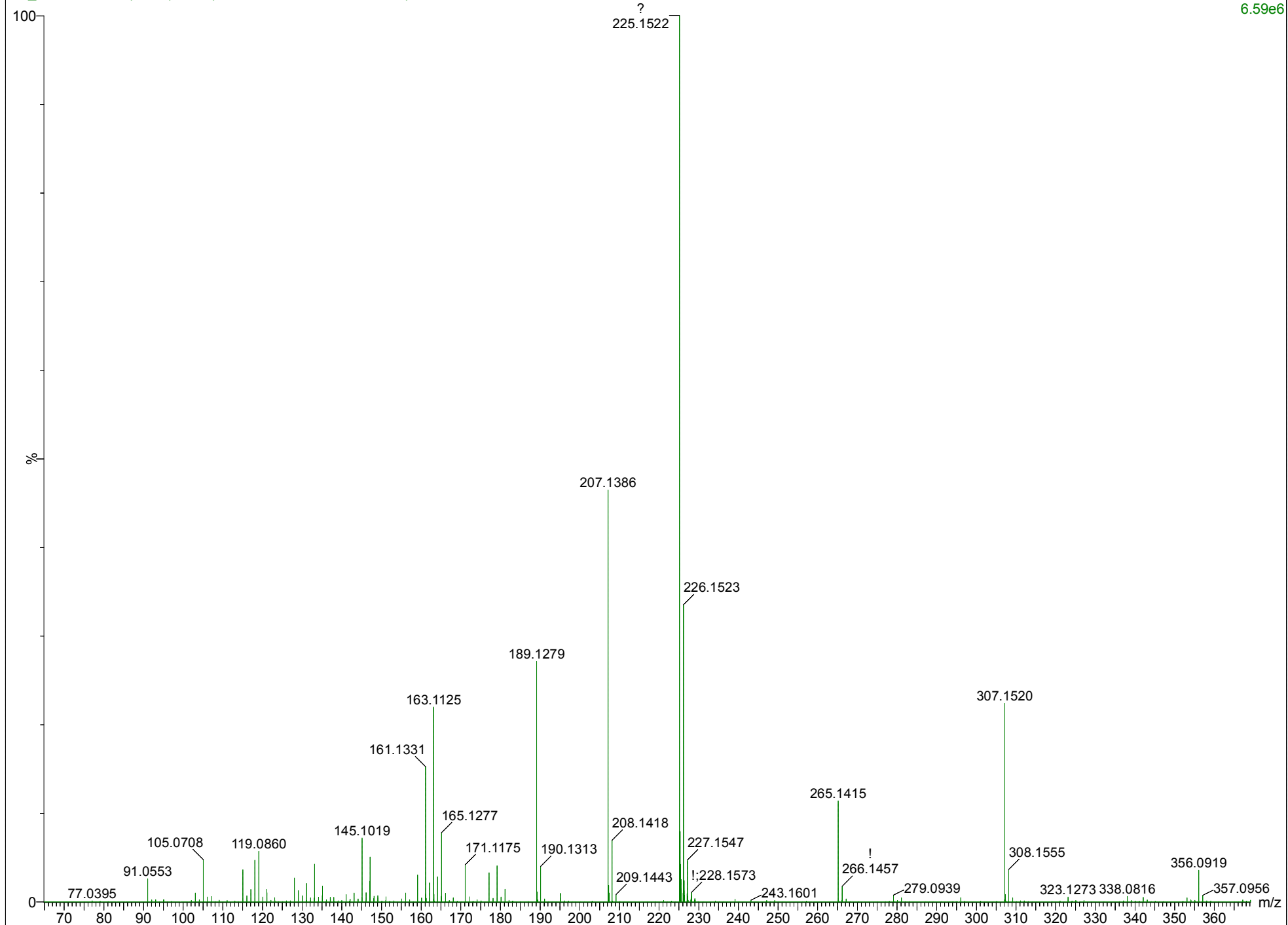

Supplement: Supplementary file 1 [file DataSheet1.ZIP › Supplementary Materials/Figure S9.HR-ESI-MS of Metabolite 4.pdf]
